# Emerging precision medicine in multiple myeloma: clinical and preclinical landscape of T cell, natural killer cell, and macrophages engaging multi-specific antibodies

**DOI:** 10.3389/fimmu.2026.1822508

**Published:** 2026-07-07

**Authors:** Sarra Mestiri, Laila Assami, Zeenath Safira K. M. Yoosuf, Queenie Fernandes, Dina Moustafa Abo El-Ella, Hesham Elsabah, Maysaloun Merhi, Karama Makni-Maalej, Honar Cherif, Said Dermime, Shahab Uddin, Fares Al-Ejeh

**Affiliations:** 1 Qatar Biomedical Research Institute, Hamad Bin Khalifa University, Qatar Foundation, Doha, Qatar; 2 Translational Cancer Research Facility, National Center for Cancer Care and Research, Hamad Medical Corporation, Doha, Qatar; 3 Hamad Bin Khalifa University, Qatar Foundation, Doha, Qatar; 4 Department of Hematology, National Center for Cancer Care and Research, Hamad Medical Corporation, Doha, Qatar; 5 College of Medicine, Qatar University, Doha, Qatar; 6 National Center for Cancer Care and Research, Hamad Medical Corporation, Doha, Qatar; 7 College of Health Sciences, Qatar University, Doha, Qatar; 8 Translational Research Institute, Academic Health System, Hamad Medical Corporation, Doha, Qatar

**Keywords:** multi- specific antibodies, multiple myeloma, NK cell engager, macrophage engager, T cell engager, resistance, biomarkers, combination therapy

## Abstract

Multiple Myeloma (MM) is the third most common hematological malignancy worldwide. Despite advancements in available therapies, MM remains incurable for most patients, mainly due to the early relapse and eventual resistance to therapy, underlining the need for novel drugs. Among these, multi-specific antibodies (MsAbs) have emerged as promising agents. Multi-specific immune cell-engaging antibodies, such as bi-specific, and tri-specific are designed to recognize two or more antigens on the same or distinct cells. These antibodies could promote cancer cell clearance by engaging both myeloma cells and cytotoxic immune cells such as T and Natural killer (NK) cells and macrophages (M). Currently, four bi-specific T cell engagers (teclistamab, elranatamab, talquetamab and linvoseltamab) are approved for refractory or relapsed MM patients (RRMM). While these therapies have demonstrated promising results in achieving deep remissions in RRMM, primary resistance occurs in about one-third of patients. Initially, immune engager research focused only on T cells due to their crucial anti-cancer role, but more recently, interest has expanded to NK cells and M for broader therapeutic potential. To date, several NK and M engaging MsAbs with mainly bi-specific and tri-specific formats are in clinical or preclinical evaluation for improving MM patients’ response and reduce treatment related toxicity. This review discusses the recent advancement in T, NK and M engaging MsAbs in treating MM, including immunological background, mechanisms of action, relevant clinical and preclinical advancements and challenges. Moreover, we discuss the advantages and drawbacks of the different immune cell engagers. Furthermore, we review recent studies investigating the clinical and molecular determinants of resistance along with the latest predictive or prognostic biomarkers of response to MsAbs. Finally, we explore novel strategies to enhance MsAbs efficacy and reduce their toxicity, providing long-term disease control and improving survival in MM patients.

## 1 Background

Multiple Myeloma (MM) is a B cell malignancy characterized by a clonal proliferation of malignant plasma cells in the bone marrow (1). MM is the third most common hematological malignancy worldwide (2). In 2022, approximately 188,000 people were diagnosed with MM with global myeloma mortality amounting to 121,000 cases, and those estimates are predicted to increase by 71% for incidence and 79% for mortality by 2045 (3). Although the introduction of novel therapies in the last decade has improved patients’ survival, MM remains incurable for most patients, mainly due to the early relapse and eventual resistance to therapy, underlining the need for new therapeutic approaches for this malignancy (4, 5).

Immune dysregulation is a hallmark of MM progression (6–8). Although MM is predominantly a B cell lineage disorder, dysfunction of other immune cells, such as T lymphocytes, natural killer (NK), and macrophages (M), along with the activation of the immunosuppressive cells, was observed in relapsed/refractory MM (RRMM) patients (6–8). Consequently, there is a growing interest in developing novel immunotherapeutic strategies to enhance the host immune system and overcome the tumor immunosuppression mechanisms. Multi-specific immune cell-engaging antibodies such as bi-specific (BsAbs), tri-specific (TsAbs), and tetra-specific antibodies are designed to recognize two or more antigens on the same or distinct cells (9). These constructs were designed to overcome resistance to conventional monospecific therapies and avoid immune escape mechanisms (10). Compared to other immunotherapies including chimeric antigen receptor (CAR)-T cell therapy and monoclonal antibodies, MsAbs offer the advantage of their off-the-shelf availability as well as superior clinical efficacy in treating RRMM compared to monoclonal antibodies (11, 12).

Currently, four bi-specific T cell engagers (teclistamab, elranatamab, talquetamab and linvoseltamab) have been approved for RRMM patients. For a long time, research on immune cell engagers has focused exclusively on T lymphocytes, probably due to their crucial role in the anti-cancer immune response. However, researchers are showing an increasing interest in targeting other immune cells, including NK, M and dendritic cells which may offer similar efficacy to T cell engagers with tolerable side effects (13).

This review discusses the recent advancement in T, NK and M engaging MsAbs in treating MM, including immunological background, mechanisms of action, relevant clinical and preclinical advancements and challenges. Moreover, we discuss the advantages and drawbacks of the different immune cell engagers. Furthermore, we review recent studies investigating the clinical and molecular determinants of resistance along with the latest predictive or prognostic biomarkers of response to MsAbs. Finally, we explore novel strategies to enhance MsAbs efficacy and reduce their toxicity, providing long-term disease control and improving survival in MM patients.

## 2 T cell-engaging multi-specific antibodies in multiple myeloma

**Figure 1:**
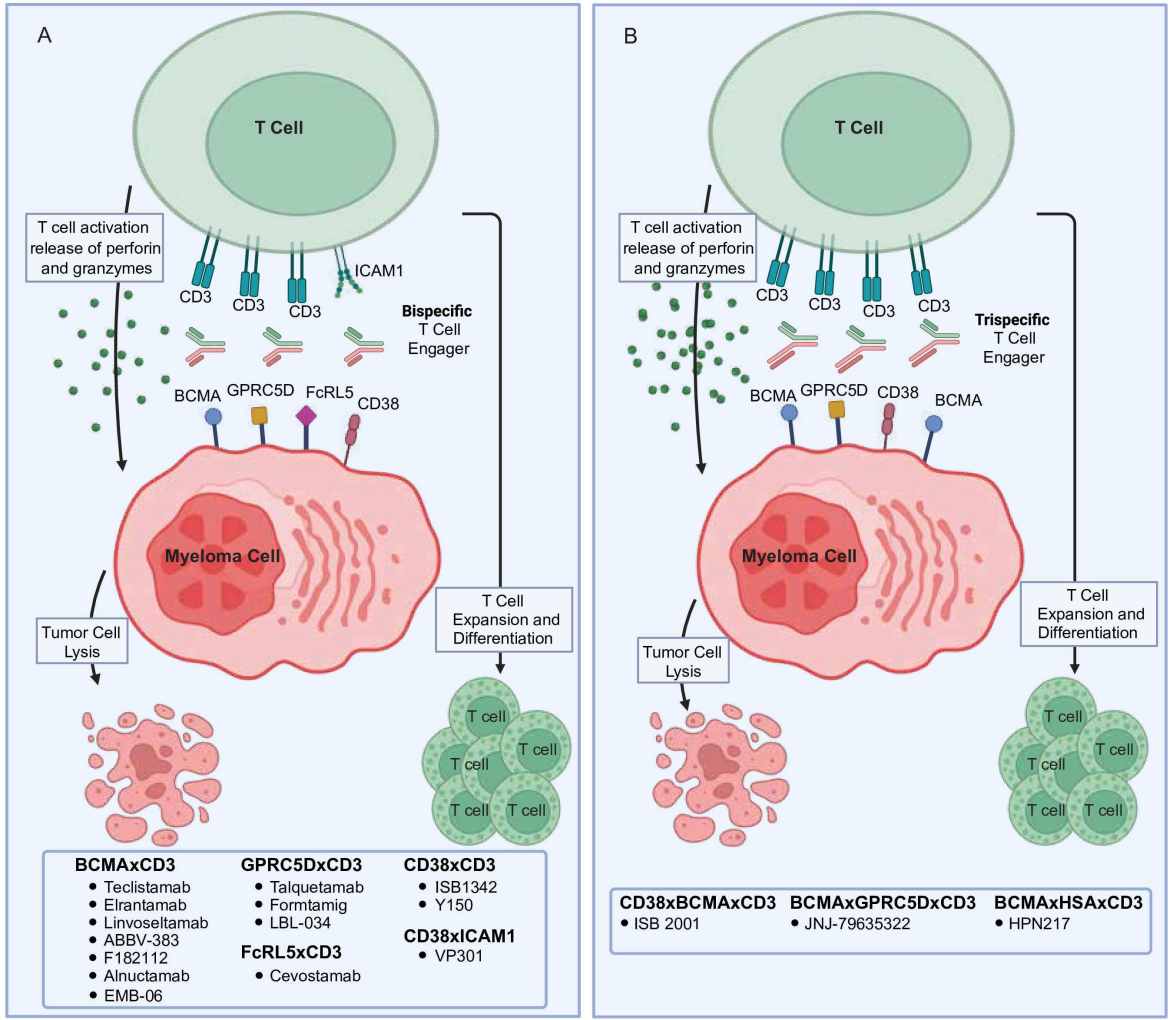
Mechanism of action of bi-specific and tri-specific T cell engagers for multiple myeloma. Bi-specific and tri-specific antibodies bridge immune cells and myeloma cells to form an immunological synapse, thereby initiating various mechanisms to eradicate tumor cells through the release of perforin and granzymes, resulting in the lysis of multiple myeloma cells. **(A)** Bi-specific T cell engagers bind BCMA/GPRC5D/FCRL5/CD38 on myeloma cell and CD3 or ICAM1 on T cell to initiate T cell-dependent cytotoxicity. **(B)** Tri-specific T cell engagers bind BCMA/GPRC5D/CD38 on myeloma cell and CD3 on T cell to initiate T cell-dependent cytotoxicity.

T cell engaging (TCE) MsAbs are engineered antibodies designed with two (bi-specific Ab) or more (tri or tetraspecific Ab) binding sites to simultaneously engage specific tumor antigens on cancer cells and surface receptors on T cells (CD3 or other co-receptors). The dual engagement of T cells and myeloma cells by TCE creates an immunological synapse, causing T cell activation, degranulation, proliferation, and differentiation, leading to MM cell lysis through perforin and granzyme B (**Figure 1**) (14, 15). Recently, the Food and Drug Administration (FDA) has approved the use of teclistamab, elranatamab, talquetamab, and linvoseltamab for the treatment of RRMM patients with at least four prior treatments (16). Furthermore, the safety and efficacy of several other bi- or tri-specific T cell engagers are currently under evaluation, with the potential to be approved shortly, given their promising results (**Table 1**). To our knowledge, there have been no reports of tetra-specific antibodies for the treatment of MM. However, the use of tetra-specific TCE format is being explored in other cancers, including non-Hodgkin lymphoma, acute lymphoblastic leukemia, breast, and pancreatic cancers (59, 60). Here, we describe TCE ongoing clinical trials (**Table 1**), and we review the TCE in preclinical stages, including their *in vitro* and *in vivo* results.

**Table 1:** Clinical trials of multispecific immune cell-engaging antibodies monotherapy in multiple myeloma based on ClinicalTrials.gov.

Clinical trial identifier	Status	Phase	Interventions	Target tested	Disease	Study objectives	Enrollment	Published results
NCT03145181	Active, not recruiting	1	Teclistamab	CD3-BCMA	RRMM	Identify the recommended Phase 2 dose(s) (RP2Ds), and schedule assessed to be safe for teclistamab and to characterize the safety and tolerability of teclistamab at the RP2Ds.	302	(14, 17–29)
NCT06062537	Recruiting	NA	Teclistamab	CD3-BCMA	RRMM	Analyze the effectiveness and Safety of Teclistamab in RRMM.	200	
NCT05945524	Recruiting	NA	Teclistamab	CD3-BCMA	Teclistamab resistant MM	Discover the immune and oncogenomic features that distinguish patients who respond to teclistamab.	100	
NCT05572515	Recruiting	3	Teclistamab	CD3-BCMA	RRMM	Compare the efficacy of teclistamab versus Pomalidomide, Bortezomib, Dexamethasone or Carfilzomib, and characterize the safety and efficacy of an alternative dosing for teclistamab.	614	
NCT04696809	Active, not recruiting	1/2	Teclistamab	CD3-BCMA	RRMM	Evaluate the safety, tolerability and efficacy in Japanese participants with RRMM at the recommended Phase 2 dose identified in phase 1 trial (NCT03145181).	40	(22)
NCT06359067	Completed	NA	Teclistamab or Elranatamab	CD3-BCMA	Refractory MM	Analyze survival data in patients treated with BsAb, as well as safety data, in particular the proportions and locations of infectious events.	600	
NCT04557098	Active, not recruiting	2	Teclistamab	CD3-BCMA	RRMM	Evaluate the efficacy of teclistamab at the recommended Phase 2 dose.	194	(17–20, 22–27, 30–34)
NCT07030517	Recruiting Completed	4	Teclistamab	CD3-BCMA	RRMM	Evaluate the safety of teclistamab in RRMM Indian participants and demonstrated disease progression on the last therapy.	75	
NCT06251076	Not yet recruiting Completed	4	Teclistamab	CD3-BCMA	RRMM	Pilot to develop an outpatient-based process for the administration of teclistamab and evaluate the burden on caregivers.	15	
NCT06285318	Recruiting Completed	NA	Teclistamab or talquetamab	CD3-BCMA CD3-GPRC5D	RRMM	Evaluate clinical outcomes in RRMM patients treated with teclistamab or talquetamab outside of clinical trials.	900	
NCT06505369	Recruiting Completed	2	Talquetamab and Teclistamab	CD3-BCMA CD3-GPRC5D	NDMM	Measure the MRD negativity after talquetamab and teclistamab consolidation in sequence as part of first line treatment in transplant.	50	
NCT06758375	Recruiting Completed	2	Teclistamab	CD3-BCMA	NDMM	Determine the efficacy and safety of low-dose, limited-duration teclistamab as a consolidation scheme in newly diagnosed multiple myeloma patients.	10	(29)
NCT05469893	Recruiting Completed	2	Teclistamab	CD3-BCMA	High-Risk SMM	Test teclistamab compared to lenalidomide + dexamethasone combination in high risk smoldering multiple myeloma.	52	
NCT06425991	Active, not recruiting	1	Teclistamab	CD3-BCMA	RRMM	Compare the pharmacokinetics between teclistamab made from the current and new commercial manufacturing process.	108	
NCT06477783	Recruiting	NA	Teclistamab	CD3-BCMA	RRMM	Assess the clinical efficacy and safety of teclistamab.	100	
NCT05972135	Recruiting	2	Teclistamab or Talquetamab	CD3-BCMA CD3-GPRC5D	MM	Evaluate the outpatient administration of Teclistamab or Talquetamab in MM patients	100	
NCT05932680	Recruiting	2	Teclistamab	CD3-BCMA	RRMM	Determine if stopping treatment after achieving a good response is non-inferior to continuous treatment and reduce the risks of infections and BCMA-negative relapses. A subset of patients will also be part of a biomarker study to explore these factors further.	75	
NCT06285318	Recruiting	N/A	Teclistamab or Talquetamab	CD3-BCMA CD3-GPRC5D	RRMM	Describe the use of teclistamab or talquetamab in the treatment of patients with RRMM outside of clinical trials.	900	
NCT06359067	Completed	N/A	Teclistama or Elranatamab	CD3-BCMA	Refractory MM	Analyze survival data in patients treated with BsAb, as well as safety data, in particular the proportions and locations of infectious events.	600	
NCT03399799	Active, not recruiting	1	Talquetamab	CD3-GPRC5D	RRMM	Characterize the safety of talquetamab and determine the recommended Phase 2 dose(s)	279	(35–41)
NCT04634552	Recruiting	2	Talquetamab	CD3-GPRC5D	RRMM	Evaluate the efficacy and safety of talquetamab at the recommended phase 2 dose(s).	510	(36, 38–44)
NCT04773522	Active, not recruiting	1	Talquetamab	CD3-GPRC5D	RRMM	Evaluate the safety and tolerability in Japanese participants with RRMM at the recommended Phase 2 dose identified in NCT03399799 study.	15	
NCT06066346	Recruiting	2	Talquetamab	CD3-GPRC5D	RRMM	Evaluate whether talquetamab is an effective treatment after BCMA CAR T cell therapy in RRMM patients	17	
NCT05231629	Recruiting	2	Teclistamab	CD3-BCMA	NDMM	Determine the proportion of patients with the lowest minimal residual disease (MRD) response after receiving 6 cycles of treatment.	300	
NCT06592222	Completed	N/A	Elranatamab	CD3-BCMA	RRMM	Compare elranatamab (PF-06863135) with standard-of-care used in real-world clinical practice. Elranatamab used as per phase 2 clinical trial (MagnetisMM-3).	4	
NCT04649359	Active, not recruiting	2	Elranatamab	CD3-BCMA	RRMM	Evaluate whether single-agent Elranatamab (PF-06863135) can provide clinical benefit in RRMM	187	(45–52)
NCT06057402	Recruiting	4	Elranatamab	CD3-BCMA	MM	A post-trial access (PTA) open-label, single-arm study in MM participants who continue to derive clinical benefit from elranatamab monotherapy.	80	
NCT05932290	Completed	N/A	Elranatamab	CD3-BCMA	RRMM	Compare elranatamab with standard-of-care (SOC) therapies used in real-world clinical practice.	514	(53)
NCT05014412	Active, not recruiting	2	Elranatamab	CD3-BCMA	RRMM	Evaluate the safety (in particular the rate of Grade ≥ 2 CRS) of a step-up priming dose regimen of Elranatamab in participants with RRMM.	86	(48, 49, 52)
NCT05228470	Active, not recruiting	1/2	Elranatamab	CD3-BCMA	RRMM	Understand how elranatamab, acts as potential treatment for refractory MM in Chinese participants.	39	
NCT05565391	Completed	N/A	Elranatamab	CD3-BCMA	RRMM	Understand how well elranatamab may be used for RRMM by determining the ORR-unweighted analysis and compared to ORR from standard of care in real-world.	508	
NCT06504524	Completed	N/A	Elranatamab	CD3-BCMA	RRMM	Understand how well elranatamab may be used for RRMM compared to standard-of-care therapies in Germnay and the US.	633	
NCT04798586	Completed	1	Elranatamab	CD3-BCMA	RRMM	Confirm the safety and tolerability of elranatamab in Japanese participants with RRMM.	4	(49, 52, 54)
NCT06479954	Active, not recruiting	N/A	Elranatamab	CD3-BCMA	RRMM	Assess the safety and efficacy in patients with RRMM treated with elranatamab under the actual use.	1	
NCT06207799	Recruiting	2	Elranatamab	CD3-BCMA	High-Risk Multiple Myeloma	Determine the overall proportion of high-risk MM participants achieving sustained MRD-negative CR status after the completion of the treatment plan including *in-vivo* purging with elranatamab, auto-HCT, and post-transplant maintenance therapy.	40	
NCT06483100	Recruiting	2	Elranatamab	CD3-BCMA	NDMM	Generate efficacy data for a personalized maintenance approach using bone marrow based MRD testing (clonoSEQ) to guide post-AHCT maintenance with elranatamab for this patient population.	65	
NCT06152575	Recruiting	3	Elranatamab	CD3-BCMA	RRMM	Compare elranatamab to other medicines for the treatment of MM.	492	
NCT06282978	Recruiting	2	Elranatamab	CD3-BCMA	RRMM	Effectiveness and safety of elranatamab for patients with RRMM.	50	
NCT06421675	Recruiting	2	Elranatamab	CD3-BCMA	RRMM	Improve the tolerability and safety of elranatamab in patients with RRMM by evaluating an outpatient and intermittent dosing strategy.	40	
NCT06015542	Recruiting	2	Elranatamab	CD3-BCMA	Relapsed MM	Test the self-administration of elranatamab	20	
NCT05317416	Recruiting	3	Elranatamab	CD3-BCMA	NDMM	Evaluate whether elranatamab monotherapy can provide clinical benefit compared to lenalidomide monotherapy in participants with newly diagnosed multiple myeloma after autologous stem cell transplant.	811	
NCT06183489	Recruiting	2	Elranatamab	CD3-BCMA	High-Risk SMM	Determine the efficacy of elranatamab in patients with previously untreated high-risk SMM.	50	
NCT06581848	Not yet recruiting	N/A	Elranatamab	CD3-BCMA	RRMM	Assess safety and effectiveness of elranatamab in the real-world clinical setting in patients with MM in Korea.	150	
NCT06711705	Recruiting	2	Elranatamab	CD3-BCMA	RRMM	Efficacy of elranatamab alone in patients with RRMM who have previously received 1 to 3 combinations of treatment. Also, evaluate the MRD negativity rate as best response as primary outcome measures, ORR, PFR and safety.	33	
NCT06138275	Recruiting	2	Elranatamab	CD3-BCMA	RRMM	Test if elranatamab reduces the risk of disease progression after idecabtagene vicleucel in RRMM. Adverse events will be evaluated as primary outcome measures. ORR, OS and MRD will be evaluated as secondary outcome measures.	32	
NCT04910568	Active, not recruiting	1	Cevostamab	CD3-FCRL5	RRMM	Evaluate the safety, tolerability, pharmacokinetics, and pharmacodynamics of cevostamab monotherapy, cevostamab plus pomalidomide and dexamethasone or cevostamab plus daratumumab and dexamethasone.	126	
NCT05801939	Recruiting	2	Cevostamab	CD3-FCRL5	RRMM	Assess the impact of cevostamab consolidation post-BCMA CAR-T cell therapy on the rate of MRD-negative complete remission at 12 months	30	
NCT03275103	Active, not recruiting	1	Cevostamab	CD3-FCRL5	RRMM	Evaluate the safety and pharmacokinetics of escalating doses of cevostamab	355	
NCT05535244	Active, not recruiting	1/2	Cevostamab	CD3-FCRL5	RRMM	Evaluate the efficacy, safety, and pharmacokinetics of cevostamab	90	
NCT06376526	Recruiting	2	Linvoseltamab	CD3-BCMA	NDMM	Determine whether linvoseltamab therapy in patients with newly diagnosed MM will convert the disease status from MRD-positive to MRD-negative and increase the length of time that the disease is controlled.	28	
NCT05828511	Recruiting	1/2	Linvoseltamab	CD3-BCMA	NDMM	Safety, tolerability, and effectiveness of linvoseltamab.	149	
NCT03761108	Recruiting	1/2	Linvoseltamab	CD3-BCMA	RRMM	Safety and dosage, and efficacy of linvoseltamab.	387	(55, 56)
NCT06140524	Recruiting	2	Linvoseltamab	CD3-BCMA	High-Risk Monoclonal Gammopathy SMM	Safety and effectiveness of linvoseltamab in High-Risk Monoclonal Gammopathy of Undetermined Significance and Non-High-Risk Smoldering MM.	116	
NCT05730036	Recruiting	3	Linvoseltamab	CD3-BCMA	RRMM	Safety and effectiveness oflinvoseltamab compared to a combination of elotuzumab, pomalidomide and dexamethasone, (called EPd) in participants after treatment with lenalidomide and (for some) a CD38 antibody.	410	
NCT05650632	Recruiting	1	ABBV-383	CD3-BCMA	RRMM	Evaluate dose optimization measures and safety of ABBV-383	210	
NCT06223516	Recruiting	1	ABBV-383	CD3-BCMA	RRMM	Assessing adverse events and clinical activity with subcutaneous Injection	60	
NCT05286229	Active, not recruiting	1	ABBV-383	CD3-BCMA	RRMM	Assess the adverse events and change in disease state of ABBV-383 in adult participants with RRMM.	8	
NCT04735575	Recruiting	1/2	EMB-06	CD3-BCMA	RRMM	Safety, tolerability, and identify the maximum tolerated dose and/or recommended Phase 2 dose	44	
NCT04984434	Unknown status	1	F182112	CD3-BCMA	RRMM	Evaluate the safety and tolerability of F182112 when infused intravenously and determine the MTD and/or the recommended Phase 2 dose (RP2D) of F182112 when infused IV.	68	
NCT03486067	Terminated	1	CC-93269	CD3-BCMA	RRMM	Dose escalation and first-in-human clinical study of CC-93269.	183	
NCT04557150	Active, not recruiting	1	Forimtamig	CD3-GPRC5D	RRMM	Dose-escalation and dose expansion study of forimtamig	225	(57)
NCT06049290	Recruiting	1/2	LBL-034	CD3-GPRC5D	RRMM	Evaluate safety, tolerability, pharmacokinetics, and efficacy of LBL-034 in patients with RRMM.	342	
NCT03309111	Completed	1	ISB 1342	CD3-CD38	RRMM	Assess safety, efficacy, pharmacokinetic, pharmacodynamic, and immunogenicity with ISB 1342	81	
NCT04184050	Active, not recruiting	1	MK-4002	CD3-HSA-BCMA	RRMM	Safety and tolerability of different doses of MK-4002.	100	
NCT04401020	Active, not recruiting	1	SAR442257	CD38-CD3-CD28	RRMM RR-NHL	Determine the maximum tolerated dose of SAR442257 administered as a single agent.	47	
NCT03309111	Completed	1	ISB 1342	CD3-CD38	RRMM	Assess safety, efficacy, pharmacokinetic, pharmacodynamic, and immunogenicity with ISB 1342	81	
NCT05011097	Unknown status	1	Y150	CD3-CD38	RRMM	Safety and tolerability of Y150 at different dose levels and find recommended dose for Phase II/III.	20	
NCT05652335	Recruiting	1	JNJ-79635322	CD3-BCMA-GPRC5D	RRMM Previously Treated Amyloid Light-chain (AL) Amyloidosis	Identify the recommended phase 2 dose and schedule(s) for JNJ-79635322	180	
NCT04401020	Active, not recruiting	1	SAR442257	CD38-CD28- CD3	RRMM	Safety, tolerability, and pharmacokinetics of SAR442257 in and to determine the recommended Phase 2 dose(s).	47	
NCT04184050	Active, not recruiting	1	HPN217 (MK-4002)	CD3-HSA-BCMA	RRMM	Safety, tolerability, and pharmacokinetics of HPN217.	100	
NCT05862012	Recruiting	1	ISB 2001	CD3-CD38-BCMA	RRMM	Safety and anti-myeloma activity of ISB 2001	200	
NCT04434469	Completed	1	RO7297089	CD16-BCMA	RRMM	Safety, tolerability, and pharmacokinetics of RO7297089 and preliminary assessment of anti-tumor activity	27	(58)
NCT05839626	Terminated	1/2	SAR445514	NKp46-CD16-BCMA	RRMM Light-chain Amyloidosis	Safety of SAR445514 and determine the recommended Phase 2 dose(s) in RRMM patients.	32	
NCT05427812	Terminated	1/2	ISB 1442	CD38-CD47	RRMM	Safety and efficacy of ISB 1442 in RRMM.	29	

RRMM, Relapsed Refractory Multiple Myeloma; MM, Multiple Myeloma; BCMA, B cell Maturation Antigen; GPRC5D, G protein–coupled receptor class C group 5 member D; NDMM, Newly Diagnosed Multiple Myeloma; RP2Ds, Recommended Phase 2 dose(s); SMM, Smoldering Multiple Myeloma; VGPR, Very Good Partial Response; BsAb, Bispecific Antibody; MRD, Minimal Residual Disease; RR-NHL, Relapsed Refractory Non-Hodgkin Lymphoma; PTA, Post-Trial Access; SOC, Standard of Care; PFS, Progression Free Survival; ORR, Objective Response Rate; CRS, Cytokine Release Syndrome; DLTs, Dose Limiting Toxicities; ICANS, Neurotoxicity Syndrome; AHCT, Autologous Hematopoietic Cell Transplant; MFDS, Ministry of Food and Drug Safety; IMWG, International Myeloma Working Group Response Criteria.

### 2.1 Clinical applications of bi-specific T cell engagers

#### 2.1.1 Bi-specific T cell engagers targeting B cell maturation antigen

B-cell maturation antigen (BCMA) is a member of the Tumor Necrosis Factor Receptor Superfamily (TNFRSF) 17 that is expressed mainly in mature B lymphocytes (61). The binding of BCMA to its ligands, B cell activating factor (BAFF) and proliferation-inducing ligand (APRIL), promotes B cell proliferation and differentiation (14). BCMA was reported to be highly expressed at the mRNA and protein levels in MM cells (62). This expression pattern has contributed to making BCMA an attractive target for MM treatments.

##### 2.1.1.1 Teclistamab

Teclistamab is the first anti-BCMAxCD3 BsAb to be approved for the treatment of RRMM patients. Preclinical studies for teclistamab showed promising anti-tumor activity in various MM models, including MM cell lines, ex vivo MM samples, and MM xenografts (14). Importantly, teclistamab-mediated cytotoxicity was not altered in the presence of soluble BCMA (sBCMA) at concentrations similar to those detected in MM patients (14). In subsequent MajesTEC-1 phase 1/2 trials (NCT03145181 and NCT04557098), 165 refractory or relapsed MM patients to at least three therapy lines were enrolled. At 14.1- and 30.4-month follow-up, teclistamab treatment achieved an overall response rate (ORR) of 63%, with a rate of a complete response or better, increasing from 39.4 to 46.1%, respectively (17, 63). However, Firestone et al. reported a lower efficacy in patients with prior anti-BCMA therapy (ORR 50%), suggesting that exposure to multiple prior anti-BCMA therapies could diminish teclistamab efficacy (64). The minimal residual disease (MRD) negative rate among responder patients in the MajesTEC-1 trial was 26.7% at 18.4 months follow-up. Common adverse events to teclistamab included cytokine release syndrome (CRS) (in 72.1% of the patients; grade 3, 0.6%; no grade 4), neutropenia (in 70.9%; grade 3 or 4, 64.2%), anemia (in 52.1%; grade 3 or 4, 37.0%), and thrombocytopenia (in 40.0%; grade 3 or 4, 21.2%), infections (in 76.4%; grade 3 or 4, 44.8%) and immune effector cell–associated neurotoxicity syndrome (ICANS) (3.0%; all grade 1 or 2) (17). These outcomes played an important role in the FDA approval of this treatment in 2022 (16). Importantly, teclistamab demonstrated improved effectiveness compared to other real-world physicians’ choice of therapy, ranging between 2.3-fold and 148.3-fold increase in ORR (30). Collectively, these studies highlight the clinical benefit of teclistamab as a novel and effective treatment for RRMM patients. A meta-analysis of five studies, including 661 RRMM patients treated with teclistamab, showed similar efficacy data as shown in the MajesTEC-1 study, with a pooled ORR of 62.8% and a low incidence of CRS and neurotoxicity (65). Moreover, the MajesTEC-3 trial investigated the efficacy of combining teclistamab with a daratumumab-based regimen compared to a daratumumab-based regimen alone. The on-tumor toxicity effect of daratumumab, an anti-CD38 monoclonal antibody, as well as its ability to deplete immunosuppressive cells while enhancing CD8^+^ T cell cytotoxicity, has suggested its combination with teclistamab as a potentially potent therapeutic strategy. Teclistamab-daratumumab combination compared to daratumumab alone improved ORR (89.0% versus 75.3%), the complete response or better rates (81.8% versus 32.1%), and minimal residual disease negativity (58.4% versus 17.1%). Serious adverse events were reported in 70.7% of patients in the combination therapy group and 62.4% in the daratumumab alone (NCT05083169) (66). Currently, a phase II clinical trial (NCT06758375) is investigating the efficacy of low dose teclistamab in newly diagnosed MM (NDMM).

##### 2.1.1.2 Elranatamab

Elranatamab, an anti-BCMAxCD3 BsAb, showed promising therapeutic efficacy in phase 1 (NCT03269136) and phase 2 (NCT04649359) trials of the MagnetisMM-3 study. The phase 2 trial included 123 RRMM patients without prior BCMA-directed therapy to receive at least six cycles of elranatamab. At 14.7- and 28.4- month median follow-up, the ORR remained consistent at 61.0%, with an increased rate of patients achieving a complete response or better from 35.0% to 37.4%, respectively (45, 67). The most common adverse events to elranatamab included infections (69.9%), CRS (57.7%), anemia (48.8%), and neutropenia (48.8%). Grade 3–4 toxicities were reported in 39.8% of infections, 37.4% of anemia, 48.8% of neutropenia, and were not observed in CRS (45). Importantly, the same study reported that biweekly dosing of elranatamab may improve long-term safety without compromising efficacy (45). Importantly, Bahlis et al. demonstrated that a prior BCMA-directed therapy did not significantly affect the efficacy of elranatamab, where 53.8% of the patients achieved a response (68).

##### 2.1.1.3 Linvoseltamab

Linvoseltamab is a BsAb designed to target BCMA on myeloma cells and CD3 on T cells (55). Cryo-electron microscopy analysis of linvoseltamab Fab fragment bound to the BCMA extracellular domain revealed two binding sites: 16 BCMA residues, which largely overlap with the reported epitopes of teclistamab and elranatamab, and the N-terminal end of BCMA, suggesting a tilted binding orientation towards the BCMA N-terminus. Unlike other BsAbs, linvoseltamab maintains its binding ability to BCMA on target cells even in the presence of the R27P mutation - a mutation in BCMA which mediates resistance to T cell engagers, and it does not interact with the P33 domain, making it unaffected by P33S mutations, another BCMA mutation affecting T cell engagers (69).

Results from the first-in-human trial of linvoseltamab reported its safety and tolerability in RRMM (70). In the subsequent phase 1/2 LINKER-MM1 clinical trial (NCT03761108), patients treated with 200 mg linvoseltamab demonstrated an ORR of 71%, with 50% of patients achieving a complete response or better at a median follow-up of 14.3 months. The 50 mg treated group demonstrated an ORR of 48%, with 21% achieving a complete response at a median follow-up of 7.4 months (55). Linvoseltamab at 200 mg showed significantly higher response rates compared to the current standard therapies (NCT05673967) (71). Linvoseltamab exhibited comparable efficacy *in vivo* and *in vitro* models to BCMA-targeted CAR-T, but faster anti-tumor kinetics (72). Importantly, long-term follow-up of the LINKER-MM1 study (median of 21.3 months) showed that treatment with 200 mg Linvoseltamab was associated with high response rates and favorable survival outcomes across patients with markers of high disease burden (elevated % bone marrow plasma cells or soluble BCMA) or difficult-to-treat RRMM (including extramedullary plasmacytoma, International Staging System stage 3, and high-risk cytogenetic status), alongside a favorable safety profile (56). Notably, increased linvoseltamab dose resulted in treatment- emergent adverse events in all patients, with 88% classified as grade 3 or higher (56). Currently, linvoseltamab is being evaluated in phase 2 clinical trials on patients with high-risk smoldering MM (NCT05955508) and phase 1/2 clinical trials for newly diagnosed MM (NCT05828511).

##### 2.1.1.4 ABBV-383

ABBV-383 is a BsAb targeting BCMA and CD3 designed with a unique 2 + 1 format featuring two BCMA-binding domains and a low-affinity CD3-binding domain to reduce potential CRS and the negative impact of sBCMA (73, 74). In an ongoing phase 1 dose-escalation trial (NCT03933735), ABBV-383 demonstrated encouraging efficacy, achieving an ORR of 64% in the 40 mg cohort and 60% in the 60 mg cohort, alongside a favorable safety profile (75). Based on these promising results, a phase 3 study was initiated to further evaluate the efficacy of ABBV-383 compared to current standard therapies, exploring its potential as a therapeutic option for RRMM (NCT06158841).

##### 2.1.1.5 Other bi-specific T cell engagers targeting B cell maturation antigen

The safety and efficacy of several other BCMA-targeting bi-specific antibodies, including F182112, alnuctamab, and EMB-06 are currently under investigation. Preliminary data from the F182112 phase 1 study (NCT04984434) reported an ORR of 43.8% at a median follow-up of 3.1 months (76). In early clinical trials, the intravenous administration of alnuctamab was associated with CRS in 76% of patients, with 7% experiencing grade ≥3 CRS. Consequently, the mode of administration was switched to subcutaneous (NCT03486067) (77). Initial data from this trial demonstrated an ORR of 54% across all doses after a median follow-up time of 7.4 months, with an improved safety profile (77). EMB-06, an anti-BCMAxCD3 BsAb designed with a 2:2 tetravalent binding format, also showed a favorable safety profile in a phase 1/2 clinical trial (NCT04735575), achieving an ORR of 39% (71).

#### 2.1.2 Bi-specific T cell engagers targeting G-protein coupled receptor family C group 5 member D

Unlike other targets such as BCMA or CD38, G protein-coupled receptor, class C, group 5, member D (GPRC5D) expression is highly specific to myeloma cells, with minimal expression in normal tissues, making it an attractive therapeutic target for MM. Indeed, GPRC5D expression was reported in plasma cells, including MM cells, as well as cells producing keratin (15, 35). However, its function and ligands are not well characterized (15, 35). Further research is required to unravel the biological function of GPRC5D in MM to optimize the development of GPRC5D-targeted therapies in MM patients.

##### 2.1.2.1 Talquetamab

Talquetamab (JNJ-64407564) is an FDA-approved BsAb that targets GPRC5D on MM cells and CD3 on T cells. Its preclinical efficacy has been demonstrated in GPRC5D-positive cell lines, ex vivo MM samples, and murine models through T cell-mediated cytotoxicity (15, 35). Phase 1/2 MonumenTAL-1 trial, including 537 heavily pretreated RRMM patients, talquetamab resulted in 74% ORR in the 0·4 mg/kg once a week group, 69% in the 0·8 mg/kg every 2 weeks group, and 67% in the previous T cell redirection therapy group (NCT03399799, NCT04634552) (36). Grade 1/2 CRS, taste change, skin and nails related adverse events were the most common, while grade 3–4 adverse events included neutropenia, anemia, and lymphopenia (36, 78). Furthermore, a multicenter retrospective study investigated the efficacy and safety of talquetamab in heavily pretreated RRMM. This study reported an ORR of 73%, of which 26% had a complete response and 26% had a very good partial response. The most common adverse events were CRS (54%), infections (27%), and immune effector cell-associated neurotoxicity syndrome (9.8%) (79).

##### 2.1.2.2 Forimtamig

Forimtamig, another anti-GPRC5DxCD3 BsAb, is designed with a 2 + 1 configuration with two binding sites targeting GPRC5D and one targeting CD3 (57). This design has shown higher potency compared to the 1 + 1 structure, as forimtamig binds GPRC5D with high avidity and promotes a stable immunological synapse. Furthermore, the efficacy of forimtamig was validated in ex vivo models of bone marrow aspirates from newly diagnosed MM patients and NCI-H929 engrafted in a humanized mouse model (57). The first-in-human dose-escalation and expansion clinical trial (NCT04557150) reported an ORR of 71.4% for the intravenous treatment cohort, including 57.1% with very good partial response (VGPR) or more, and an ORR of 60.4% in the subcutaneous cohort, including 39.6% with VGPR or more (80).

##### 2.1.2.3 LBL-034

LBL-034 is an anti-GPRC5DxCD3 BsAb consisting of a 2 + 1 format, which showed promising anti-MM activity in preclinical studies (81) and has progressed to a phase 1 clinical trial (NCT06049290). However, LBL-034 demonstrated potent binding to GPRC5D low-expressing cells and weak binding to T cells *in vitro* (81). Preliminary data from the phase 1 trial (NCT06049290), including 15 patients in dose escalation and expansion cohorts, reported a 50% clinical benefit rate (82).

#### 2.1.3 Bi-specific T cell engagers targeting Fc receptor Like 5

Fc Receptor-Like 5 (FCRL5) is a transmembrane protein expressed exclusively in the B cell lineage and at a higher expression level on MM cells (83). Importantly, MM cells exhibited significantly higher expression of FCRL5 compared to BCMA, with 78.57% of patients expressing FCRL5 on more than 50% of myeloma cells compared to 35.71% for BCMA, suggesting that FCRL5 could be a more attractive target for MM therapies than BCMA (83).

Cevostamab is an anti-FCRL5xCD3 BsAb. A phase 1 study (NCT03275103) of cevostamab monotherapy in heavily pre-treated RRMM reported an ORR of 54.5% for patients treated with a 160 mg dose compared to 36.7% for the 90 mg dose at a median follow-up time of 6.1 months (84). However, cevostamab is associated with high rates of CRS at various grades, affecting 80% of the patients (84). Nevertheless, preliminary data on the duration of response to cevostamab have been encouraging (85). Interestingly, pretreatment with tocilizumab, an anti-interleukin-6 monoclonal antibody, reduced the incidence of CRS in patients receiving cevostamab, indicating the potential of tocilizumab pretreatment to mitigate CRS (86). Interestingly, without compromising efficacy, a single dose of prophylactic tocilizumab resulted in low rates of CRS (10.1%) and ICANS (5.9%) post-treatment with teclistamab, elranatamab, linvoseltamab, and talquetamab (87). Further studies are being conducted to evaluate the efficacy of cevostamab in patients with prior anti-BCMA therapies (NCT05535244) (88).

#### 2.1.4 Bi-specific T cell engagers targeting CD38

ISB 1342, an anti-CD38xCD3 BsAb, reported potent activity in both *in vitro* and *in vivo* models. Interestingly, compared to daratumumab, the anti-CD38 monoclonal antibody ISB 1342 demonstrated higher cytotoxicity to MM cells (89). The efficacy and safety of ISB 1342 are currently under investigation in a phase 1 dose-escalation trial in RRMM patients (NCT03309111). Preliminary data reported that ISB 1342 was well tolerated even at high doses, with a moderate CRS event (90, 91). Another CD38xCD3 BsAb, Y150, is also currently under investigation in the phase 1 clinical trial (NCT05011097). Several other CD38XCD3 targeting BsAb, such as AMG 424, and IGM-2644, showed promising results in preclinical models both *in vitro* and *in vivo* (92–94). However, all clinical trials involving these BsAbs were terminated based on the company or sponsor’s decision (NCT05698888, NCT03445663, and NCT05908396).

### 2.2 Clinical applications of tri-specific T cell engagers

ISB 2001 was developed to target both BCMA and CD38 on tumor cells, along with CD3 on T cells. This design exhibited efficacy in various preclinical models and showed superior cytotoxicity compared to other bi-specific TCEs, including teclistamab, alnuctamab, and EM801 (58). The safety and efficacy of ISB 2001 are currently under evaluation in a phase 1 clinical trial (NCT05862012) for the treatment of RRMM. HPN217 is a tri-specific antibody targeting BCMA, human serum albumin (HSA), and CD3. The addition of the HSA domain extends the TsAb half-life. Interim results from the phase 1 clinical trial (NCT04184050) showed that HPN217 is well-tolerated in RRMM patients (95, 96). Furthermore, targeting both BCMA and GPRC5D on MM cells along with CD3 with JNJ-79635322 showed high anti-MM activity in cells expressing both BCMA and GPRC5D as well as those expressing BCMA or GPRC5D individually. JNJ-79635322 is currently in a phase I clinical trial (NCT05652335) for the treatment of RRMM (97). In addition, phase II (NCT07266441) and III (NCT07258511) clinical trials are currently ongoing with no published data available yet. Furthermore, the efficacy and safety of JNJ-79635322 in combination with teclistamab or talquetamab are being tested in a phase II trial (NCT05695508).

### 2.3 Pre-clinical studies based on bi-specific T cell engagers

Other studies have explored targeting different antigens on myeloma cells to enhance treatment efficacy and limit adverse events. These antigens, including CD138, the signaling lymphocytic activation molecule family member 7 (SLAMF7), CD19, and NY-ESO-1, are still in preclinical evaluation and have not advanced to clinical trials against MM.

CD138 (syndecan-1) is a glycoprotein expressed on bone marrow hematopoietic cells, endothelial cells, and is less expressed on some breast cancer cells (98). Its high expression in myeloma cells was associated with tumor cell proliferation and survival (98). Preclinical studies showed that BsAbs targeting CD138xCD3, such as h-STL002 and m-STL002, exhibit cytotoxic activity against MM cells (99).

Signaling SLAMF7 is a glycoprotein normally expressed on immune cells. A preclinical study compared the activity of monoclonal antibody, CAR T cell, and BsAb targeting SLAMF7 using *in vitro* and *in vivo* models. This study found that the anti-tumor activity was comparable in all three constructs (100).

NY-ESO-1 is a cancer-testis antigen expressed in myeloma cells and is associated with poor prognosis in MM (101). Generally, its expression is limited to the testis, making it an attractive target for tumor cells. A preclinical study of anti-NY-ESO-1xCD3 BsAb demonstrated promising antitumor activity *in vivo* and *in vitro* (102).

### 2.4 Pre-clinical studies based on tri-specific T cell engagers

SLAMF7xCD38xCD3 is a tri-specific antibody designed with hemibody pairs targeting CD38 and SLAMF7, enabling T cells to recognize and lyse, especially the dual antigen-positive MM cells. Preclinically, SLAMF7xCD38xCD3 TsAb eliminated the dual antigen-positive MM cells through T cell activation while leaving the single antigen-positive cells unharmed. Importantly, SLAMF7xCD38xCD3 TsAb demonstrated a reduced off-target toxicity and cytokine release compared to CD38 and SLAMF7 BsAb (103).

SAR442257 is a TsAb simultaneously targeting CD38 on myeloma cells and CD28, CD3 on T cells. This antibody demonstrated more potent cytotoxicity in an ex vivo model of relapsed MM compared to CD38xCD3 BsAbs and anti-CD38 monoclonal antibodies (104).

## 3 NK and M cell-engaging multi-specific antibodies in multiple myeloma

**Figure 2:**
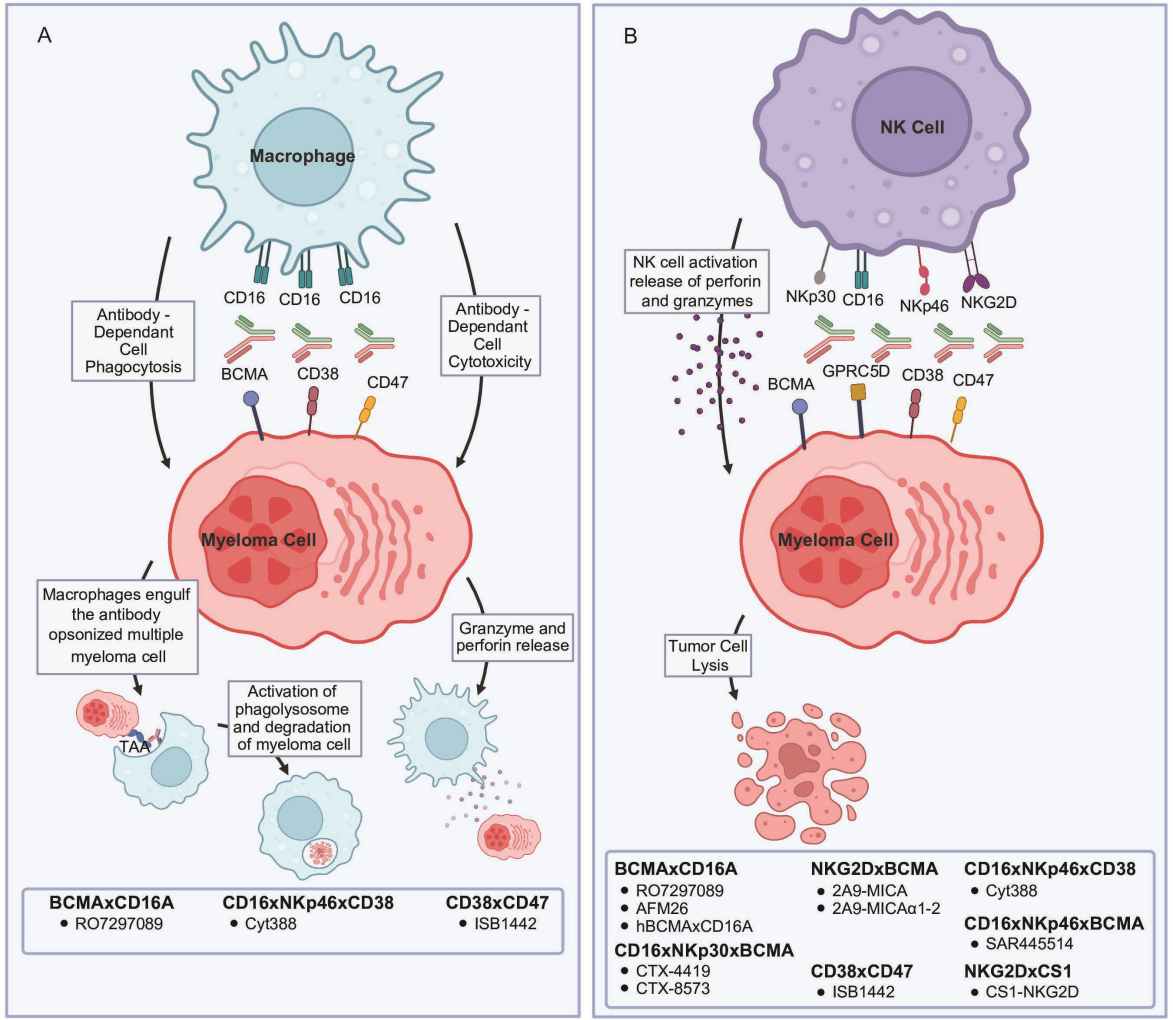
Mechanism of action of bi-specific and tri-specific NK and macrophage engagers for multiple myeloma. **(A)** M engagers bind BCMA/CD38/CD47 on myeloma cell and CD16 on M to initiate M antibody-dependent cell phagocytosis or antibody-dependent cell cytotoxicity. **(B)** Natural Killer cell engagers bind BCMA/GPRC5D/CD38 on myeloma cell and CD47/NKp30/NKp46/NKG2D/CD16 and for natural Killer antibody-dependent cell cytotoxicity through the release of perforin and granzymes, resulting in the lysis of multiple myeloma cells.

NK and M cell engagers are antibodies designed to simultaneously bind to both NK cells/M and tumor cells, creating a bridging effect that enhances the anti-tumor cytotoxic activity of these immune cells (**Figure 2**) (105). In contrast to T cells, NK and M cells offer several advantages, including reduced risk of side effects, faster immune responses, and better capacity for chemokine production, which subsequently facilitates communication with other immune cells (**Table 2**) (105–107). Therefore, emerging NKCEs and MCEs therapies could present promising alternatives for MM patients who may not benefit from TCEs due to insufficient T cell function (108, 109). These immune engagers activate NK and M cells by targeting their activating receptors, such as NKp46, NKp30, CD16A, NKG2D, and others (105). NKp46 and NKp30 are exclusively expressed in NK cells and maintain a stable expression across various cancers (106). In contrast, CD16A and NKG2D are also found in M and monocytes, and their cell surface expression is downregulated in cancer conditions (106). However, the downregulation of these costimulatory markers is reversible and does not abolish their expression and NK/M cells still retain inducible cytotoxicity (110, 111). Over the years, NKCEs and MCEs designs have successively evolved from primary bi-specific structures to more complex multi-specific formats. Notably, several recent studies support the co-engagement of different activation receptors for potent activation of NK and M cells and an enhanced anti-tumor response (10, 112). In the first section of this part, we describe recent NKCEs and MCEs currently in clinical trials (**Table 1**), and then we report the immune engagers in preclinical stages.

**Table 2:** Comparison of T, NK, and macrophages cell engagers multi-specific antibodies in multiple myeloma.

Feature	T cell engager	NK or macrophage engager
Anti-tumor mechanism	T cell induced cytotoxicity	ADCC for NK cells ADCC and ADCP for macrophages
Available Structures/multi-specific format	Bi-specific, Tri-specific	Bi-specific, Tri-specific
Safety and efficacy	- High efficacy. - High pro-inflammatory cytokines. - High incidence of CRS and neurotoxicity. - Low chemotaxis ability.	- Efficacy is still under investigation. - Less pro-inflammatory cytokine production. - Reduced risk of CRS, and neurotoxicity. - Higher chemotaxis ability (chemokines secretion and the recruitment and activation of other immune cells). - Overcome limitation of insufficient T cell function.
FDA approved drugs and Targets for MM	Telistamab (CD3xBCMA) Elranatamab (CD3xBCMA) Talquetamab (CD3XGPRC5D) Linvoseltamab (CD3xBCMA)	none
Targets in clinical/preclinical evaluation	MM cell target: BCMA, GPRC5D, CD38, CD138, SLAMF7, CD19, FCRL5, NY-ESO-1 T cell target: CD3, CD28	MM cell target: BCMA, CD38, SLAMF7 NK/macrophages cell target: CD16A, CD47, NKp46, NKp30, NKG2D

### 3.1 Clinical applications of NK and M cell-engaging bi-specific antibodies

#### 3.1.1 RO7297089

RO7297089 is a tetravalent antibody that targets BCMA on myeloma cells and CD16A expressed on innate immune cells, including NK cells, M, and monocytes (113). This antibody is designed to induce the lysis of BCMA cells through two different mechanisms: NK antibody-dependent cellular cytotoxicity (ADCC) and M antibody-dependent cellular phagocytosis (ADCP) (113, 114). Unlike conventional antibodies, RO7297089 selectively targets CD16A with no binding to Fcγ receptors of other immune cells, such as CD16B on neutrophils, resulting in a 100-fold stronger affinity than regular antibody Fc-CD16A-based interaction (114). *In vitro* assays on RO7297089 showed potent cell killing of BCMA-positive cells with varying BCMA expression levels at very low effector to target cells, with a minimal release of TNFα and IFN-γ (114). The *in vivo* anti-tumor efficacy and safety of RO7297089 were evaluated in the cynomolgus monkey model. Following five weekly intravenous administrations at 0, 15, and 50 mg/kg, monkeys showed a decrease in serum IgM and BCMA gene expression levels, a 100-fold increase and 2-fold increase in soluble BCMA and soluble CD16 levels, respectively, after the first drug administration. These findings could be explained by the systemic engagement and stabilization of BCMA and CD16A with RO7297089. In addition, the intravenous administration of the different drug concentrations was well tolerated (114, 115). In the subsequent first-in-human phase 1 study (NCT04434469), 27 RRMM patients with prior therapy received a median of 8 various doses of RO7297089 (between 60 and 1850 mg/kg). Most of the patients (19/27, 70%) experienced serious treatment-related adverse events such as thrombocytopenia, pyrexia, CRS, and others. Of the 25 response-evaluable patients, 16 (64%) had a stable disease, 4 (16%) had a minimal or partial response, and 5 (20%) had disease progression at the time of the clinical cut-off date (116). Similar to the effect observed in monkeys, soluble BCMA and soluble CD16A increased immediately after the first dose in RRMM patients (117). The apparent half-life was dose-dependent, ranging from 1.39 days to 6.52 days with the highest dose. The pharmacokinetics analysis indicates that increasing the dose could enhance the drug’s efficiency. This clinical trial has been completed without reporting any plan for a phase 2 trial. Furthermore, researchers consider that combining RO7297089 with other anti-MM therapies could have a synergistic effect.

#### 3.1.2 ISB 1442

ISB 1442 is a fully human bi-specific antibody targeting CD38 and CD47 to treat CD38-positive malignancies, including MM (118, 119). This antibody is designed with a bi-paratopic anti-CD38 arm targeting two distinct CD38 epitopes to strengthen its binding to CD38-positive tumor cells and overcome potential competition with anti-CD38 antibody therapy. The anti-CD47 arm consists of a single fragment designed to block interaction between CD47 and the signal regulatory protein alpha (SIRPα) receptor expressed on M, monocytes, and dendritic cells, enhancing antibody effector functions. Indeed, CD47 is highly expressed in MM cells (120); its binding to SIRPα allows cancer cells to inhibit or escape NK and M phagocytosis (121). Blocking of CD47 in hematological malignancies was associated with more potent anti-tumor efficacy clinically (122). ISB 1442 exerts anti-MM activity via different Fc-dependent mechanisms: ADCC, ADCP, and complement-dependent cytotoxicity (CDC) (118, 119). *In vitro*, ISB 1442 exhibited more potent tumor cell killing compared to the anti-CD38 mAb Daratumumab (Dara) alone or in combination with anti-CD47 magrolimab (52% ISB, 30% Dara, 38% Dara + magrolimab) (118). *In vivo*, ISB 1442 treatment at a dose of 3 mg/kg biweekly achieved a complete tumor regression in 9/10 preclinical mouse models, while Dara achieved the CR only in 1/10 (118). After a 60-day observation period, ISB 1442 showed a superior survival rate compared to Dara in a preclinical mouse model (100% versus 15%) (118). Generally, anti-CD47 antibodies are known to induce red blood cell (RBC) hemagglutination due to their on-target off-tumor depletion of RBCs (123). Importantly, ISB 1442 demonstrated a favorable safety profile with a low potential for adverse events such as hemagglutination, platelet aggregation, and RBC depletion (118). A subsequent phase 1/2 clinical trial is ongoing to assess the safety, tolerability, and efficacy of ISB 1442 in 121 RRMM patients (NCT05427812). Preliminary data from this ongoing trial, including 10 RRMM patients with prior anti-myeloma line therapy receiving once weekly ISB 1442 in 4 dose-escalation groups from 6 mg to 150 mg, showed that the drug was well tolerated (124). Most of the observed treatment-related adverse events (TRAEs) were moderate (grade 1 or 2), with the absence of grade 5 TRAE (124). The same study revealed that several patients had a transient increase in M-related inflammatory proteins within 24h after ISB 1442 treatment, which is associated with M activation (124). Although ISB 1442 showed promising results in the early-stage trial, other measures, such as drug efficacy, pharmacokinetics, and immunogenicity, will be provided upon completion of the trial.

### 3.2 Clinical applications of NK and M cell-engaging tri-specific antibodies

SAR445514 is an anti-NKp46xCD16xBCMA tri-specific antibody designed to efficiently activate NK cells and redirect them to engage and kill BCMA-expressing cells. The dual engagement of both NKp46 and CD16A induced a stronger NK activation compared to the single engagement of these two molecules (125). This construct demonstrated a significant NK cell-mediated cytotoxicity against BCMA-expressing MM cells *in vitro* with an incredibly low cytokine release. *In vivo* evaluation reported a higher OS of 90% and a median survival day over 90 days in the mouse model treated at different doses of SAR445514 (from 0.5 mg/kg to 5 mg/kg) (125). More recently, Tang et al. confirmed that SAR445514 has potent anti-tumor activity against tumor cells resistant to standard therapies while inducing minimal cytokine release (126). Consequently, this antibody became the first tri-specific NK-cell engager to enter a phase 1/2 clinical trial in May 2023 for the treatment of RRMM and relapsed/refractory light chain amyloidosis (RRLCA) (NCT05839626), but the trial is currently terminated based on the sponsor’s decision.

### 3.3 Preclinical applications of NK and M cell-engaging multi-specific antibodies

#### 3.3.1 NK and M cell-engaging multi-specific antibodies targeting BCMA

##### 3.3.1.1 AFM 26 and hBCMAxCD16A

AFM 26 is a novel tetravalent bi-specific antibody designed to induce a potent NK cell anti-MM cytotoxicity through a high-affinity bivalent binding to CD16A and a diversion of NK cells to engaged BCMA-expressing cells (127, 128). AFM 26 induces a strong anti-tumor NK-specific activity compared to the classical antibody format *in vitro*, independently of CD16A polymorphism (129). This construct is differentiated from other antibodies by a prolonged cell retention time, unaffected by high levels of circulating IgG (129). These data presented AFM26 as a promising treatment for MM therapy alone or in combination with other complementary immunotherapy agents.

Recent studies showed that the antibody size plays a crucial role in determining its binding properties, affinity, immunogenicity, and stability (130). In recent years, there has been a growing interest in engineering smaller antibodies to improve their tumoral uptake, distribution, and tumor lysis efficacy (131, 132). Such a strategy could be of great interest in MM treatment, allowing the penetration of tumor cell reservoirs in the bone marrow and lesions outside the bone marrow, which implies a poor prognosis. Accordingly, Giang et al. recently developed a small format of anti-BCMAxCD16 NK engager with a smaller molecular weight of 23 kDa, compared to AFM 26 (105 kDa), to increase its tumor penetration, efficacy, and stability (133). This study showed that hBCMAxCD16a dual engagers, affibody, efficiently activated resting NK cells and induced a potent lysis of human BCMA-positive MM cells *in vitro* (133). On the other hand, it has been hypothesized that immune engagers with a small molecular size could have improved tissue penetration, including EMD and blood-brain barrier penetration compared to full- length antibodies, but clinical evidence is limited. A recent case report has demonstrated that a 62-year-old female with high-risk MM who developed extensive leptomeningeal myelomatosis showed an improvement in leptomeningeal disease following two cycles of teclistamab. This study supports the possible biological penetration of this TCE and its activity in the central nervous system (134, 135). However, further preclinical studies are needed to evaluate the safety, pharmacokinetics, and efficacy of this uniquely small NK engager.

##### 3.3.1.2 CTX-4419 and CTX-8573

CTX-4419 and CTX-8573 are novel bi-specific antibodies targeting NK cells by co-engagement of NKp30 and CD16A and myeloma cells through BCMA (136, 137). The main advantage of these constructs is that their anti-tumor function does not require the CD16A engagement by the Fc region; Nkp30 alone is sufficient to promote this activity (136, 137). Notably, this feature could overcome the reduction or loss of CD16A activity due to FC receptor shedding or downregulation in the tumor microenvironment (136, 137). *In vitro* studies demonstrated that both NK engagers elicit a potent anti-MM activity against BCMA-expressing cells, estimated to be 100-fold greater than anti-BCMA mAb for CTX-8573. CTX-8573 also has an additional benefit of activating γδT cells in addition to NK cells, which is not seen in CTX-4419 (136, 137). Moreover, both NK engagers showed efficacy in pre-clinical models, which correlated with a reduction of plasma cell count and with a good safety profile. Importantly, the two drugs maintain their cytotoxicity effect in the presence of BCMA and BCMA ligands and serum IgG without the induction of NK-cell fratricide (136, 137). However, the main concern of NKp30 targeting therapies is their low abundance (approximately 1000 molecules) on the NK cell surface compared to other targets such as CD16A (around 70,000 molecules per NK cell) (138). Thus, the development of anti-NKp30 NK engagers with a more flexible structure able to bind distant targets could overcome this limitation.

##### 3.3.1.3 MHC class I-related chain A domains: 2A9-MICA and 2A9-MICAα1-2

MHC class I-related chain A (MICA) expressed on myeloma cells is a ligand of NKG2D. The interaction between MICA and NKG2D triggers the activation of NK cells and the co-stimulation of T cells (139). However, cancer cells can evade the anti-tumor immune response through MICA shedding (140). Recently, Wang et al. designed a novel NK engager targeting NKG2D and BCMA to restore the NKG2D-mediated surveillance and enhance the immune response in MM patients (141). *In vivo* and *in vitro* studies reported that 2A9-MICA effectively activated NK cells, induced cytolysis of MM cells, and inhibited tumor growth (141). Further advancements led to the development of a new format of 2A9-MICA, aiming to improve the affinity and specificity of the antibody (142). The novel construct is characterized by the dual engagement of two NKG2D domains and BCMA on cancer cells. Compared with 2A9-MICA, 2A9-MICAα1–2 had a stronger affinity to BCMA and NKG2D, which mediated the cytotoxicity of NK effector cells against MM cells (142). *In vivo*, 2A9-MICAα1–2 showed superior anti-MM efficacy, tumor growth suppression, activation of CD56^+^ TNF-α^+^ NK cells, and immune infiltration within the tumor microenvironment (142).

#### 3.3.2 NK and M cell-engaging multi-specific antibodies CD38

CYT-338 is a tetravalent bi-specific antibody that targets NKp46 and CD16 on NK cells and CD38 on myeloma cells. This construct is designed to overcome the anti-CD38 mAb, Dara, mediated fratricide of NK cells by engaging the NKp46 activation receptor (143). Interestingly, preclinical investigations reported that the engagement of NKp46 via CYT 388 enhanced the NK cell cytotoxicity while preventing cell exhaustion (143, 144). Indeed, *in vitro* studies demonstrated that CYT-338 mediates anti-tumor activity through ADCC, ADCP, and CCD mechanisms involving NK cells, M, and complement pathway activation (144). Importantly, CYT-338 showed higher cytotoxicity and enrichment of lymphocyte and leukocyte activation receptor-related genes in MM patients’ tissues compared to Dara (144). These findings encouraged researchers to develop a first-in-human phase 1 study targeting RRMM patients in the United States (143). NAYA Therapeutics announced that patient enrolment for CYT-338 (NY-338) Phase I/II clinical trials is expected in early 2026 (145).

#### 3.3.3 NK cell-engaging multi-specific antibodies targeting SLAMF7

CS1-NKG2D is a bi-specific NK-engaging antibody that targets CS1 (alternative gene name for SLAMF7), a tumor antigen expressed on MM, and the NK activation receptor NKG2D. Compared to the previously mentioned NK-engaging bi-specific antibody, this construct is considered less effective. Indeed, CS1-NKG2D can trigger *in vitro* NK cytotoxicity only in MM expressing high levels of CS1 and at high effector-to-target ratios. Similarly, the survival benefit reported in the CS1-NKG2D-treated mice model was dependent on the level of MM cells’ CS1 expression. Mice engrafted with CS1-high MM cells showed a significant survival extension, while those with CS1-intermediate MM cells experienced only a modest survival benefit (146).

## 4 Challenges and limitations of T cells, NK cells, and macrophages engaging multi-specific antibodies

**Figure 3:**
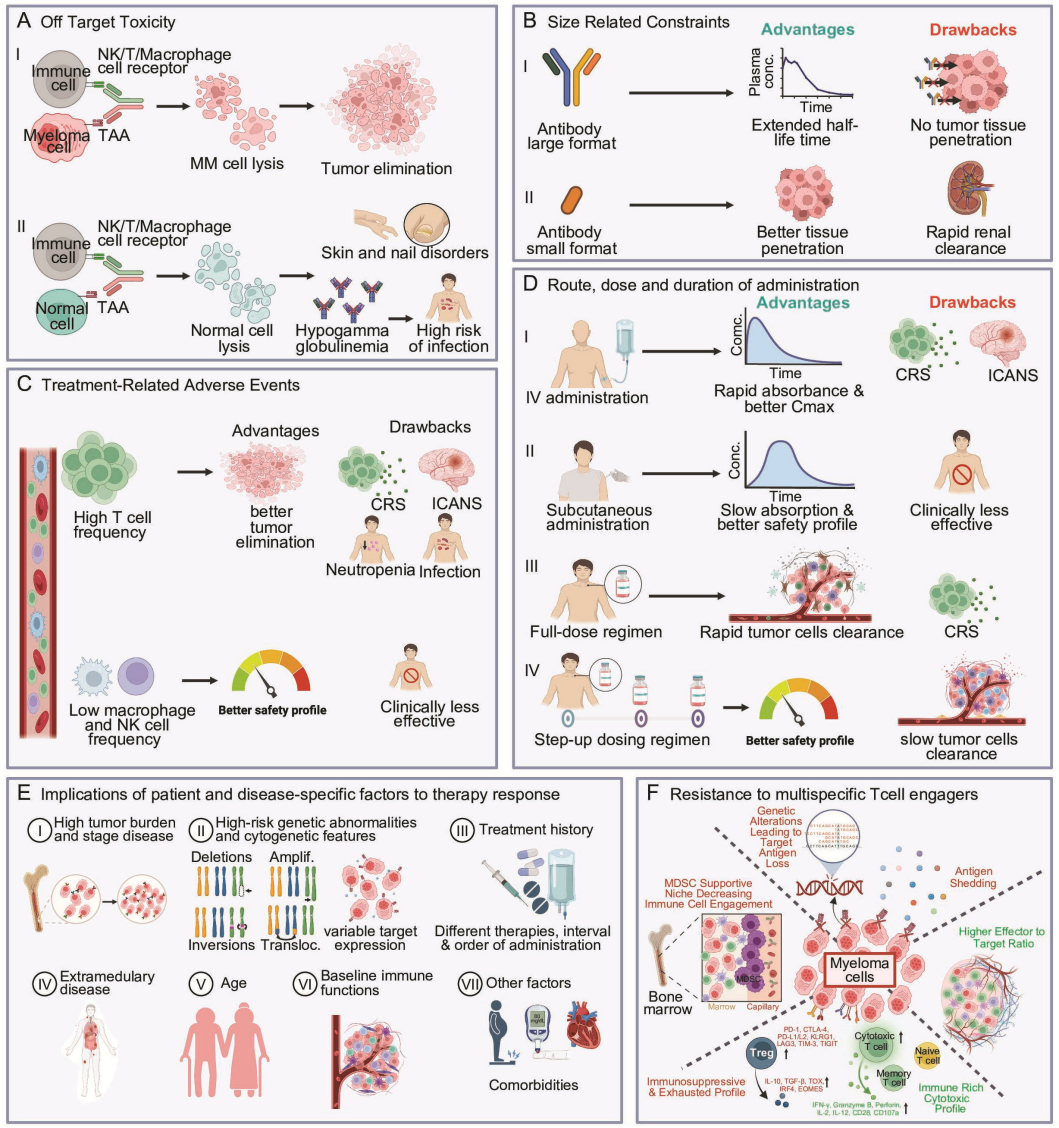
Challenges and limitations of the multi-specific antibodies. **(A)** Tumor-associated antigens are usually expressed on both malignant and healthy cells at varying expression levels. Though ideally, (I) the antigen expression on malignant cells will lead to tumor elimination, (II) their expression on normal cells leads to off-target toxicity such as hypogammaglobulinemia or skin and nail disorders. **(B)** (I) Larger constructs exhibit prolonged half-lives but limited tumor penetration, whereas (II) smaller antibody formats enable improved tissue penetration but undergo accelerated rapid clearance. **(C)** Due to the high frequency of T cells in the blood, TCE shows an efficient treatment response but are frequently associated with adverse events such as CRS, ICANS, neutropenia, and infections. In contrast, NK cell and Mengaging MsAbs have a lower risk of treatment-related toxicities that can be attributed to their relatively lower blood frequency. **(D)** (I) Intravenous (IV) administration enables rapid drug absorption, reaching peak serum concentration quickly, which facilitates swift tumor cell clearance, but this exposure leads to a higher incidence of toxicity. (II) Subcutaneous (SC) administration results in slower absorption, causing less immediate antitumor activity; however, SC delivery is also linked to a reduced risk of toxicities. (III) Patients receiving a single full-dose regimen experienced rapid tumor elimination, but experienced higher CRS incidence, while (IV) a step-up dosing schedule provided improved safety with slower antitumor efficacy. **(E)** Several patient- and disease-specific factors may influence treatment response, as shown in I) Patients with high tumor burden or advanced-stage disease often exhibit genetic heterogeneity and elevated antigen levels, resulting in initial responses to MsAbs followed by relapses due to immune exhaustion. II) High-risk genetic and cytogenetic abnormalities contribute to antigen heterogeneity, drive rapid adaptation, and thus limit response durability. III) Prior exposure to targeted therapies may reduce response to subsequent agents targeting the same antigen, due to antigen downregulation and clonal expansion of low- or negative-expressing tumor cells. The interval and order of administered treatments also influence treatment efficacy and its persistence. IV) MsAbs encounter reduced antigen availability and limited immune cell infiltration in extramedullary sites, thus restricting therapeutic access and efficacy. V) Aging-related immune decline contributes to impaired T cell activation and degranulation, sustains an immunosuppressive microenvironment, and limits MsAb efficacy. VI) Baseline immune fitness is associated with improved responses, while prior treatment exposure correlates with a more exhausted immune profile and reduced tumor clearance. VII) Comorbidities and obesity could influence the efficacy outcomes of MsAbs. **(F)** Excessive antigen shedding diminishes surface target availability, contributing to immune escape and treatment resistance. Antigen loss may also result from genetic alterations, epigenetic silencing, or alternative splicing of antigen-encoding genes. High T cell frequencies and favorable effector-to-target ratios were consistently associated with improved responses to bi- and tri-specific T cell engagers, while low T cell numbers correlated with primary resistance. TCE efficacy is linked with a cytotoxic and memory T cell-activated profile; in contrast, T cell exhaustion and immunosuppressive markers accelerate disease progression. Myeloid-derived suppressor cells (MDSCs) promote immune resistance in MM by shielding tumor cells, limiting TCE access, and activating survival pathways.

T, NK cells, and Mengaging MsAbs have demonstrated promising results in treating various hematological malignancies, including MM (10, 105, 106, 147). However, these immune-engaging therapies confront multiple challenges (**Figure 3**). Addressing these challenges should pave the way for developing novel and innovative strategies to enhance these antibodies’ efficacy, stability, and safety profile.

### 4.1 Off-target toxicity

The first challenge in immune cell engager MsAbs is the selection of an appropriate antigen with predominant expression limited to tumor cells rather than normal cells to prevent off-target effects. However, most tumor-associated antigens (TAAs) do not fulfill this requirement since most of them are usually expressed on healthy and tumor cells, but with different expression levels (148). Indeed, patients treated with MsAbs targeting BCMA, GPRC5D, and FcRH5 experienced hypogammaglobulinemia, which may increase infections risk due to the expression of these antigens in normal plasma cells (15). Additionally, GPRC5D is also expressed in keratin-producing cells, which could result in skin and nail disorders observed in patients treated with GPRC5D-targeting MsAbs (149–151). Consequently, the development of immune engagers targeting multiple TAAs, such as tri and tetra-specific antibodies, could be an effective strategy to enhance the specificity and affinity of these antibodies against tumor cells and minimize the off-target related adverse events. Indeed, the simultaneous recognition of two or more tumor antigens by tri- or tetra- MsAbs will restrict the activation of immune cells to tumor cells that co-express these antigens while reducing damage to normal cells expressing a single antigen, thereby improving safety (10, 152, 153).

The tumor microenvironment is characterized by an acidic pH due to the high lactic acid production by tumor cells during glycolysis (154). Interestingly, researchers designed antibodies that are activated only in the acidic conditions of the tumor microenvironment, while their activity is diminished in the physiological alkaline environment of normal tissues to minimize off-target toxicity (155).

Interestingly, the development of oncolytic viruses armed with a therapeutic transgene encoding Bi-specific NK and T cell engagers may be an appealing platform for delivering bi-specific antibodies (156). This strategy will target and induce viral replication only in tumor cells while sparing normal cells (156). Two oncolytic-based T cell engagers targeting epidermal growth factor receptor (EGFR) and Epithelial cell adhesion molecule (EpCAM) were previously evaluated *in vivo* and *in vitro* for cancer therapy (156). However, there are no relevant engineered MsAbs-producing oncolytic viruses currently undergoing preclinical evaluation for MM therapy.

Another crucial aspect in the development of immune-engaging MsAbs is their affinity for TAAs or immune-stimulatory markers such as CD3. The development of antibodies with extremely high binding affinity could enhance anti-tumor immune cells cytotoxicity but increase the risk of systemic T cell activation, CRS, and off-tumor toxicity. Conversely, antibodies with lower affinities could improve tumor selectivity, specificity, and safety but reduce efficiency. Therefore, effort is needed to identify the optimal antibody affinity that is sufficiently strong to bind to TAAs or immune stimulatory markers while minimizing the off-target toxicity (157).

### 4.2 Size-related constraints

The molecular structure of immune-engaging MsAbs plays a crucial role in determining their efficacy and pharmacokinetics. Smaller formats may provide better tissue penetration but are susceptible to rapid renal clearance. In contrast, larger structures tend to have extended half-life times but may not penetrate the tumor tissue. In recent years, researchers have become increasingly interested in nanobodies. Nanobodies are single-domain immunoglobulin structures derived from naturally occurring heavy-chain antibodies found in camels (158). Studies have demonstrated that nanobody-based MsAbs are smaller and more soluble, offering enhanced stability and improved tumor penetration *in vivo* (159). Although nanobody-based MsAbs immune engagers offer significant advantages, their small size, falling below the kidney filtration threshold, results in a shorter serum half-life *in vivo*. To address this, researchers have developed several strategies for half-life extension over the last decades including fusion with the Fc domain of human immunoglobulin G (IgG), polyethylene gylation, and fusion to human serum albumin (HSA) or albumin-binding moieties, which have exhibited clinical promise in prolonging the systemic retention of the antibodies (160). Interestingly, one study on MM cells reported that CD38-specific-extended half-life nanobody-based MsAbs targeting CD38 on MM cells and CD16 on NK cells induced superior cytotoxicity against CD38-positive myeloma cell lines than the conventional daratumumab (161). These nanobodies bind to three distinct and non-overlapping epitopes of CD38 on MM cells, activate NK cells by targeting CD16 through central nanobodies, and bind to albumin via a C-terminal nanobody. As a result, the trimer not only targets the antigen and activates NK cells but also extends the construct’s half-life (161). In addition, Ding et al. designed a novel nanobody-based tri-specific T cell engager targeting fibroblast activation protein (FAP), programmed cell death protein 1 (PD-1), and CD3 to overcome tumor-mediated immunosuppression. *In vitro*, this construct showed a strong affinity with its targets and potent antigen-specific cytotoxicity. *In vivo*, this nanobody-based tri-specific antibody suppressed tumor growth, enhanced T cell infiltration, and improved survival rate in mouse models compared to the nanobody form (162). The efficacy of other NK and M engaging nanobody-based MsAbs is being tested *in vitro* for MM therapy (161–163). Altogether, these novel bi-specific/tri-specific immune engager nanobodies offer a promising therapeutic platform with enhanced therapeutic effects in MM with a good safety profile.

### 4.3 Treatment-related adverse events

T cell-engaging MsAbs have revolutionized therapies for MM and other hematological malignancies. However, these constructs were also associated with multiple adverse events such as CRS, ICANS, neutropenia, infections, as well as others. CRS is a systemic inflammatory syndrome caused by a massive release of pro-inflammatory cytokines such as IL-6, IL-1β, and TNF, α due to the activation and expansion of T cells (164). ICANS is associated with proinflammatory cytokines in the cerebrospinal fluid and elevated serum angiopoietin-2 (ANG2) (164). In the phase I/II trial (NCT03145181, NCT04557098), treatment with teclistamab resulted in grade 1 or 2 CRS in 72.1% of patients (18). Hypogammaglobulinemia and an increased risk of infection can occur due to BsAbs targeting healthy plasma cells or B cells (165).

Several strategies are currently recommended for the prevention and management of these adverse events (166). Importantly, patient examination and regular laboratory data monitoring are essential for the detection of changes and onset of symptoms during treatment as early as possible (167). CRS generally occurs 0–16h after T cell-engaging MsAbs infusion (167). Therefore, it is mandatory to monitor patients regularly, ideally on an hourly basis (167). For patients experiencing grade 2 CRS, corticosteroids are recommended. Premedication with corticosteroids may prevent CRS in patients who developed grade 2 CRS during a previous cycle of T cell-engaging MsAbs. Anti-IL-6 drugs might be considered for patients during grade 1 CRS monitoring and escalation for grade ≥2 CRS (166–168). ICANS, often alongside CRS, glucocorticoids are recommended, if necessary, along with anakinra if the response is insufficient, and anticonvulsants if convulsions occur (166). Preventive measures against infections include antiviral and antibacterial medications as well as the administration of immunoglobulins (166). Other strategies are also being tested for CRS management, including blockade of IL-1β, TNF-α, IFN-γ, and GMCSF and the use of kinase inhibitors (169).

NK and M engagers showed a more promising safety profile than T cell engagers due to a lower risk of adverse event development (170). These outcomes could be explained by distinct reasons. First, the blood frequency of NK and M is six to eight times lower than T cells, resulting in a moderate release of inflammatory cytokines upon treatment, thus limiting the risk of CRS in treated MM patients (171). The cytokine release induced by NK and M engaging remains within the physiological ranges (106). Second, the spectrum of cytokines and chemokines secreted by NK and M cells is distinct from that secreted by T cells, linked to the onset of these adverse events (172, 173). Preclinical studies with NK engagers have demonstrated a specific killing activity and a safer toxicity profile compared to T cell engagers (174). However, clinical studies showed that a large proportion of cancer patients did not benefit from this treatment (174). Currently, several groups are testing different strategies to promote the efficacy of NK and M engagers in MM, such as the generation of NK and M-based nanobodies with an extended half-life to enhance the antibody stability and its therapeutic effect (161–163). Moreover, the co-engagement of multiple activating receptors of NK cells could induce a more potent anti-tumor activity (125, 136, 137). Another strategy is the addition of a cytokine-based crosslinker, such as IL-15, promoting persistent activation and proliferation of NK and M cells (175). Furthermore, the development of multi-specific antibodies that block checkpoint receptors such as PD-1, KIR, NKG2A, or TIGIT involved in NK and M suppression could overcome tumor cell immune escape (162). Finally, the combination of NK engagers with other anti-MM therapies could have a synergistic cytotoxic effect, leading to accelerated tumor cell degradation (106).

### 4.4 Route, dose, and duration of administration

Recently, several studies showed that the route of administration could impact the MsAbs’ pharmacokinetics and toxicity profile (176, 177). Indeed, intravenous (IV) administration provides a rapid absorption of the drug linked to an immediate maximum serum concentration (Cmax) within 0.5 to 4 hours, thus a rapid clearance of tumor cells (178). However, rough exposure to high doses of the drug in IV administration was also associated with a higher incidence of CRS and neurotoxicity (176). In contrast, the subcutaneous (SC) administration induced a slow absorption of the drug (a Cmax reached between 0.7 to 2 days), leading to less efficient anti-tumor killing (178). Notably, this route of administration showed a lower incidence of CRS and neurotoxicity due to the gradual sensitization and priming of the immune cells to the drug (176).

The dose could also impact the MsAbs’ pharmacokinetics and toxicity profile. Higher CRS events were observed in patients treated with a first full dose regimen compared to those treated with a two-step up-dosing regimen. Importantly, larger constructs may benefit from IV administration due to their longer circulation time, while SC administration of smaller constructs enables a gradual absorption of the drug, potentially extending their effective duration (106).

Another critical factor to consider is the treatment duration. To our knowledge, most MsAbs immune engagers in clinical trials were administered for fixed-duration treatment. The option of continuous administration of low doses of the drug until disease progression or the onset of intolerable toxicity could also be considered. However, this approach could increase the treatment costs and the need for long-term healthcare utilization by patients.

### 4.5 Implications of patient and disease-specific factors to therapy response

There are several patient and disease- specific factors that could affect treatment response in MM. For example, patients with a higher tumor burden and late-stage disease often have a variable genetic profile with elevated levels of circulating antigens, leading them to initially respond to immune-engaging antibodies but relapse as tumor cell populations evolve and eventually lead to immune exhaustion (179, 180). High-risk genetic abnormalities in MM, such as deletions of chromosome 17p (del(17p)), translocation t(4;14), gain of chromosome 1q (1q^+^), and translocation t(14;16), are associated with poor ORR to therapies, including MsAbs. High-risk cytogenetic features present diverse populations of myeloma cells with varying target antigen expression. As a result, immune-engaging therapies will effectively target only some subsets of cells within the heterogeneous population. Moreover, the underlying genetic instability often leads to cancer cells’ resistance, thus limiting the durability of response (45, 68).

Given that aging is associated with a decline in immune function, talquetamab activity was reduced in samples from older patients (over 67), along with decreased T cell activation and degranulation (15). These features inherently sustain an immunosuppressive microenvironment, leading to an inadequate response to MsAbs in MM.

Extramedullary disease (EMD) occurs with the spread of myeloma cells outside the bone marrow, involving organs, soft tissues, or other locations, and is associated with more aggressive disease behavior (181). MsAbs face barriers of reduced antigen availability in extramedullary sites and limited access to immune cells due to the challenge of infiltrating these sites (45, 68, 182). In addition to BCMA targeting agents (17), patients with extramedullary plasmacytomas also faced lower response rates (40-45%) to anti-GPRC5D BsAb (151).

Previous treatments often influence the biology of MM cells and the immune environment, thus impacting the effectiveness of subsequent immunotherapies. Patients previously treated with targeted therapies may experience a reduced response to subsequent agents, specifically those targeting the same antigen, due to antigen downregulation from prior selective pressures. These therapies may lead to clonal selection, where clones of negative or low-expression target antigens expand over time. Indeed, both elranatamab and teclistamab showed a lower ORR, 53.8% and 50% respectively, in patients who received prior BCMA treatment (19, 68). In contrast, talquetamab induced high responses (67%-83%) in RRMM patients with prior exposure to BCMA-CART, suggesting GPRC5D targeting therapy may overcome resistance mechanisms associated with previous BCMA-directed therapies (37). Patients who are treatment-naïve or have not received extensive prior treatments often show better responses to MsAbs, as they likely have preserved antigen expression and a less resistant tumor cell population (45). In the MonumenTAL-1 study, patients without prior T cell redirection therapy displayed a more favorable immune profile, with higher T cell counts and fewer immune-suppressive features, such as Tregs and inhibitory checkpoint markers (such as lymphocyte-activation gene 3 (LAG-3), T cell immunoglobulin and mucin-domain containing-3 (TIM-3), and PD-1) on CD8^+^ T cells. This baseline immune fitness improved responses to talquetamab, while patients with prior exposure exhibited a more exhausted and immunosuppressive profile with an impaired response (183).

The ORR also varies based on the interval between prior T cell redirection therapies and the initiation of talquetamab. A longer interval (>9 months) between prior BsAb treatment and talquetamab was associated with a higher response rate (78). Furthermore, switching different T cell directed therapies after relapse can maintain response durability, achieving an ORR rate of up to 80% and extended survival, even in heavily pretreated patients (184). A recent study reported that sequencing CAR-T therapy followed by BsAbs may provide optimal outcomes, with higher response rates and longer duration of response, specifically when alternating antigen targets (from BCMA to GPRC5D) (185). On the other hand, immunomodulatory drugs, proteasome inhibitors, and anti-CD38 monoclonal antibodies enhance immune activation and mediate potential synergistic effects with T- and NK-cell engagers (15, 186). Thus, treatment history serves as a critical factor for stratifying patients and guiding subsequent adaptive therapy adjustments.

## 5 Mechanisms of resistance and predictive biomarkers of response to multi-specific antibodies

Despite the promising therapeutic activity of multi-specific immune cell engagers in MM, available clinical data remains limited. The available reported patient outcomes appear heterogeneous, ranging from deep responses in some patients to resistance, relapse, or modest impact in others. This variability underscores the need to identify biomarkers for response and resistance, benefiting both physicians and patients with cost-effective and efficient treatment outcomes. Biomarker-guided dosing strategies can help optimize therapeutic windows by adjusting treatment doses and duration based on immune and tumor-related biomarkers. Real-time monitoring of patient responses also facilitates timely therapeutic interventions, such as switching therapies or devising synergistic treatments to avoid unnecessary toxicity to the patients (187). These predictive biomarkers can be categorized into tumor-intrinsic factors, which refer to the molecular, genetic, or phenotypic aspects inherent to the malignant plasma cells, and tumor-extrinsic factors, which encompass elements of the tumor microenvironment, the immune system, and the patient characteristics (188).

### 5.1 Tumor-intrinsic factors

A high tumor burden was also associated with lower response rates to MsAbs (17, 45). Tumor antigen loss is a well-documented mechanism through which cancer cells evade immune recognition, particularly in the context of targeted immunotherapies. As discussed earlier, MsAbs rely on target antigens such as BCMA, GPRC5D, CD38, CS1, and FCRL5 to recognize and bind myeloma cells. Preclinical studies demonstrated that the density and expression levels of these TAAs were positively correlated with a potent anti-tumor efficacy by the immune-engaging MsAbs (14, 118, 146, 180, 189–191). However, tumor cells can escape immune surveillance of the immune engagers through the downregulation or expression loss of the TAAs (192). Therefore, stable and high antigen expression can serve as a biomarker of response to immune-engaging MsAbs, whereas antigen loss contributes to treatment resistance.

Target antigens such as BCMA can be cleaved from the cell surface and shed into the serum via secretase, forming soluble BCMA (62). Increased levels of sBCMA in the blood were linked with disease burden in MM and poor outcomes (31, 180, 193, 194). sBCMA can be measured non-invasively at regular intervals and could be used for monitoring disease dynamics during treatment. Upon treatment, responding patients showed a reduction in sBCMA, while progressive patients sustained increased sBCMA levels (17, 68, 95, 117, 193, 195). In this line, persistently elevated sBCMA may indicate ongoing disease burden or the emergence of treatment resistance mechanisms (193, 196). Importantly, a Phase I study evaluating RO7297089 (anti-CD16xBCMA NK/M engaging antibody) found that baseline NK/M counts did not correlate directly with treatment outcomes, but elevated sBCMA and CD16A post-treatment infusion did associate with stabilized target engagement and response (117). However, changes in sBCMA should be interpreted cautiously, as reduced levels may reflect not only decreased tumor burden but, in some cases, altered BCMA expression or antigen escape, thus diminishing its reliability as a definite marker of treatment efficacy (197, 198). The presence of soluble target antigens may also act as a “soluble sink,” reducing the engagement of RO7297089 to cell-bound BCMA, potentially diminishing its efficacy (116). Decreased target antigen levels due to excessive shedding clearly reduce their availability on the cell surface, thus manifesting an escape mechanism by the tumor cells and therapy resistance (179). To overcome this resistance mechanism, some studies have investigated adding a secretase inhibitor. In a preclinical model, incorporating γ-secretase inhibitor resulted in enhanced BsAbs efficacy (14, 199). More specifically, teclistamab activity was enhanced when the γ-secretase inhibitor, LY-411575, was added to MM cells (14). Similarly, an increased potency in HPN217 was reported when treated in combination with a γ-secretase inhibitor (200).

Mechanisms of antigen loss can also be attributed to genetic alterations, epigenetic silencing, or alternative splicing of antigen-encoding genes. For example, mutations or epigenetic modifications in the target gene can cause reduced or loss of expression on the surface of myeloma cells. Decreased target antigen levels due to excessive shedding may reduce availability on the cell surface, thereby manifesting immune escape and therapy resistance (179). Multiple studies have investigated tumor-intrinsic resistance mechanisms to talquetamab. One report identified convergent evolution in a patient with a clonal 12p deletion in the pre-treatment sample. Interestingly, seven resistant subclones emerged at relapse, each acquiring distinct mutations that resulted in the complete loss of GPRC5D protein on the cell surface (179, 201).

Truger et al. conducted whole genome sequencing and RNA-sequencing on samples from 100 MM patients, half newly diagnosed, and the others RRMM, to further identify pre-existing vulnerabilities and potential resistance mechanisms before and after T cell-engaging antibodies therapy. The research group found that about 30% of immunotherapy-naïve MM patients already had heterozygous deletions in key immunotherapy target genes, such as GPRC5D, CD38, SDC1, TNFRSF17, and NCAM1. This did not reduce gene expression directly but may predispose cells to antigen loss after therapy. The RRMM patients exhibited more complex karyotypes, with increased deletions, suggesting clonal evolution and resistance (202).

In addition, Lee et al. revealed mechanisms of antigen escape in BCMA- and GPRC5D-targeted immunotherapies using bulk and single-cell whole-genome sequencing and copy number variation (CNV) analyses in MM (179). Among 14 patients who progressed on anti-BCMA T-cell engager therapy, 42.8% had TNFRSF17 alterations, including biallelic loss in one patient and extracellular-domain mutations in five patients. In one patient with triple-class refractory MM who relapsed 6 months after anti-BCMA T-cell engager therapy, 99.5% of cells at relapse showed biallelic TNFRSF17 loss with absent BCMA expression. Likewise, in a patient treated with talquetamab, single-cell CNV analysis showed monoallelic GPRC5D loss in 79% of cells and biallelic loss in 0.2% before treatment, while at relapse, 93.6% of cells showed clonal biallelic focal deletion of GPRC5D (201). Notably, a recent study by Papadimitriou et al. identified that the emergence of antigen loss is primarily acquired during treatment and emphasizes the need for ongoing monitoring rather than static baseline screening to detect these resistance mechanisms early (203, 236). Several tri-specific immune engagers have entered clinical trials lately and are currently undergoing early-stage clinical trials for MM therapy (10, 105, 106, 147).

### 5.2 Tumor-extrinsic factors

The tumor microenvironment (TME) of MM, specifically in the immunological components, plays a crucial role in determining both response and resistance to MsAbs. The interactions within the TME can either facilitate effective immune cell engagement and subsequent tumor cell destruction or create barriers that lead to therapeutic resistance. An adequate, competent immune status with stable immune effector cell activation remains the prerequisite for an efficient anti-tumor response (190, 204).

#### 5.2.1 T cell frequency

Several studies have provided evidence for a clear relationship between T cell frequencies and the efficacy of bi-specific and tri-specific TCE in treating MM patients. For example, preclinical killing assays with bone marrow samples from MM naïve patients showed that teclistamab and talquetamab anti-MM activity strongly correlated with a high T cell/MM cell ratio (8). Correlative analyses from the MajesTEC-1 study demonstrated that a higher proportion of peripheral T cells was crucial for an effective response to teclistamab, whereas, lower T cell numbers was associated with resistance in non-responders (31). The efficacy of talquetamab was dependent on a higher effector-to-target ratio in bone marrow samples (15). Similarly, high T cell frequencies were associated with improved JNJ-7957, an anti-BCMA mediated anti-MM activity (158). A combined analysis of resistance mechanisms to both teclistamab and talquetamab demonstrated a low ratio of T cells to MM cells associated with primary resistance (205). Correspondingly, an ex vivo biomarker study of tri-specific TCE, SAR442257, indicated a low effector-to-tumor cell ratio as a resistance marker (104). Another tri-specific TCE, ISB 2001, co-targeting BCMA and CD38 on MM cells, evidenced high infiltration of T cells, along with cytokine release in the tumor site of responders (206).

#### 5.2.2 T cell functional and phenotypical characteristics

The functional and phenotypic characteristics of T cells are key determinants of clinical outcomes in MM therapies involving TCE. Preclinical assessments endorsed an immune-rich environment and an activated cytotoxic profile with a surge in CD8^+^, naïve, and memory IFN-γ, granzyme B- secreting T cells for a consistent therapeutic response to bi-specific anti-BCMA therapies. An exhausted and immunosuppressive environment, reserving Tregs along with PD-1, PD-L1, LAG3, KLRG1, and excessive IRF4 markers, effectuated progression (180, 186). Patients who respond to teclistamab have shown early immune activation with increased cytokines IFN-γ, IL-6, IL-10, and IL-2 receptor α. Elevated levels of CD38 and TIM-3 on CD8^+^ T cells of these patients also depicted immune readiness early in treatment (17). The MajesTEC-1 trial proposed immune fitness, constituting a higher cytotoxic CD8^+^ T cell population expressing granzyme B and perforin for an efficient response to teclistamab. Contrarily, higher baseline frequencies of Tregs/CD38^+^ Tregs and exhausted T cells expressing markers such as PD-1, TIM-3, and CD38 represented tumor burden and resistance in non-responders. Increased PD-1^+^ CD8 T cells and Tregs in the periphery, along with CD25^+^ and CD38^+^ CD4 T cells in the bone marrow, were associated with shorter PFS (31). Baseline PD-1 expression was significantly associated with the ORR in the trial (20). An analysis of progression and relapse immune profiles in another phase 1/2 MajesTEC-1 teclistamab study noted lower CD28 expression on CD8^+^ T cells and higher expression of exhaustion markers (CD38, PD-1, TIM-3, EOMES, TOX) and perforin on both CD4^+^ and CD8^+^ T cells. Further, significantly increased CD38 expression on T cell receptor gamma delta T cells and higher PD-1^+^/TIGIT^+^ Tregs were detected at progression in comparison to baseline (183). The dynamics of the pre-existing T cell landscape in MM patients receiving bi-specific TCEs, specifically BCMAxCD3 antibodies, were investigated. The authors reported conserved patterns of activity in bone marrow-residing T cells. They transcriptionally defined subsets that expanded in responders who showed clonal expansion of CD8^+^ T cells with minimal signs of exhaustion, especially in the CX3CR1^+^ effector subset, known for strong cytotoxic activity. A potential reserve of naïve T cells with active MHC class I signaling was crucial for differentiating and transitioning into effector T cells. Such immune plasticity mounted a flexible and amplified response to TCE stimulation. Distinctively, non-responders had a higher prevalence of exhausted T cells at baseline, marked by expression of exhaustion markers, specifically TOX and other markers (GZMK, PD-1, LAG-3), thus limiting effective clonal expansion for immune cell engagement. Moreover, patients who initially responded to TCEs developed resistance later due to loss of MHC class I and reduced BCMA expression (192).

A real-world retrospective study of teclistamab revealed CD8^+^ T cells, specifically the effector memory population, as drivers of response and contrarily non-response aligned with a higher proportion of TIGIT^+^ Tregs and CD4^+^ central memory T cells (TCM) co-expressing TIGIT and PD-1 (207). AMG 701, a BCMA BsAb therapy, reiterated the significance of CD8^+^T cells, especially the central, effector, and stem-like memory subsets, in mediating a durable response and induced PD-1, IL-10, and Treg expression as a tumor resistance mechanism (189).

Cellular and molecular predictors of clinical response were determined using Cite-Seq profiling in MM patients treated with anti-BCMA CAR-T or BCMA-CD3 BsAbs at variable time points. The study endorsed the trend of fortified CD4^+^ T cells and a higher population of memory-like T cells, including stem cell memory T cells and central memory T cells in responders, identified through their specific phenotypic markers and transcriptional signatures. Non-responders maintained a terminally exhausted senescent T cell profile with an upregulation of Tregs and immune checkpoint inhibitors (LAG3, TIGIT, and PD-1) (208).

The role of Tregs in impeding response is apparent from the above studies, and a recent study has decoded the mechanisms of this resistance to TCE. The treatment with anti-BCMA BsAb expanded the Treg population with high expression of PD-1, TIGIT, and CD38 markers. Intriguingly, the BsAb therapy drove the differentiation of conventional CD4 T cells into induced (i) Tregs, characterized by persistent FOXP3 and CD25 expression, together with ICOS and CTLA-4 markers. These cells negatively regulate cytotoxic CD8 T cells through IL-10 upregulation, thus sustaining an immune-suppressed setting (209). Another indispensable population required for anti-BCMA BsAb efficiency is the induced (i) NKT cells, and Casey et al. ascertain the significance of these cells in synergy with IL-12 cytokine as critical players in response (210).

Talquetamab triggered robust T cell activation and degranulation, as evidenced by markers CD25 and CD107a on both CD4^+^ and CD8^+^ T cells. A dose-dependent increase of IFN-γ, TNF-α, IL-6, and IL-8 cytokines, as well as granzyme B, was seen post-treatment, further reflecting an activated immune microenvironment. On the contrary, Treg and PD-1 levels inversely correlate with talquetamab response, as they impair T cell activation and contribute to an exhausted suppressive tumor microenvironment. A high baseline proportion of T cells expressing PD-1 or HLA-DR negatively impacted its activity (15). This was further reaffirmed in the MonumenTAL-1 study, where responders showed robust, early T cell activation after talquetamab administration, characterized by higher and sustained levels of CD3^+^ T cells within the first two cycles versus non-responders and relapsed patients who exhibited persistent deficient immune competence due to increased expression of coinhibitory receptors and Tregs at progression (183).

An integrated study confirming resistance mechanisms to both teclistamab and talquetamab reported that Tregs and inhibitory receptors (PD-1, CTLA4, CD38) on CD4^+^ T cells are associated with primary resistance. Contrastingly, acquired resistance was fueled by exhaustion markers (PD-1, TIGIT, TIM-3), leading to defective T cell proliferation and cytokine secretion (205). Corroborating the above studies, Neri et al. showed selective expansion of clonotypic CD8^+^ T cells with minimal exhaustion, enriched pools of naive and memory CD8^+^ T cells in both peripheral blood and bone marrow of responders to TCEs. In addition, they exhibited increased tumor-reactive TCRs, with high clonality and reduced diversity in their TCR repertoires. Their hyperexpanded TCRs were linked to known tumor-reactive antigens, suggesting epitope expansion. Replacing bone marrow-exhausted T cell clones with non-dysfunctional circulating CD8^+^ T cells from peripheral blood validated TCE-induced immune restoration in responders. As documented earlier, exhausted, functionally compromised T cells with a less selective TCR repertoire were observed in non-responders (211).

In the case of BFCR4350A, a FCRL5 BsAb, higher and expanded CD8^+^ T cell activity in both peripheral blood and tumor sites predicted response (212). An effective immune synapse formation depends on the target FCRL5 clustering, and the exclusion of CD45 is vital for TCR signaling in anti-FCRL5/CD3 activity. TCEs that engage FCRL5 at membrane-proximal sites led to stronger immune synapse formation and enhanced MM cytotoxicity. As with other TCEs, the induction of exhaustion marker PD-1 also impaired responses to the FCRL5 targeting therapy (191).

For HPN217, the tri-specific TCE binds anti-BCMA for MM cell interaction, anti-albumin for half-life extension, and anti-CD3 for T cell activation, the potential anti-tumor activity was tolerable and scalable with reductions in sBCMA, upregulation of activation marker CD69 on CD8^+^ T cells, and cytokine engagement as key markers of response (95). Quantitative systems pharmacology modeling has delineated the drivers of an efficient response in a tri-specific TCE binding CD3 on T cells, CD38 on tumor cells, and CD28, a co-stimulatory receptor that triggers T cell activation. An enhanced T cell activation through CD28 co-stimulation and an increased and competent synapse formation with effective receptor occupancy promoted tumor cell cytotoxicity even at lower doses of the tri-specific TCE (213). Preclinical assessments of such a TCE affirmed a profile of low effector cells, high Tregs, and high TGF-β levels in poor responders, while responders had elevated cytotoxic and granzyme B^+^ T cells together with high concentrations of activation cytokines, IL-2, and IFN-γ (214, 215). The ex vivo biomarker analysis of tri-specific TCE, SAR442257, identified CD38 expression as the highest predictor of response (104).

This biomarker-based approach helps stratify patients more likely to benefit from T/NK cell-engaging therapies and proposes combination strategies to overcome resistance, ensuring more personalized and effective treatment plans for MM patients.

#### 5.2.3 Myeloid-derived suppressor cells

In MM, myeloid-derived suppressor cells (MSCs) create a supportive niche that helps myeloma cells survive, proliferate, and resist therapies. MSCs form a dense and protective niche around MM cells, posing a physical barrier and imparting an immunosuppressive microenvironment, thus diminishing immune cell engagement. Furthermore, MSCs weaken the potency of CD3 redirection BsAbs by suppressing immune effector cells’ activation and activating pro-survival pathways such as PI3K/Akt and Bcl-2 in MM cells (216). Direct contact and adhesion between MM cells and MSCs also contributed to talquetamab resistance, partly caused by decreased access to GPRC5D on MM cells (15). MSCs inhibited talquetamab-mediated lysis without diminishing T cell activation and degranulation, suggesting that MSCs induced cell-intrinsic resistance mechanisms within MM to counter the cytotoxic effects of T cells.

## 6 Combination strategies to improve patients’ response and overcome treatment toxicity

**Figure 4:**
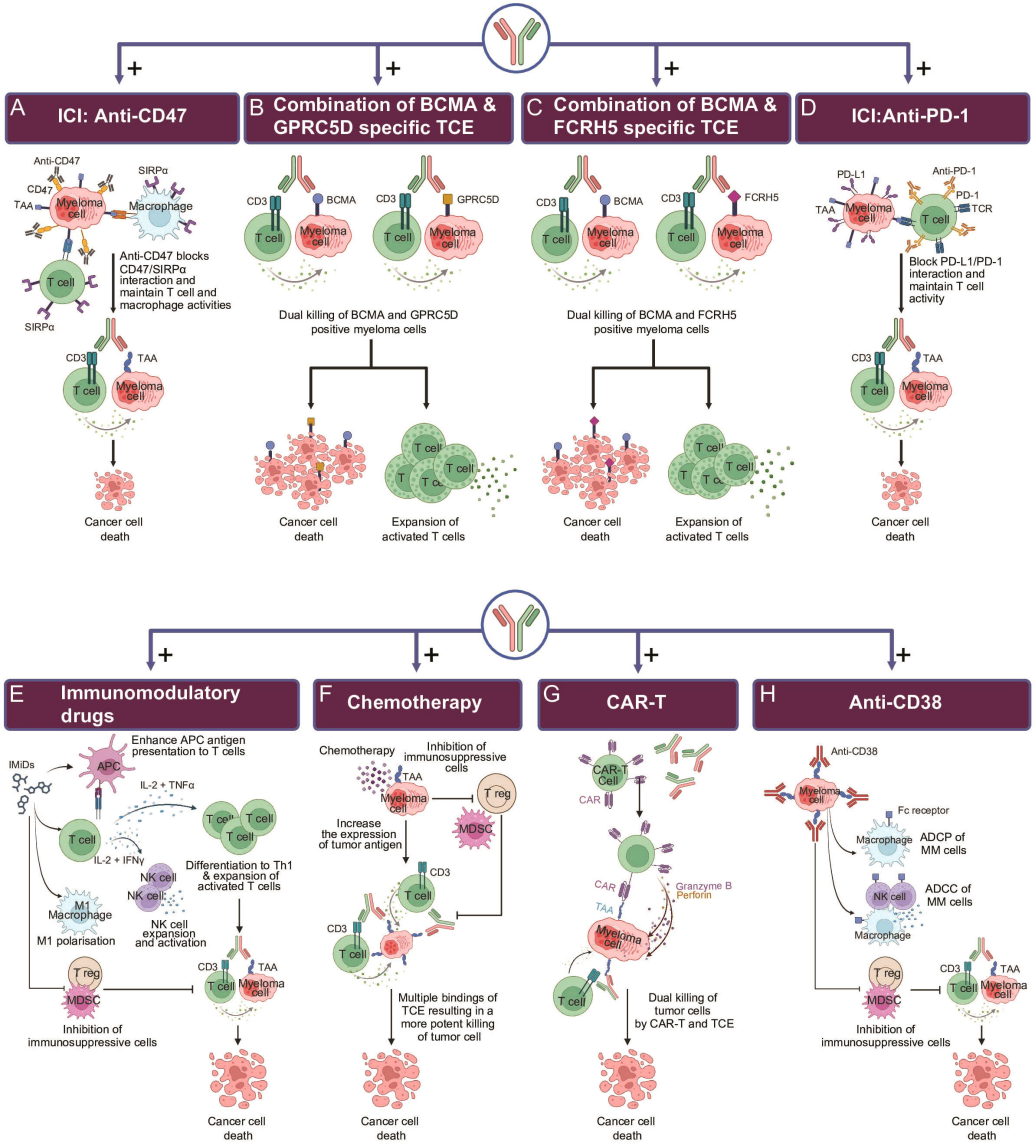
Combination therapies to enhance TCE efficacy. **(A)** Immune cell inhibitory mechanisms, such as CD47/SIRPα, can limit T or M cells activation by MsAbs. Anti-CD47 blocks the CD47/SIRPα signaling pathway, leading to enhanced T and M cytotoxicity and promoting the lysis and death of myeloma cells. **(B, C)** The combination of BCMA and GPC5D or FCRL5-specific TCE offers the benefit of targeting different tumor antigens simultaneously, resulting in more potent myeloma cell killing and T cell activation. **(D)** Immune cell inhibitory mechanisms, such as PD-L1/PD-1, can limit T cell activation by TCE. Anti-PD-1 blocks the PD-L1/PD-1 signaling pathway, leading to enhanced T cytotoxicity and promoting myeloma cell death. **(E)** IMiDs enhance APC antigen presentation to T cells. The activation of T cells induces the secretion of IL-2, IFN-γ, and TNF-α, leading to T/NK cell expansion and enhanced immune-mediated destruction of myeloma cells. The expansion of T cells enhances the interaction with TCE and promotes myeloma cell killing. IMiDs further increase anti-myeloma immunity through polarization of M towards an M1 tumoricidal phenotype, stimulation of dendritic cell antigen presentation, and downregulation of regulatory T cells (Treg) and myeloid-derived suppressor cells (MDSCs). IMiDs work synergistically with TCE to induce a more potent anti-myeloma activity. **(F)** Chemotherapy is known to increase the expression of tumor-associated antigens on myeloma cells. This feature will enhance the interaction of TCE with myeloma cells. Moreover, by downregulating Treg and MDSCs, chemotherapy promotes T cell activation, proliferation, and differentiation induced by TCE interaction. Chemotherapy supports TCE activity for a more potent anti-tumor effect. **(G)** Activated CAR-T cells can specifically recognize TAA working synergistically with T cells activated by TCE to induce a more potent anti-myeloma activity through the dual secretion of perforin and granzyme granules. **(H)** Dara recognizes CD38 on MM cells and exerts anti-MM activity via Fc-dependent mechanisms (ADCC and ADCP) and via immunomodulatory effects (reduction of immunosuppressive cells, Tregs, and MDSCs). Dara works synergistically with TCE to induce a more potent anti-myeloma activity.

Studies have investigated the potential of T, NK, and M engaging antibodies as effective combination therapies for MM. Several trials have tested these agents in combination with other BsAb or other anti-myeloma agents (190). A summary of ongoing trials of MsAbs in combination with other therapies is provided in **Supplementary Table 1**, **Figure 4**.

The RedirecTT-1 trial studied the combination of Talquetamab with Teclistamab in 228 patients with relapsed or refractory MM and extramedullary disease (NCT04586426). The combination induced an ORR in 80% of the advanced MM patients. This therapy regimen achieved a durable response in 86% of the patients after 18 months. However, the incidence of grade 3 or 4 infections with the combination was higher than that of monotherapy (32).

Another Phase 1 trial (NCT03269136) evaluated Elranatamab in combination with immunomodulatory drugs, including dexamethasone, lenalidomide, or pomalidomide, to assess dose-limiting toxicities and anti-myeloma activity. The study demonstrated encouraging results in achieving durable responses, manageable safety, and promising survival for patients with MM over two years (68, 217).

The Phase 1b MajesTEC-2 trial (NCT04722146) tested the safety, tolerability, and anti-myeloma activity of Teclistamab in combination with lenalidomide and the anti-CD38 daratumumab in RRMM. The ORR was seen in 13/13 patients after 8.61 months with a favorable safety profile (218). Based on these promising results, a phase 3 MajesTEC−7 study (NCT05552222) was designed to assess the efficacy of teclistamab or talquetamab in combination with daratumumab and lenalidomide versus daratumumab, lenalidomide, and dexamethasone in NDMM.

Preclinical studies demonstrated that the immune checkpoint blockade could enhance the efficacy of the immune cell engagers (219). Therefore, the combination of talquetamab with a PD-1 inhibitor is being tested in phase 1 TRIMM-3 trial in patients with RRMM (NCT05338775).

Other ongoing trials are examining the efficacy of T cell engagers such as Linvoseltamab (NCT05137054), Elranatamab (NCT05090566), and Teclistamab (NCT04722146) in combination with γ-secretase inhibitors. Indeed, the addition of γ-secretase inhibitor enhanced the anti-myeloma activity of BCMA BsAb *in vitro* through the reduction of sBCMA and an increase in BCMA expression on MM cells (199). More recently, the efficacy and safety of teclistamab in combination with a dendritic cell-based vaccine are being tested in RRMM patients (NCT06799026).

Furthermore, other combinations are being tested to reduce the toxicity of these multi-specific antibodies. For example, derma cosmetic products are being tested in combination with anti-GPRC5D bi-specific antibody to prevent and limit cutaneous and nail toxicity treatment-related adverse events (NCT06418750).

Collectively, these clinical trials provide critical data on optimizing combination therapies for MM. By focusing on efficacy, safety, and precise dosing strategies, these studies aim to advance MM treatment options, addressing resistance and reducing toxicity in this challenging therapeutic landscape.

## 7 Future directions

**Figure 5:**
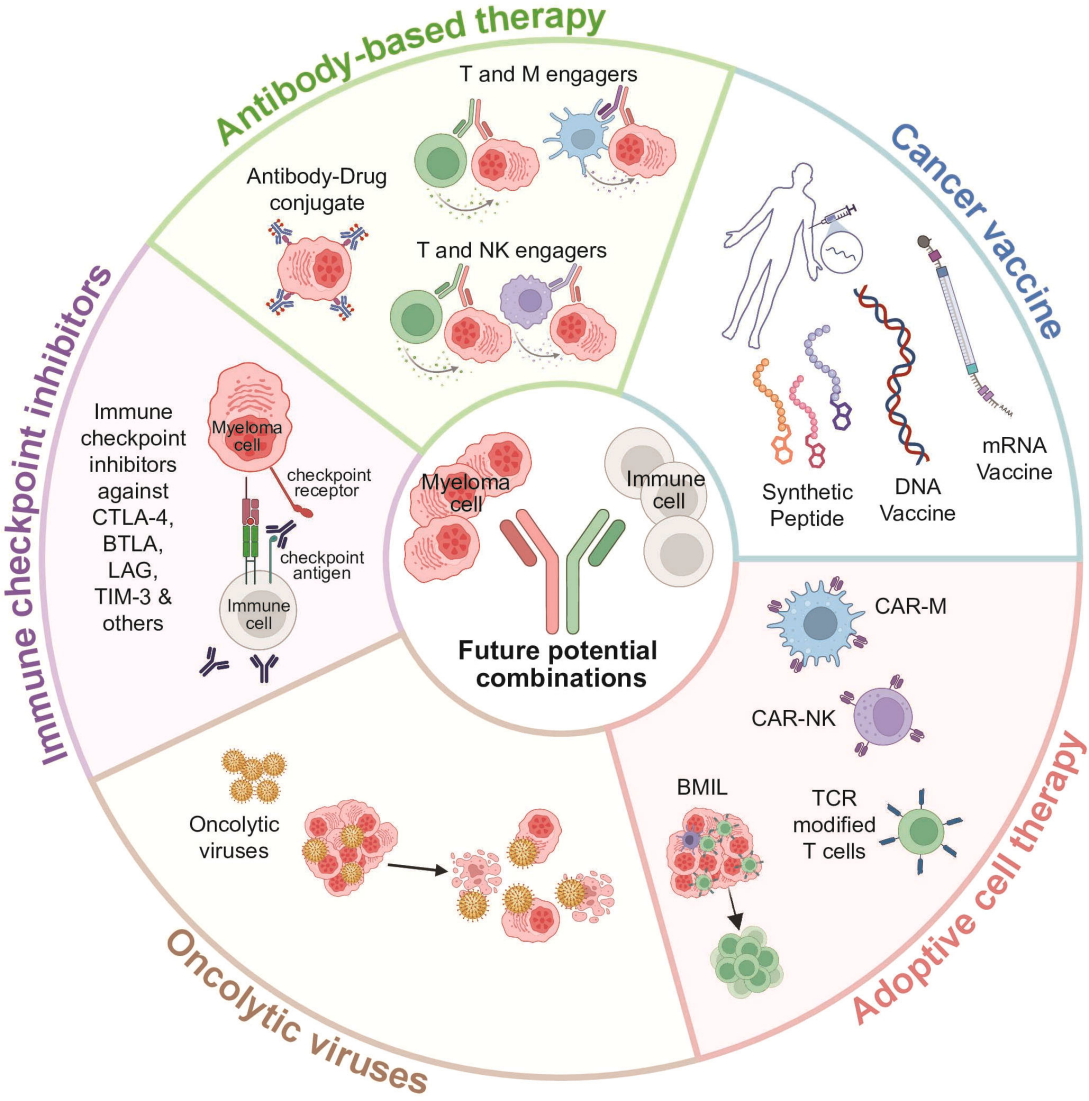
Future potential combination therapies for MsAbs. The image illustrates the potential combination strategies to improve treatment efficacy and overcome resistance in MM. Several therapeutic combinations with MsAbs are suggested. Antibody-based therapies include the combination of antibody drug conjugate (ADCs) with MsAbs or the simultaneous use of different MsAbs targeting the same or distinct tumor antigens. Cancer vaccines include the peptide-based, DNA-based, and mRNA-based vaccines targeting a tumor-specific antigen in combination with MsAbs. Adoptive cell therapy, including bone marrow infiltrating lymphocytes (BMILs), TCR-modified T cells, CAR-NK, and CAR-M, which can be tested in combination with MsAbs. Oncolytic viruses, which selectively infect and lyse tumor cells while enhancing antitumor immunity. Immune checkpoint inhibitors targeting inhibitory receptors such as CTLA-4, PD-1/PD-L1, LAG-3, TIM-3, and BTLA in combination with MsAbs could restore exhausted or impaired immune cells’ function.

In this part, we suggest several novel combinations that have not been previously tested to overcome some of the challenges discussed earlier. These combinations aim to enhance the anti-tumor efficacy of MsAbs while maintaining a relatively manageable safety profile (**Figure 5**). As mentioned earlier, the immunosuppressive tumor microenvironment represents a key challenge for immune engagers MsAbs treatment effectiveness and persistence. To address this challenge, combining MsAbs with immune checkpoint inhibitors targeting PD-L1, CTLA-4, LAG-3, TIM-3, TIGIT, CD47, the activating receptor natural killer group 2 member A (NKG2A), and others could present a promising strategy to remodel the tumor microenvironment, promote immune cells proliferation, cytotoxicity and sensitize TME cells to MsAbs. Several studies showed that combining such inhibitors with other standards of care enhances the antitumor efficacy *in vitro* and improves cancer patients’ survival (220–223). Recently, the FDA approved the use of different monoclonal antibodies targeting CTLA-4 (ipilimumab and tremelimumab), PD-L1 (atezolizumab, avelumab, and durvalumab), and LAG-3 (relatlimab) in different types of cancer (224). The evaluation of these immune checkpoint inhibitors alone or in combination with immune engagers MsAbs should be explored in future clinical trials to overcome resistance and improve overall outcomes in MM.

Oncolytic viruses are genetically engineered viruses that specifically target MM cells due to their overexpression of surface attachment receptors, including CD46 and CD138, abnormal homeostasis, and aberrant activation of RAS or Akt pathways (225). These features make MM hypersensitive to viral infection and replication compared to normal cells. The infection by oncolytic viruses induces MM cell death through apoptosis signaling and immune-mediated cellular destruction (226). Therefore, we suggest that oncolytic viruses could enhance MsAbs efficacy by fostering a more immunogenic microenvironment that increases cell death and tumor destruction. Notably, the treatment of PBMCs from MM patients with reovirus upregulated the activation marker CD69 expression on NK cells, CD4^+^ T cells, CD8^+^ T and NK, thus enhancing their cytotoxicity against autologous tumor cells (227). Interestingly, Kelly et al. reported that oncolytic virus treatment of MM cells increases the expression of PD-L1 (228). This upregulation could potentiate the anti-MM efficacy of anti-PD-L1 inhibitors in combination with MsAbs.

The immune system comprises innate immunity and adaptive immunity, which work synergistically to coordinate the antitumor immune response. T cells, NK, and M cells are the most effective cytotoxic immune cells in the tumor killing process (229). Notably, several studies highlighted the importance of the interaction and co-stimulation of T, NK, and M cells in tumor regression (229–232). Therefore, we suggest that the combination of T cell-based therapies (such as T cell engagers MsAbs or CAR-T, bone marrow infiltrating lymphocytes, and TCR engineered T cells) with NK/M cell-based therapies (including NK/M engagers MsAbs and CAR-NK/M) targeting the same or different tumor antigens could strengthen the immune response against tumors. Importantly, the dosing, timing, and sequence of drug administration of these combinations’ approaches are critical to both treatment efficacy and patient safety.

On the other hand, the integration of artificial intelligence (AI) methods and machine learning models could overcome the challenges previously discussed in this review. These methods can reduce time and costs, while minimize experimental failures and increase the success rate of antibody design compared to traditional approaches. AI-driven methodologies utilize large-scale databases including antibody sequences, structures, and interactions to model antibody structures, predict antibody–antigen interactions, optimize antibody affinity, and generate novel antibody candidates (233–235).

## 8 Conclusion

Multi-specific immune cell engagers, including bi-specific and tri-specific antibodies, have proven an impressive efficacy in treating MM, particularly in RRMM cases. While current research has primarily focused on RRMM patients, expanding studies to include newly diagnosed MM patients as first-line treatment, where the immune cell fitness is better compared with heavily pre-treated and relapsed or refractory patients, could further improve patients’ outcomes. Subsequently, several studies demonstrated the superior efficacy of these antibodies compared to MM conventional therapies. Indeed, the advancement of multi-specific antibodies could mark the beginning of a paradigm shift in myeloma treatment. However, this field still faces many challenges, such as optimizing treatment efficacy, optimizing the dose schedule, reducing toxicity, and overcoming resistance mechanisms etc of the MsAbs that are still under investigation and not yet FDA approved. These challenges could be addressed through the development of next-generation structures, innovative targets, and strategic treatment combinations. Furthermore, personalized medicine will be instrumental in optimizing therapeutic strategies by selecting the most suitable multi-specific immune engager based on patient-specific factors, including treatment history, molecular genetic profile, and immune phenotypic characteristics.

## Glossary

MM: Multiple Myeloma
MsAbs: Multi-Specific Antibodies
NK: Natural Killer
M: Macrophage
RRMM: Relapsed/Refractory Multiple Myeloma
BsAbs: Bi-specific Antibodies
TsAbs: Tri-specific Antibodies
CAR: Chimeric Antigen Receptor
TCE: T Cell Engaging
Ab: Antibody
FDA: Food and Drug Administration
BCMA: B Cell Maturation Antigen
TNFRSF: Tumor Necrosis Factor Receptor Superfamily
BAFF: B Cell Activating Factor
ORR: Overall Response Rate
MRD: Minimal Residual Disease
CRS: Cytokine Release Syndrome
sCR: Stringent Complete Response
NDMM: Newly Diagnosed Multiple Myeloma
PFS: Progression Free Survival
sBCMA: Soluble B Cell Maturation Antigen
GPRC5D: G Protein-Coupled Receptor Family C Member 5
VGPR: Very Good Partial Response
FCRL5: Fc Receptor-Like 5
HSA: Human Serum Albumin
SLAMF7: Signaling Lymphocytic Activation Molecule Family Member 7
NKCE: Natural Killer Cell Engager
MCE: Macrophage Cell Engager
ADCC: Antibody-Dependent Cellular Cytotoxicity
ADCP: Antibody-Dependent Cellular Phagocytosis
TNFα: Tumor Necrosis Family- alpha
IFN-γ: Interferon gamma
SIRPα: Signal Regulatory Protein Alpha
CDC: Complement-Dependent Cytotoxicity
Dara: Daratumumab
RBC: Red Blood Cell
TRAE: Treatment-Related Adverse Event
RRLCA: Relapsed/Refractory Light Chain Amyloidosis
IgG: Immunoglobulin G
MICA: MHC Class I-Related Chain A
EGFR: Epidermal Growth Factor Receptor
EpCAM: Epithelial Cell Adhesion Molecule
PD-1: Programmed Cell Death Protein 1
ICANS: Immune Effector Cell-Associated Neurotoxicity Syndrome
ANG2: Angiopoietin-2
GMCSF: Granulocyte-Macrophage Colony-Stimulating Factor
Cmax: Maximum Serum Concentration
SC: Subcutaneous
EMD: Extramedullary Disease
LAG-3: Lymphocyte-Activation Gene 3
TIM-3: T cell Immunoglobulin and Mucin-Domain Containing-3
TAA: Tumor-Associated Antigens
TME: Tumor Microenvironment
GZMK: Granzyme K
TCM: Memory T Cells
MSCs: Myeloid-Derived Suppressor Cells
NKG2A: Natural Killer Group 2 Member A
AI: Artificial Intelligence.

## References

[1] Morgan GJ Walker BA Davies FE . The genetic architecture of multiple myeloma. Nat Rev Cancer. (2012) 12:335–48. doi: 10.1038/nrc3257 22495321

[2] Rajkumar SV . Multiple myeloma: 2016 update on diagnosis, risk-stratification, and management. Am J Hematol. (2016) 91:719–34. doi: 10.1002/ajh.24402 27291302 PMC5291298

[3] Mafra A Laversanne M Marcos-Gragera R Chaves HVS McShane C Bray F . The global multiple myeloma incidence and mortality burden in 2022 and predictions for 2045. J Natl Cancer Inst. (2025) 117:907–14. doi: 10.1093/jnci/djae321 39658225

[4] Ocio EM Richardson PG Rajkumar SV Palumbo A Mateos MV Orlowski R . New drugs and novel mechanisms of action in multiple myeloma in 2013: a report from the International Myeloma Working Group (IMWG). Leukemia. (2014) 28:525–42. doi: 10.1038/leu.2013.350 24253022 PMC4143389

[5] Mateos MV Nooka AK Larson SM . Moving toward a cure for myeloma. Am Soc Clin Oncol Educ Book. (2022) 42:1–12. doi: 10.1200/edbk_349603 35623025

[6] Nakamura K Smyth MJ Martinet L . Cancer immunoediting and immune dysregulation in multiple myeloma. Blood. (2020) 136:2731–40. doi: 10.1182/blood.2020006540 32645135

[7] Russell BM Avigan DE . Immune dysregulation in multiple myeloma: the current and future role of cell-based immunotherapy. Int J Hematol. (2023) 117:652–9. doi: 10.1007/s12185-023-03579-x 36964840 PMC10039687

[8] Lopes R Caetano J Ferreira B Barahona F Carneiro EA Joao C . The immune microenvironment in multiple myeloma: friend or foe? Cancers (Basel). (2021) 13:625. doi: 10.3390/cancers13040625 33562441 PMC7914424

[9] Runcie K Budman DR John V Seetharamu N . Bi-specific and tri-specific antibodies- the next big thing in solid tumor therapeutics. Mol Med. (2018) 24:50. doi: 10.1186/s10020-018-0051-4 30249178 PMC6154901

[10] Tapia-Galisteo A Álvarez-Vallina L Sanz L . Bi- and trispecific immune cell engagers for immunotherapy of hematological Malignancies. J Hematol Oncol. (2023) 16:83. doi: 10.1186/s13045-023-01482-w 37501154 PMC10373336

[11] Cheng L Chen L Shi Y Gu W Ding W Zheng X . Efficacy and safety of bispecific antibodies vs. immune checkpoint blockade combination therapy in cancer: a real-world comparison. Mol Cancer. (2024) 23:77. doi: 10.1186/s12943-024-01956-6 38627681 PMC11020943

[12] Kim J Cho J Lee MH Yoon SE Kim WS Kim SJ . CAR T cells vs bispecific antibody as third- or later-line large B-cell lymphoma therapy: a meta-analysis. Blood. (2024) 144:629–38. doi: 10.1182/blood.2023023419 38696731

[13] Zhang M Lam KP Xu S . Natural killer cell engagers (NKCEs): a new frontier in cancer immunotherapy. Front Immunol. (2023) 14:1207276. doi: 10.3389/fimmu.2023.1207276 37638058 PMC10450036

[14] Pillarisetti K Powers G Luistro L Babich A Baldwin E Li Y . Teclistamab is an active T cell-redirecting bispecific antibody against B-cell maturation antigen for multiple myeloma. Blood Adv. (2020) 4:4538–49. doi: 10.1182/bloodadvances.2020002393 32956453 PMC7509877

[15] Verkleij CPM Broekmans MEC van Duin M Frerichs KA Kuiper R de Jonge AV . Preclinical activity and determinants of response of the GPRC5DxCD3 bispecific antibody talquetamab in multiple myeloma. Blood Adv. (2021) 5:2196–215. doi: 10.1182/bloodadvances.2020003805 33890981 PMC8095149

[16] Firestone R Lesokhin AM Usmani SZ . An embarrassment of riches: Three FDA-approved bispecific antibodies for relapsed refractory multiple myeloma. Blood Cancer Discov. (2023) 4:433–6. doi: 10.1158/2643-3230.bcd-23-0176 37824758 PMC10618718

[17] Moreau P Garfall AL NWCJvd D Nahi H San-Miguel JF Oriol A . Teclistamab in relapsed or refractory multiple myeloma. N Engl J Med. (2022) 387:495–505. doi: 10.1056/nejmoa2203478 35661166 PMC10587778

[18] Martin TG Mateos MV Nooka A Banerjee A Kobos R Pei L . Detailed overview of incidence and management of cytokine release syndrome observed with teclistamab in the MajesTEC-1 study of patients with relapsed/refractory multiple myeloma. Cancer. (2023) 129:2035–46. doi: 10.1002/cncr.34756 36991547

[19] Touzeau C Krishnan AY Moreau P Perrot A Usmani SZ Manier S . Efficacy and safety of teclistamab in patients with relapsed/refractory multiple myeloma after BCMA-targeting therapies. Blood. (2024) 144:2375–88. doi: 10.1182/blood.2023023616 39172760

[20] Miao X Wu LS Lin SXW Xu Y Chen Y Iwaki Y . Population pharmacokinetics and exposure-response with teclistamab in patients with relapsed/refractory multiple myeloma: results from MajesTEC-1. Target Oncol. (2023) 18:667–84. doi: 10.1007/s11523-023-00989-z 37713090 PMC10518021

[21] Cardé NAQ Niu J Guo Y Zhou J Qiu X Su Y . Revised recommendations for restarting teclistamab following dose delays: insights from the MajesTEC-1 study on clinical safety, and from simulated pharmacokinetics and cytokine dynamics. Targeted Oncol. (2026). doi: 10.1007/s11523-026-01205-4 PMC13222323 41886220

[22] Martin TG Mateos M-V Yi JH van de Donk NWCJ Cai Z Fu W . Efficacy and safety of teclistamab in triple-class exposed relapsed/refractory multiple myeloma: pooled findings from three clinical cohorts and a retrospective cohort. Cancer. (2026) 132:e70237. doi: 10.1002/cncr.70237 41485109

[23] Guo Y Niu J Cardé NAQ Wu L Miao X Hanson S . Teclistamab dosing in responders: modeling and simulation results from the MajesTEC-1 study in relapsed/refractory multiple myeloma. Targeted Oncol. (2025) 20:651–61. doi: 10.1007/s11523-025-01149-1 40332715 PMC12307566

[24] Moreau P Mateos MV Gonzalez Garcia ME Einsele H De Stefano V Karlin L . Comparative effectiveness of teclistamab versus real-world physician's choice of therapy in LocoMMotion and MoMMent in triple-class exposed relapsed/refractory multiple myeloma. Adv Ther. (2024) 41:696–715. doi: 10.1007/s12325-023-02738-0 38110653 PMC10838813

[25] Martin TG Moreau P Usmani SZ Garfall A Mateos MV San-Miguel JF . Teclistamab improves patient-reported symptoms and health-related quality of life in relapsed or refractory multiple myeloma: results from the phase II MajesTEC-1 study. Clin Lymphoma Myeloma Leuk. (2024) 24:194–202. doi: 10.1016/j.clml.2023.11.001 38052709

[26] Nooka AK Rodriguez C Mateos MV Manier S Chastain K Banerjee A . Incidence, timing, and management of infections in patients receiving teclistamab for the treatment of relapsed/refractory multiple myeloma in the MajesTEC-1 study. Cancer. (2024) 130:886–900. doi: 10.1002/cncr.35107 37960969

[27] Moreau P van de Donk NW Nahi H Oriol A Nooka AK Martin T . Plain language summary of the MajesTEC-1 study of teclistamab for the treatment of people with relapsed or refractory multiple myeloma. Future Oncol. (2023) 19:811–8. doi: 10.2217/fon-2023-0171 37132225

[28] Girgis S Lin SXW Pillarisetti K Banerjee A Stephenson T Ma X . Translational modeling predicts efficacious therapeutic dosing range of teclistamab for multiple myeloma. Target Oncol. (2022) 17:433–9. doi: 10.1007/s11523-022-00893-y 35749004 PMC9345835

[29] Usmani SZ Garfall AL van de Donk N Nahi H San-Miguel JF Oriol A . Teclistamab, a B-cell maturation antigen × CD3 bispecific antibody, in patients with relapsed or refractory multiple myeloma (MajesTEC-1): a multicenter, open-label, single-arm, phase 1 study. Lancet. (2021) 398:665–74. doi: 10.1016/s0140-6736(21)01338-6 34388396

[30] Moreau P van de Donk N Delforge M Einsele H De Stefano V Perrot A . Comparative efficacy of teclistamab versus current treatments in real-world clinical practice in the prospective LocoMMotion study in patients with triple-class-exposed relapsed and/or refractory multiple myeloma. Adv Ther. (2023) 40:2412–25. doi: 10.1007/s12325-023-02480-7 36961654 PMC10129954

[31] Cortes-Selva D Perova T Skerget S Vishwamitra D Stein S Boominathan R . Correlation of immune fitness with response to teclistamab in relapsed/refractory multiple myeloma in the MajesTEC-1 study. Blood. (2024) 144:615–28. doi: 10.1182/blood.2023022823 38657201 PMC11347796

[32] Cohen YC Magen H Gatt M Sebag M Kim K Min CK . Talquetamab plus teclistamab in relapsed or refractory multiple myeloma. N Engl J Med. (2025) 392:138–49. doi: 10.1056/nejmoa2406536 39778168

[33] Martin TG van de Donk N Rodríguez-Otero P Mateos MV Kobos R Chastain K . Plain language summary of the management of certain side effects of teclistamab in people with multiple myeloma. Future Oncol. (2026) 22:285–97. doi: 10.1080/14796694.2025.2597732 41574880 PMC12867346

[34] Frerichs KA Verkleij CPM Mateos MV Martin TG Rodriguez C Nooka A . Teclistamab impairs humoral immunity in patients with heavily pretreated myeloma: importance of immunoglobulin supplementation. Blood Adv. (2024) 8:194–206. doi: 10.1182/bloodadvances.2023011658 38052042 PMC10787247

[35] Pillarisetti K Edavettal S Mendonça M Li Y Tornetta M Babich A . A T-cell-redirecting bispecific G-protein-coupled receptor class 5 member D x CD3 antibody to treat multiple myeloma. Blood. (2020) 135:1232–43. doi: 10.1182/blood.2019003342 32040549 PMC7146017

[36] Chari A Touzeau C Schinke C Minnema MC Berdeja JG Oriol A . Safety and activity of talquetamab in patients with relapsed or refractory multiple myeloma (MonumenTAL-1): a multicenter, open-label, phase 1–2 study. Lancet Hematol. (2025) 12:e269–81. doi: 10.1016/s2352-3026(24)00385-5 40090350

[37] Chari A Minnema MC Berdeja JG Oriol A van de Donk N Rodríguez-Otero P . Talquetamab, a T-cell-redirecting GPRC5D bispecific antibody for multiple myeloma. N Engl J Med. (2022) 387:2232–44. doi: 10.1056/nejmoa2204591 36507686

[38] Einsele H Moreau P Bahlis N Bhutani M Vincent L Karlin L . Comparative efficacy of talquetamab vs. real-world physician's choice of treatment in triple-class-exposed relapsed/refractory multiple myeloma: updated analyses of MonumenTAL-1 vs. LocoMMotion/MoMMent. Adv Ther. (2026) 43:333–55. doi: 10.1007/s12325-025-03409-y 41313549 PMC12858606

[39] Schinke C Rodriguez-Otero P van de Donk N Lipe B Lavi N Rasche L . Infections and parameters of humoral immunity with talquetamab in relapsed/refractory multiple myeloma in MonumenTAL-1. Blood Adv. (2025) 9:5752–62. doi: 10.1182/bloodadvances.2025016613 40864183 PMC12661299

[40] Schinke C Touzeau C Oriol A Mateos MV Stevens D Rasche L . Talquetamab improves patient-reported symptoms and health-related quality of life in relapsed or refractory multiple myeloma: results from the phase 1/2 MonumenTAL-1 study. Cancer. (2025) 131:e35927. doi: 10.1002/cncr.35927 40631904 PMC12239699

[41] Catamero D Ray C Purcell K Leahey S Esler E Rogers S . Nursing considerations for the clinical management of adverse events associated with talquetamab in patients with relapsed or refractory multiple myeloma. Semin Oncol Nurs. (2024) 40:151712. doi: 10.1016/j.soncn.2024.151712 39155155

[42] Ito S Kuroda Y Sunami K Matsue K Imada K Tamura H . Talquetamab in Japanese patients with relapsed/refractory multiple myeloma in the MonumenTAL-1 study. Int J Hematol. (2026) 123:580–92. doi: 10.1007/s12185-025-04134-6 41405810 PMC13083461

[43] van de Donk N Chari A Martin T Krishnan A Rasche L Ye JC . Characterization and management of cytokine release syndrome from the MonumenTAL-1 study of talquetamab in patients with relapsed/refractory multiple myeloma. Cancer Med. (2025) 14:e71276. doi: 10.1002/cam4.71276 41036677 PMC12489744

[44] Einsele H Moreau P Bahlis N Bhutani M Vincent L Karlin L . Comparative efficacy of talquetamab vs. current treatments in the LocoMMotion and MoMMent studies in patients with triple-class-exposed relapsed/refractory multiple myeloma. Adv Ther. (2024) 41:1576–93. doi: 10.1007/s12325-024-02797-x 38402374 PMC10960754

[45] Lesokhin AM Tomasson MH Arnulf B Bahlis NJ Miles Prince H Niesvizky R . Elranatamab in relapsed or refractory multiple myeloma: phase 2 MagnetisMM-3 trial results. Nat Med. (2023) 29:2259–67. doi: 10.1038/s41591-023-02528-9 37582952 PMC10504075

[46] Gordan LN Bensimon AG Mu F Kim N Wu B Lin D . Cost per responder for teclistamab and elranatamab in relapsed or refractory multiple myeloma in the United States. J Med Econ. (2025) 28:910–20. doi: 10.1080/13696998.2025.2514909 40495704

[47] Hebraud B Charalampous C Parrondo R Chhabra S Nagaraj M DiBonaventura M . Comparison of patient-reported outcomes for elranatamab in MagnetisMM-3 versus real-world physician choice of therapy for triple-class refractory multiple myeloma. Clin Lymphoma Myeloma Leuk. (2026). doi: 10.1016/j.clml.2026.03.014 41968022

[48] Elmeliegy M Soltantabar P Hibma J Ashman O Wang D Lon HK . Elranatamab fixed dosing: a safe, effective, and convenient dosing approach. Target Oncol. (2025) 20:821–31. doi: 10.1007/s11523-025-01170-4 40830740 PMC12454520

[49] Elmeliegy M Viqueira A Vandendries E Hickman A Conte U Irby D . Dose optimization of elranatamab to mitigate the risk of cytokine release syndrome in patients with multiple myeloma. Target Oncol. (2025) 20:349–59. doi: 10.1007/s11523-025-01134-8 40000533 PMC11933221

[50] Mol I Hu Y LeBlanc TW Cappelleri JC Chu H Nador G . A matching-adjusted indirect comparison of the efficacy of elranatamab versus physician's choice of treatment in patients with triple-class exposed/refractory multiple myeloma. Curr Med Res Opin. (2024) 40:199–207. doi: 10.1182/blood-2023-185920 38078866

[51] Iida S Ito S Yokoyama H Ishida T Nagai Y Handa H . Elranatamab in Japanese patients with relapsed/refractory multiple myeloma: results from MagnetisMM-2 and MagnetisMM-3. Jpn J Clin Oncol. (2024) 54:991–1000. doi: 10.1093/jjco/hyae068 38794892 PMC11374885

[52] Lon H-K Hibma J Jiang S Sullivan S Vandendries E Skoura A . Population exposure–response efficacy analysis of elranatamab (PF-06863135) in patients with multiple myeloma. Targeted Oncol. (2025) 20:803–19. doi: 10.1007/s11523-025-01168-y 40826257 PMC12454538

[53] Costa LJ LeBlanc TW Tesch H Sonneveld P Kyle RP Sinyavskaya L . Elranatamab efficacy in MagnetisMM-3 compared with real-world control arms in triple-class refractory multiple myeloma. Future Oncol. (2024) 20:1175–89. doi: 10.2217/fon-2023-0995 38415370 PMC11318743

[54] Iida S Ito S Yokoyama H Ishida T Nagai Y Handa H . Elranatamab in Japanese patients with relapsed/refractory multiple myeloma: results from MagnetisMM-2 and MagnetisMM-3. Jpn J Clin Oncol. (2024) 54:991–1000. doi: 10.1093/jjco/hyae068 38794892 PMC11374885

[55] Bumma N Richter J Jagannath S Lee HC Hoffman JE Suvannasankha A . Linvoseltamab for treatment of relapsed/refractory multiple myeloma. J Clin Oncol. (2024) 42:2702–12. doi: 10.1200/jco.24.01008 38879802 PMC11272139

[56] Lee HC Zonder JA Dhodapkar MV Jagannath S Hoffman JE Suvannasankha A . Linvoseltamab in patients with relapsed/refractory multiple myeloma in the LINKER-MM1 study: longer follow-up and subgroup analyses. Clin Lymphoma Myeloma Leukemia. (2026) 26:e201–e212.e8. doi: 10.1016/j.clml.2025.11.004 41387038

[57] Eckmann J Fauti T Biehl M Zabaleta A Blanco L Lelios I . Forimtamig, a novel GPRC5D-targeting T-cell bispecific antibody with a 2 + 1 format, for the treatment of multiple myeloma. Blood. (2025) 145:202–19. doi: 10.1182/blood.2024025987 39476124 PMC11738037

[58] Carretero-Iglesia L Hall OJ Berret J Pais D Estoppey C Chimen M . ISB 2001 trispecific T cell engager shows strong tumor cytotoxicity and overcomes immune escape mechanisms of multiple myeloma cells. Nat Cancer. (2024) 5:1494–514. doi: 10.1038/s43018-024-00821-1 39261676 PMC11505469

[59] Wang JX Qi JY Li WY Zhai ZM Li P Zou W . GNC-038, a tetra-specific antibody, in patients with R/R non-Hodgkin lymphoma or acute lymphoblastic leukemia: a phase 1 study design and rationale. J Clin Oncol. (2023) 41:TPS2668–TPS2668. doi: 10.1891/9780826155146.0036 41472467

[60] Hu S Fu W Xu W Yang Y Cruz M Berezov SD . Four-in-one antibodies have superior cancer inhibitory activity against EGFR, HER2, HER3, and VEGF through disruption of HER/MET crosstalk. Cancer Res. (2015) 75:159–70. doi: 10.1016/b978-0-12-821584-5.00012-2 25371409

[61] Shah N Chari A Scott E Mezzi K Usmani SZ . B-cell maturation antigen (BCMA) in multiple myeloma: rationale for targeting and current therapeutic approaches. Leukemia. (2020) 34:985–1005. doi: 10.1038/s41375-020-0734-z 32055000 PMC7214244

[62] Dogan A Siegel D Tran N Fu A Fowler J Belani R . B-cell maturation antigen expression across hematologic cancers: a systematic literature review. Blood Cancer J. (2020) 10:73. doi: 10.1038/s41408-020-0337-y 32606424 PMC7327051

[63] Garfall AL Nooka AK NWCJvd D Moreau P Bhutani M Oriol A . Long-term follow-up from the phase 1/2 MajesTEC-1 trial of teclistamab in patients with relapsed/refractory multiple myeloma. J Clin Oncol. (2024) 42:7540. doi: 10.1016/s2152-2650(24)00953-4

[64] Firestone R Shekarkhand T Patel D Tan CRC Hultcrantz M Lesokhin AM . Evaluating the efficacy of commercial teclistamab in relapsed refractory multiple myeloma patients with prior exposure to anti-BCMA therapies. J Clin Oncol. (2023) 41:8049. doi: 10.1200/jco.2023.41.16_suppl.8049 41909186

[65] Qureshi Z Jamil A Altaf F Siddique R Ahmed F . Efficacy and safety of teclistamab in relapsed or refractory multiple myeloma: a systematic review and meta-analysis. Ann Hematol. (2024) 103:4901–12. doi: 10.1007/s00277-024-06078-z 39511034

[66] Costa LJ Bahlis NJ Perrot A Nooka AK Lu J Pawlyn C . Teclistamab plus daratumumab in relapsed or refractory multiple myeloma. N Engl J Med. (2026) 394:739–52. doi: 10.1056/nejmoa2514663 41363801 PMC13218738

[67] Tomasson MH Iida S Niesvizky R Mohty M Bahlis NJ Martinez-Lopez J . Long-term survival and safety of elranatamab in patients with relapsed or refractory multiple myeloma: update from the MagnetisMM-3 study. Hemasphere. (2024) 8:e136. doi: 10.1002/hem3.136 39055646 PMC11269363

[68] Bahlis NJ Costello CL Raje NS Levy MY Dholaria B Solh M . Elranatamab in relapsed or refractory multiple myeloma: the MagnetisMM-1 phase 1 trial. Nat Med. (2023) 29:2570–6. doi: 10.1038/s41591-023-02589-w 37783970 PMC10579053

[69] Lee K Zhou Y Franklin M Ullman E Hermann A Kroog GS . Characterization of linvoseltamab's BCMA binding epitope and efficacy against BCMA mutations in relapsed/refractory multiple myeloma. Blood. (2024) 144:3265. doi: 10.1182/blood-2024-208776

[70] Suvannasankha A Kapoor P Pianko MJ Richter J D'Souza A Anderson LD . Abstract CT013: Safety and efficacy from the phase 1/2 first-in-human study of REGN5459, a BCMA×CD3 bispecific antibody with low CD3 affinity, in patients with relapsed/refractory multiple myeloma. Cancer Res. (2023) 83:CT013–3. doi: 10.1158/1538-7445.am2023-ct013 36230740

[71] Kumar S Jagannath S Weisel KC Dachs LR Dimopoulos MA Siegel DS . Comparative effectiveness of linvoseltamab versus current real-world standard of care therapies in triple-class exposed relapsed/refractory multiple myeloma treated at IMWG sites. Blood. (2024) 144:7039–41. doi: 10.1182/blood-2024-210376

[72] DiLillo DJ Olson K Mohrs K Meagher TC Bray K Sineshchekova O . A BCMAxCD3 bispecific T cell-engaging antibody demonstrates robust antitumor efficacy similar to that of anti-BCMA CAR T cells. Blood Adv. (2021) 5:1291–304. doi: 10.1182/bloodadvances.2020002736 33651100 PMC7948265

[73] Voorhees PM D'Souza A Weisel K Hurd DD Teipel R Chung A . A phase 1 first-in-human study of Abbv-383, a BCMA x CD3 bispecific T-cell-redirecting antibody, as monotherapy in patients with relapsed/refractory multiple myeloma. Blood. (2022) 140:4401–4. doi: 10.1182/blood-2022-167008 PMC9622641 36029527

[74] Sharon D Robinson V Hecquet C Calabrese K Cosgrove C Mantis C . Bivalent BCMA binding and low affinity CD3 T-cell engagement by Abbv-383 drives sustained activation with reduced T-cell exhaustion in preclinical models of multiple myeloma. Blood. (2023) 142:4666. doi: 10.1182/blood-2023-179039

[75] Vij R Kumar SK D'Souza A Mckay JT Voorhees PM Chung A . Updated safety and efficacy results of Abbv-383, a BCMA x CD3 bispecific T-cell redirecting antibody, in a first-in-human phase 1 study in patients with relapsed/refractory multiple myeloma. Blood. (2023) 142:3378. doi: 10.1182/blood-2023-182388

[76] Sun MY Qiu LG Wei YQ Jin J Li X Liu X . Results from a first-in-human phase I study of F182112, a B-cell maturation antigen (BCMA)-CD3 bispecific antibody, in patients with relapsed/refractory multiple myeloma. J Clin Oncol. (2023) 41:8038. doi: 10.1200/jco.2023.41.16_suppl.8038 41909186

[77] Bar P Mateos MV Ribas P Hansson M Paris L Hofmeister CC . Alnuctamab (ALNUC; BMS-986349; CC-93269), a 2 + 1 B-cell maturation antigen (BCMA) x CD3 T-cell engager (TCE), administered subcutaneously (SC) in patients (pts) with relapsed/refractory multiple myeloma (RRMM): updated results from a phase 1 first-in-human clinical study. Blood. (2023) 142:2011. doi: 10.1182/blood-2022-159009

[78] Jakubowiak AJ Anguille S Karlin L Chari A Schinke C Rasche L . Updated results of talquetamab, a GPRC5D×CD3 bispecific antibody, in patients with relapsed/refractory multiple myeloma with prior exposure to T-cell redirecting therapies: results of the phase 1/2 MonumenTAL-1 study. Blood. (2023) 142:3377. doi: 10.1182/blood-2023-187242

[79] Al Hadidi S Szabo A Mohan Lal B Bag A Zhen S Ogunsesan Y . Talquetamab in relapsed refractory multiple myeloma: multi-institutional real-world study. Blood Cancer J. (2025) 15:196. doi: 10.1038/s41408-025-01386-7 41203605 PMC12594773

[80] Carlo-Stella C Mazza R Manier S Facon T Yoon SS Koh Y . RG6234, a GPRC5DxCD3 T-cell engaging bispecific antibody, is highly active in patients (pts) with relapsed/refractory multiple myeloma (RRMM): updated intravenous (IV) and first subcutaneous (SC) results from a phase I dose-escalation study. Blood. (2022) 140:397–9. doi: 10.1182/blood-2022-157988

[81] Wang B Huang X Sun J Qin Y Shang H Li T . Lbl-034, a highly differentiated T-cell engaging bispecific antibody targeting GPRC5D for the treatment of relapsed or refractory multiple myeloma. Blood. (2023) 142:4672. doi: 10.1182/blood-2023-181395

[82] Qin L Zhong YP Huang DP Xie HT Du X Liao AJ . An uniquely designed asymmetric bispecific antibody against GPRC5D and CD3 (LBL-034) in patients with relapsed/refractory multiple myeloma: A phase I/II, first in human, open-label, multicenter, dose escalation/expansion study. Blood. (2024) 144:4736–7. doi: 10.1182/blood-2024-202871

[83] Jiang D Huang H Qin H Tang K Shi X Zhu T . Chimeric antigen receptor T cells targeting FcRH5 provide robust tumor-specific responses in murine xenograft models of multiple myeloma. Nat Commun. (2023) 14:3642. doi: 10.1038/s41467-023-39395-4 37339964 PMC10282100

[84] Trudel S Cohen AD Krishnan AY Fonseca R Spencer A Berdeja JG . Cevostamab monotherapy continues to show clinically meaningful activity and manageable safety in patients with heavily pre-treated relapsed/refractory multiple myeloma (RRMM): Updated results from an ongoing phase I study. Blood. (2021) 138:157. doi: 10.1182/blood-2021-147983

[85] Lesokhin AM Richter J Trudel S Cohen AD Spencer A Forsberg PA . Enduring responses after 1-year, fixed-duration cevostamab therapy in patients with relapsed/refractory multiple myeloma: Early experience from a phase I study. Blood. (2022) 140:4415–7. doi: 10.1182/blood-2022-157547

[86] Mateos M-V Bahlis NJ Spencer A Kaedbey R Rodríguez-Otero P Harrison S . P946: Tocilizumab pre-treatment significantly reduces the incidence of cytokine release syndrome in patients with relapsed/refractory multiple myeloma (Rrmm) who receive cevostamab. HemaSphere. (2023) 7:e75458a8. doi: 10.1097/01.hs9.0000970688.75458.a8 33079766

[87] Kowalski A Lykon J Diamond B Coffey D Kaddoura M Maura F . Tocilizumab prophylaxis for patients with multiple myeloma treated with bispecific antibodies. Blood Adv. (2025) 9:4979–86. doi: 10.1182/bloodadvances.2025016911 40590849 PMC12514479

[88] Kumar S Bachier CR Cavo M Corradini P Delforge M Janowski W . CAMMA 2: A phase I/II trial evaluating the efficacy and safety of cevostamab in patients with relapsed/refractory multiple myeloma (RRMM) who have triple-class refractory disease and have received a prior anti-B-cell maturation antigen (BCMA) agent. J Clin Oncol. (2023) 41:TPS8064–TPS8064. doi: 10.1200/jco.2023.41.16_suppl.tps8064 41909186

[89] Pouleau B Estoppey C Suere P Nallet E Laurendon A Monney T . Preclinical characterization of ISB 1342, a CD38 x CD3 T-cell engager for relapsed/refractory multiple myeloma. Blood. (2023) 142:260–73. doi: 10.1182/blood.2022019451 37192303 PMC10644056

[90] Mohan SR Chase CC Berdeja JG Karlin L Belhadj K Perrot A . Initial results of dose escalation of ISB 1342, a novel CD3xCD38 bispecific antibody, in patients with relapsed/refractory multiple myeloma (RRMM). Blood. (2022) 140:7264–6. doi: 10.1182/blood-2022-157525

[91] Kapoor P Stevens DA Leleu X Merzoug KB Karlin L Manier S . Dose escalation of ISB 1342, a novel CD38xCD3 bispecific antibody, in patients with relapsed / refractory multiple myeloma (RRMM). Blood. (2023) 142:3339. doi: 10.1182/blood-2023-186664

[92] Chen X Wong OK Reiman L Sherbenou DW Post L . CD38 x ICAM-1 bispecific antibody is a novel approach for treating multiple myeloma and lymphoma. Mol Cancer Ther. (2024) 23:127–38. doi: 10.1158/1535-7163.mct-23-0052 37816503

[93] Zuch de Zafra CL Fajardo F Zhong W Bernett MJ Muchhal US Moore GL . Targeting multiple myeloma with AMG 424, a novel anti-CD38/CD3 bispecific T-cell-recruiting antibody optimized for cytotoxicity and cytokine release. Clin Cancer Res. (2019) 25:3921–33. doi: 10.1158/1078-0432.ccr-18-2752 30918018

[94] Li KY Yun R Chai M Yakkundi P Rosete R Li G . Igm-2644, a novel CD38xCD3 bispecific IgM T cell engager demonstrates potent efficacy on myeloma cells with an improved preclinical safety profile. Blood. (2022) 140:6010–1. doi: 10.1182/blood-2022-159205

[95] Abdallah AO Cowan AJ Leleu X Touzeau C Lipe B Medvedova E . Updated interim results from a phase 1 study of HPN217, a half-life extended tri-specific T cell activating construct (TriTAC®) targeting B cell maturation antigen (BCMA) for relapsed/refractory multiple myeloma (RRMM). Blood. (2022) 140:7284–5. doi: 10.1182/blood-2022-159665

[96] Madan S Costello CL Lipe B Cowan AJ Medvedova E Hillengass J . Results from the completed dose escalation portion of the phase 1 study of HPN217, a half-life extended tri-specific T cell activating construct (TriTAC) targeting B cell maturation antigen (BCMA) for relapsed/refractory multiple myeloma (MM). Blood. (2023) 142:1012. doi: 10.1182/blood-2023-182345

[97] Pillarisetti R Yang DL Yao JH Smith M Luistro L Vulfson P . Characterization of JNJ-79635322, a novel BCMAxGPRC5DxCD3 T-cell redirecting trispecific antibody, for the treatment of multiple myeloma. Blood. (2023) 142:456. doi: 10.1182/blood-2023-174941

[98] Akhmetzyanova I McCarron MJ Parekh S Chesi M Bergsagel PL Fooksman DR . Dynamic CD138 surface expression regulates switch between myeloma growth and dissemination. Leukemia. (2020) 34:245–56. doi: 10.1038/s41375-019-0519-4 31439945 PMC6923614

[99] Chen D Zou J Zong Y Meng H An G Yang L . Anti-human CD138 monoclonal antibodies and their bispecific formats: Generation and characterization. Immunopharmacol Immunotoxicol. (2016) 38:175–83. doi: 10.3109/08923973.2016.1153110 26954291

[100] Frebel K Albring JC Wohlgemuth A Schwoppe C Hailfinger S Lenz G . Comparison of antibody-based immunotherapeutics for Malignant hematological disease in an experimental murine model. Blood Adv. (2024) 8:1934–45. doi: 10.1182/bloodadvances.2023011647 38197968 PMC11021910

[101] van Rhee F Szmania SM Zhan F Gupta SK Pomtree M Lin P . NY-ESO-1 is highly expressed in poor-prognosis multiple myeloma and induces spontaneous humoral and cellular immune responses. Blood. (2005) 105:3939–44. doi: 10.1182/blood-2004-09-3707 15671442 PMC1895070

[102] Maruta M Ochi T Tanimoto K Asai H Saitou T Fujiwara H . Direct comparison of target-reactivity and cross-reactivity induced by CAR- and BiTE-redirected T cells for the development of antibody-based T-cell therapy. Sci Rep. (2019) 9:13293. doi: 10.1038/s41598-019-49834-2 31527633 PMC6746725

[103] Geis M Nowotny B Bohn MD Kouhestani D Einsele H Bumm T . Combinatorial targeting of multiple myeloma by complementing T cell engaging antibody fragments. Commun Biol. (2021) 4:44. doi: 10.1038/s42003-020-01558-0 33420283 PMC7794243

[104] Keller AL Reiman LT Perez de Acha O Parzych SE Forsberg PA Kim PS . Ex vivo efficacy of SAR442257 anti-CD38 trispecific T-cell engager in multiple myeloma relapsed after daratumumab and BCMA-targeted therapies. Cancer Res Commun. (2024) 4:757–64. doi: 10.1158/2767-9764.crc-23-0434 38421887 PMC10929583

[105] Rolin C Zimmer J Seguin-Devaux C . Bridging the gap with multispecific immune cell engagers in cancer and infectious diseases. Cell Mol Immunol. (2024) 21:643–61. doi: 10.1038/s41423-024-01176-4 38789528 PMC11214628

[106] Zhu A Bai Y Nan Y Ju D . Natural killer cell engagers: From bi-specific to tri-specific and tetra-specific engagers for enhanced cancer immunotherapy. Clin Transl Med. (2024) 14:e70046. doi: 10.1002/ctm2.70046 39472273 PMC11521791

[107] Chen S Saeed A Liu Q Jiang Q Xu H Xiao GG . Macrophages in immunoregulation and therapeutics. Signal Transduct Target Ther. (2023) 8:207. doi: 10.1038/s41392-023-01452-1 37211559 PMC10200802

[108] Lian G Mak TS Yu X Lan HY . Challenges and recent advances in NK cell-targeted immunotherapies in solid tumors. Int J Mol Sci. (2021) 23. doi: 10.3390/ijms23010164 35008589 PMC8745474

[109] Li W Wang F Guo R Bian Z Song Y . Targeting macrophages in hematological Malignancies: Recent advances and future directions. J Hematol Oncol. (2022) 15:110. doi: 10.1186/s13045-022-01328-x 35978372 PMC9387027

[110] Romee R Rosario M Berrien-Elliott MM Wagner JA Jewell BA Schappe T . Cytokine-induced memory-like natural killer cells exhibit enhanced responses against myeloid leukemia. Sci Transl Med. (2016) 8:357ra123. doi: 10.1126/scitranslmed.aaf2341 27655849 PMC5436500

[111] André P Denis C Soulas C Bourbon-Caillet C Lopez J Arnoux T . Anti-NKG2A mAb is a checkpoint inhibitor that promotes anti-tumor immunity by unleashing both T and NK cells. Cell. (2018) 175:1731–1743.e13. doi: 10.1016/j.cell.2018.10.014 30503213 PMC6292840

[112] Winidmanokul P Panya A Okada S . Tri-specific killer engager: Unleashing multi-synergic power against cancer. Explor Target Antitumor Ther. (2024) 5:432–48. doi: 10.37349/etat.2024.00227 38745768 PMC11090690

[113] Kakiuchi-Kiyota S Ross T Wallweber HA Kiefer JR Schutten MM Adedeji AO . A BCMA/CD16A bispecific innate cell engager for the treatment of multiple myeloma. Leukemia. (2022) 36:1006–14. doi: 10.1038/s41375-021-01478-w 35001074

[114] Kakiuchi-Kiyota S Schutten MM Adedeji AO Cai H Hendricks R Liu L . Abstract 4556: Preclinical pharmacology and safety of RO7297089, a novel anti-BCMA/CD16a bispecific antibody for the treatment of multiple myeloma. Cancer Res. (2020) 80:4556. doi: 10.1158/1538-7445.am2020-4556 36127472

[115] Cai H Kakiuchi-Kiyota S Hendricks R Zhong S Liu L Adedeji AO . Nonclinical pharmacokinetics, pharmacodynamics, and translational model of RO7297089, a novel anti-BCMA/CD16A bispecific tetravalent antibody for the treatment of multiple myeloma. AAPS J. (2022) 24:100. doi: 10.1208/s12248-022-00744-8 36127472

[116] Plesner T Harrison SJ Quach H Lee C Bryant A Vangsted A . Phase I study of safety and pharmacokinetics of RO7297089, an anti-BCMA/CD16a bispecific antibody, in patients with relapsed, refractory multiple myeloma. Clin Hematol Int. (2023) 5:43–51. doi: 10.1007/s44228-022-00023-5 36656461 PMC10063703

[117] Plesner T Harrison SJ Quach H Lee CH Bryant A Vangsted AJ . A phase I study of RO7297089, a B-cell maturation antigen (BCMA)-CD16a bispecific antibody in patients with relapsed/refractory multiple myeloma (RRMM). Blood. (2021) 138:2755. doi: 10.1182/blood-2021-147418

[118] Grandclement C Estoppey C Dheilly E Panagopoulou M Monney T Dreyfus C . Development of ISB 1442, a CD38 and CD47 bispecific biparatopic antibody innate cell modulator for the treatment of multiple myeloma. Nat Commun. (2024) 15:2054. doi: 10.1038/s41467-024-46310-y 38448430 PMC10917784

[119] Stefano S Grandclement C Dehilly E Panagopoulou M Martini E Castillo R . ISB 1442, a first-in-class CD38 and CD47 bispecific antibody innate cell modulator for the treatment of relapsed refractory multiple myeloma. Blood. (2021) 138:73. doi: 10.1182/blood-2021-145586

[120] Kim D Wang J Willingham SB Martin R Wernig G Weissman IL . Anti-CD47 antibodies promote phagocytosis and inhibit the growth of human myeloma cells. Leukemia. (2012) 26:2538–45. doi: 10.1038/leu.2012.141 22648449

[121] Chao MP Takimoto CH Feng DD McKenna K Gip P Liu J . Therapeutic targeting of the macrophage immune checkpoint CD47 in myeloid Malignancies. Front Oncol. (2019) 9:1380. doi: 10.3389/fonc.2019.01380 32038992 PMC6990910

[122] Yang H Xun Y You H . The landscape overview of CD47-based immunotherapy for hematological Malignancies. biomark Res. (2023) 11:15. doi: 10.1186/s40364-023-00456-x 36726125 PMC9893585

[123] Sikic BI Lakhani N Patnaik A Shah SA Chandana SR Rasco D . First-in-human, first-in-class phase I trial of the anti-CD47 antibody Hu5F9-G4 in patients with advanced cancers. J Clin Oncol. (2019) 37:946–53. doi: 10.1200/jco.18.02018 30811285 PMC7186585

[124] Kazandjian D Quach H Sia H Ho PJ Spencer A Schroeder MA . Initial dose escalation of ISB 1442, a novel CD38 biparatopic x CD47 bispecific antibody, in patients with relapsed / refractory multiple myeloma (RRMM). Blood. (2023) 142:4707. doi: 10.1182/blood-2023-186241

[125] Tang A Gauthier L Beninga J Rossi B Gourdin N Blanchard-Alvarez A . The novel trifunctional anti-BCMA NK cell engager SAR'514 has potent in-vitro and in-vivo anti-myeloma effect through dual NK cell engagement. Blood. (2022) 140:9985–6. doi: 10.1182/blood-2022-166187

[126] Tang A Gauthier L Zaghi E Beninga J Amara C Basset A . Targeting BCMA in multiple myeloma with a trifunctional NK cell engager. Cell Rep Med. (2026) 7:102628. doi: 10.1016/j.xcrm.2026.102628 41709452 PMC13006415

[127] Gantke T Reusch U Kellner C Ellwanger K Fucek I Weichel M . AFM26 is a novel, highly potent BCMA/CD16A-directed bispecific antibody for high affinity NK-cell engagement in multiple myeloma. J Clin Oncol. (2017) 35:8045. doi: 10.1200/jco.2017.35.15_suppl.8045 41909186

[128] Gantke T Reusch U Kellner C Klausz K Haneke T Knackmuss S . AFM26-targeting B cell maturation antigen (BCMA) for NK cell-mediated immunotherapy of multiple myeloma. Blood. (2017) 130:3082. doi: 10.1182/blood.V130.Suppl_1.3082.3082

[129] Ross T Reusch U Wingert S Haneke T Klausz K Otte AK . Preclinical characterization of AFM26, a novel B cell maturation antigen (BCMA)-directed tetravalent bispecific antibody for high affinity retargeting of NK cells against myeloma. Blood. (2018) 132:1927. doi: 10.1182/blood-2018-99-118970

[130] Asaadi Y Jouneghani FF Janani S Rahbarizadeh F . A comprehensive comparison between camelid nanobodies and single chain variable fragments. biomark Res. (2021) 9:87. doi: 10.1186/s40364-021-00332-6 34863296 PMC8642758

[131] Ye QN Zhu L Liang J Zhao DK Tian TY Fan YN . Orchestrating NK and T cells via tri-specific nano-antibodies for synergistic antitumor immunity. Nat Commun. (2024) 15:6211. doi: 10.1038/s41467-024-50474-y 39043643 PMC11266419

[132] Yang XM Lin XD Shi W Xie SX Huang XN Yin SH . Nanobody-based bispecific T-cell engager (Nb-BiTE): a new platform for enhanced T-cell immunotherapy. Signal Transduct Target Ther. (2023) 8:328. doi: 10.1038/s41392-023-01523-3 37661200 PMC10475457

[133] Giang KA Boxaspen T Diao Y Nilvebrant J Kosugi-Kanaya M Kanaya M . Affibody-based hBCMA x CD16 dual engagers for NK cell-mediated killing of multiple myeloma cells. N Bio/Technol. (2023) 77:139–48. doi: 10.1016/j.nbt.2023.09.002 37673373

[134] Blackman J Blackman JT Pauranik A . A case report supporting the use of teclistamab in multiple myeloma with CNS involvement. Case Rep Hematol. (2025) 2025:8425047. doi: 10.1155/crh/8425047 41194784 PMC12585788

[135] Noveihed A Al Hadidi S Mohan M Shah MR . Bispecific antibody therapy in CNS myeloma: Early evidence from a multicenter cohort. Br J Haematol. (2025) 207:2161–6. doi: 10.1111/bjh.70108 41251392 PMC12624187

[136] Draghi M Schafer JL Nelson A Frye Z Oliphant A Haserlat S . Abstract 4972: Preclinical development of a first-in-class NKp30xBCMA NK cell engager for the treatment of multiple myeloma. Cancer Res. (2019) 79:4972. doi: 10.1158/1538-7445.am2019-4972 36230740

[137] Watkins-Yoon J Guzman W Oliphant A Haserlat S Leung A Chottin C . CTX-8573, an innate-cell engager targeting BCMA, is a highly potent multispecific antibody for the treatment of multiple myeloma. Blood. (2019) 134:3182. doi: 10.1182/blood-2019-128749

[138] Peipp M Derer S Lohse S Staudinger M Klausz K Valerius T . HER2-specific immunoligands engaging NKp30 or NKp80 trigger NK-cell-mediated lysis of tumor cells and enhance antibody-dependent cell-mediated cytotoxicity. Oncotarget. (2015) 6:32075–88. doi: 10.18632/oncotarget.5135 26392331 PMC4741660

[139] Zwirner NW Fuertes MB Girart MV Domaica CI Rossi LE . Cytokine-driven regulation of NK cell functions in tumor immunity: role of the MICA-NKG2D system. Cytokine Growth Factor Rev. (2007) 18:159–70. doi: 10.1016/j.cytogfr.2007.01.013 17324607

[140] Xing S Ferrari de Andrade L . NKG2D and MICA/B shedding: a 'tag game' between NK cells and Malignant cells. Clin Transl Immunol. (2020) 9:e1230. doi: 10.1002/cti2.1230 33363734 PMC7754731

[141] Wang Y Li H Xu W Pan M Qiao C Cai J . BCMA-targeting bispecific antibody that simultaneously stimulates NKG2D-enhanced efficacy against multiple myeloma. J Immunother. (2020) 43:175–88. doi: 10.1097/cji.0000000000000320 32349046

[142] Xu W Wang Y Li H Wang M Zhang J . BCMA targets bispecific fusion protein for induction of NK cell activity against multiple myeloma. (2021). doi: 10.21203/rs.3.rs-351031/v1

[143] Khairnar V Lin L Chang HM Maiti S Diaz S Mandelboim O . CYT-338 NK cell engager mutispecific antibody engagement of NKp46 activation receptor overcomes anti-CD38 Mab mediated fratricide of NK cells and accords enhanced cancer immunotherapy. Blood. (2023) 142:6725. doi: 10.1182/blood-2023-190613

[144] Lin L Chang HM Wu D Titz B Chen A Ma MC . Biological characterization and differential gene expression analysis of CYT-338 NK cell engager (NKE) against CD38 expressing tumors including multiple myeloma (MM). Blood. (2022) 140:7068–9. doi: 10.1182/blood-2022-170193

[145] Therapeutics, N . Available online at: https://www.nayatx.com/copy-of-about-us (Accessed June 6, 2026).

[146] Chan WK Kang S Youssef Y Glankler EN Barrett ER Carter AM . A CS1-NKG2D bispecific antibody collectively activates cytolytic immune cells against multiple myeloma. Cancer Immunol Res. (2018) 6:776–87. doi: 10.1158/2326-6066.cir-17-0649 29769244 PMC6030494

[147] Chen S Lai SWT Brown CE Feng M . Harnessing and enhancing macrophage phagocytosis for cancer therapy. Front Immunol. (2021) 12:635173. doi: 10.3389/fimmu.2021.635173 33790906 PMC8006289

[148] Andersen MH . Tumor microenvironment antigens. Semin Immunopathol. (2023) 45:253–64. doi: 10.1007/s00281-022-00966-0 36175673 PMC10335965

[149] van de Donk NW Bahlis N Mateos MV Weisel K Dholaria B Garfall AL . S183: Novel combination immunotherapy for the treatment of relapsed/refractory multiple myeloma: Updated phase 1b results for talquetamab (a Gprc5d x Cd3 bispecific antibody) in combination with daratumumab. HemaSphere. (2022) 6:84–5. doi: 10.1097/01.hs9.0000843624.82943.92 33079766

[150] Hasselbalch Riley C Hutchings M Yoon SS Koh Y Manier S Facon T . S180: Rg6234, a novel Gprc5d T-cell engaging bispecific antibody, induces rapid responses in patients with relapsed/refractory multiple myeloma: Preliminary results from a first-in-human trial. HemaSphere. (2022) 6:81–2. doi: 10.1097/01.hs9.0000843612.41180.42 33079766

[151] Chari A Touzeau C Schinke C Minnema MC Berdeja J Oriol A . Talquetamab, a G protein-coupled receptor family C group 5 member D x CD3 bispecific antibody, in patients with relapsed/refractory multiple myeloma (RRMM): Phase 1/2 results from MonumenTAL-1. Blood. (2022) 140:384–7. doi: 10.1182/blood-2022-159707

[152] Zhao P Schardt J Chiang CI Shah P Eun GS Martinek J . Improving dual targeting selectivity in T-cell engagers via synapse-gated and affinity-tuned trispecific antibody design. MAbs. (2025) 17:2570748. doi: 10.1080/19420862.2025.2570748 41058481 PMC12520116

[153] Cattaruzza F Nazeer A To M Hammond M Koski C Liu LY . Precision-activated T-cell engagers targeting HER2 or EGFR and CD3 mitigate on-target, off-tumor toxicity for immunotherapy in solid tumors. Nat Cancer. (2023) 4:485–501. doi: 10.1038/s43018-023-00536-9 36997747 PMC10132983

[154] Boedtkjer E Pedersen SF . The acidic tumor microenvironment as a driver of cancer. Annu Rev Physiol. (2020) 82:103–26. doi: 10.1146/annurev-physiol-021119-034627 31730395

[155] Frey G Cugnetti APG Liu H Xing C Wheeler C Chang HW . A novel conditional active biologic anti-EpCAM x anti-CD3 bispecific antibody with synergistic tumor selectivity for cancer immunotherapy. MAbs. (2024) 16:2322562. doi: 10.1080/19420862.2024.2322562 38445633 PMC10936661

[156] Guo ZS Lotze MT Zhu Z Storkus WJ Song XT . Bi- and tri-specific T cell engager-armed oncolytic viruses: Next-generation cancer immunotherapy. Biomedicines. (2020) 8. doi: 10.3390/biomedicines8070204 32664210 PMC7400484

[157] Tang TF Cheong CS Looi CY Wong WF Gan GG . Mechanistic insights and advances of bispecific T cell engaging antibodies therapy in multiple myeloma. Med (Kaunas). (2025) 61. doi: 10.3390/medicina61122113 41470115 PMC12734887

[158] van Faassen H Jo DH Ryan S Lowden MJ Raphael S MacKenzie CR . Incorporation of a novel CD16-specific single-domain antibody into multispecific natural killer cell engagers with potent ADCC. Mol Pharm. (2021) 18:2375–84. doi: 10.1021/acs.molpharmaceut.1c00208 33999642

[159] Bannas P Hambach J Koch-Nolte F . Nanobodies and nanobody-based human heavy chain antibodies as antitumor therapeutics. Front Immunol. (2017) 8:1603. doi: 10.3389/fimmu.2017.01603 29213270 PMC5702627

[160] Zaman R Islam RA Ibnat N Othman I Zaini A Lee CY . Current strategies in extending half-lives of therapeutic proteins. J Control Release. (2019) 301:176–89. doi: 10.1016/j.jconrel.2019.02.016 30849445

[161] Hambach J Fumey W Stahler T Gebhardt AJ Adam G Weisel K . Half-life extended nanobody-based CD38-specific bispecific killercell engagers induce killing of multiple myeloma cells. Front Immunol. (2022) 13:838406. doi: 10.3389/fimmu.2022.838406 35651607 PMC9150782

[162] Ding Z Sun S Wang X Yang X Shi W Huang X . Nanobody-based trispecific T cell engager (Nb-TriTE) enhances therapeutic efficacy by overcoming tumor-mediated immunosuppression. J Hematol Oncol. (2023) 16:115. doi: 10.1186/s13045-023-01507-4 38031188 PMC10688028

[163] Zhao Y Han B Hao JL Zheng YD Chai JS Zhang ZZ . Bi-specific macrophage nano-engager for cancer immunotherapy. Nano Today. (2021) 41:101313. doi: 10.1016/j.nantod.2021.101313 38826717

[164] Pan D Richter J . Management of toxicities associated with BCMA, GPRC5D, and FcRH5-targeting bispecific antibodies in multiple myeloma. Curr Hematol Malig Rep. (2024) 19:237–45. doi: 10.1007/s11899-024-00740-z 39145912

[165] Mohan M Chakraborty R Bal S Nellore A Baljevic M D'Souza A . Recommendations on prevention of infections during chimeric antigen receptor T-cell and bispecific antibody therapy in multiple myeloma. Br J Haematol. (2023) 203:736–46. doi: 10.1111/bjh.18909 37287117 PMC10700672

[166] Ludwig H Terpos E van de Donk N Mateos MV Moreau P Dimopoulos MA . Prevention and management of adverse events during treatment with bispecific antibodies and CAR T cells in multiple myeloma: a consensus report of the European Myeloma Network. Lancet Oncol. (2023) 24:e255–69. doi: 10.1016/s1470-2045(23)00159-6 37269857

[167] Hassel JC Berking C Forschner A Gebhardt C Heinzerling L Meier F . Practical guidelines for the management of adverse events of the T cell engager bispecific tebentafusp. Eur J Cancer. (2023) 191:112986. doi: 10.1016/j.ejca.2023.112986 37595494

[168] Pelland AA Dumas M Lemieux-Blanchard É LeBlanc R Côté J Boudreault JS . Perspectives on outpatient delivery of bispecific T-cell engager therapies for multiple myeloma. Curr Oncol. (2025) 32. doi: 10.3390/curroncol32040238 40277794 PMC12025952

[169] Leclercq G Steinhoff N Haegel H De Marco D Bacac M Klein C . Novel strategies for the mitigation of cytokine release syndrome induced by T cell engaging therapies with a focus on the use of kinase inhibitors. Oncoimmunology. (2022) 11:2083479. doi: 10.1080/2162402x.2022.2083479 35694193 PMC9176235

[170] Li H Er Saw P Song E . Challenges and strategies for next-generation bispecific antibody-based antitumor therapeutics. Cell Mol Immunol. (2020) 17:451–61. doi: 10.1038/s41423-020-0417-8 32313210 PMC7193592

[171] Autissier P Soulas C Burdo TH Williams KC . Evaluation of a 12-color flow cytometry panel to study lymphocyte, monocyte, and dendritic cell subsets in humans. Cytometry A. (2010) 77:410–9. doi: 10.1002/cyto.a.20859 20099249 PMC11742174

[172] Cui A Huang T Li S Ma A Perez JL Sander C . Dictionary of immune responses to cytokines at single-cell resolution. Nature. (2024) 625:377–84. doi: 10.1038/s41586-023-06816-9 38057668 PMC10781646

[173] Sokol CL Luster AD . The chemokine system in innate immunity. Cold Spring Harb Perspect Biol. (2015) 7. doi: 10.1101/cshperspect.a016303 25635046 PMC4448619

[174] Huan T Guan B Li H Tu X Zhang C Tang B . Principles and current clinical landscape of NK cell engaging bispecific antibody against cancer. Hum Vaccin Immunother. (2023) 19:2256904. doi: 10.1080/21645515.2023.2256904 37772505 PMC10543353

[175] Chiu E Felices M Cichocki F Davis Z Wang H Tuninga K . Anti-NKG2C/IL-15/anti-CD33 killer engager directs primary and iPSC-derived NKG2C(+) NK cells to target myeloid leukemia. Mol Ther. (2021) 29:3410–21. doi: 10.1016/j.ymthe.2021.06.018 34174441 PMC8636174

[176] Soltantabar P Sharma S Wang D Lon HK Czibere A Hickmann A . Impact of treatment modality and route of administration on cytokine release syndrome in relapsed or refractory multiple myeloma: A meta-analysis. Clin Pharmacol Ther. (2024) 115:1258–68. doi: 10.1002/cpt.3223 38459622

[177] Tacchetti P Barbato S Mancuso K Zamagni E Cavo M . Bispecific antibodies for the management of relapsed/refractory multiple myeloma. Cancers (Basel). (2024) 16. doi: 10.3390/cancers16132337 39001399 PMC11240369

[178] Aguiar-Ibanez R Fotheringham I Mittal L Sillah A Pathak S . Differences between intravenous and subcutaneous modes of administration in oncology from the patient, healthcare provider, and healthcare system perspectives: A systematic review. Adv Ther. (2024) 41:4396–417. doi: 10.1007/s12325-024-02985-9 39425890

[179] Lee H Ahn S Maity R Leblay N Ziccheddu B Truger M . Mechanisms of antigen escape from BCMA- or GPRC5D-targeted immunotherapies in multiple myeloma. Nat Med. (2023) 29:2295–306. doi: 10.1038/s41591-023-02491-5 37653344 PMC10504087

[180] Meermeier EW Welsh SJ Sharik ME Du MT Garbitt VM Riggs DL . Tumor burden limits bispecific antibody efficacy through T cell exhaustion averted by concurrent cytotoxic therapy. Blood Cancer Discov. (2021) 2:354–69. doi: 10.1158/2643-3230.bcd-21-0038 34258584 PMC8266040

[181] Rees MJ Mammadzadeh A Bolarinwa A Elhaj ME Bohra A Bansal R . Clinical features associated with poor response and early relapse following BCMA-directed therapies in multiple myeloma. Blood Cancer J. (2024) 14:122. doi: 10.1038/s41408-024-01081-z 39043638 PMC11266661

[182] Zonder JA Richter J Bumma N Brayer J Hoffman JE Bensinger WI . Early, deep, and durable responses, and low rates of cytokine release syndrome with REGN5458, a BCMAxCD3 bispecific monoclonal antibody, in a phase 1/2 first-in-human study in patients with relapsed/refractory multiple myeloma (RRMM). Blood. (2021) 138:160. doi: 10.1182/blood-2021-144921 33831168

[183] Vishwamitra D Skerget S Cortes D Lau O Davis C Renaud T . Mechanisms of resistance and relapse with talquetamab in patients with relapsed/refractory multiple myeloma from the phase 1/2 MonumenTAL-1 study. Blood. (2023) 142:1933. doi: 10.1182/blood-2023-187755

[184] Mouhieddine TH Van Oekelen O Melnekoff DT Li J Ghodke-Puranik Y Lancman G . Sequencing T-cell redirection therapies leads to deep and durable responses in patients with relapsed/refractory myeloma. Blood Adv. (2023) 7:1056–64. doi: 10.1182/bloodadvances.2022007923 36018226 PMC10033902

[185] Puertas B Fernandez-Sanchez A Alejo E Rey-Bua B Martin-Lopez AA Perez-Lopez E . A research center's experience of T-cell-redirecting therapies in triple-class refractory multiple myeloma. Blood Adv. (2024) 8:3478–87. doi: 10.1182/bloodadvances.2024012773 38717869 PMC11260841

[186] Frerichs KA Broekmans MEC Marin Soto JA van Kessel B Heymans MW Holthof LC . Preclinical activity of JNJ-7957, a novel BCMAxCD3 bispecific antibody for the treatment of multiple myeloma, is potentiated by daratumumab. Clin Cancer Res. (2020) 26:2203–15. doi: 10.1158/1078-0432.ccr-19-2299 31969333

[187] Letouzé E Moreau P Munshi N Samur M Minvielle S Touzeau C . Mechanisms of resistance to bispecific T-cell engagers in multiple myeloma and their clinical implications. Blood Adv. (2024) 8:2952–9. doi: 10.1182/bloodadvances.2023012354 PMC11302375 38513088

[188] Dhodapkar MV . Immune status and selection of patients for immunotherapy in myeloma: a proposal. Blood Adv. (2024) 8:2424–32. doi: 10.1182/bloodadvances.2023011242 38564776 PMC11112605

[189] Goldstein RL Goyos A Li CM Deegen P Bogner P Sternjak A . AMG 701 induces cytotoxicity of multiple myeloma cells and depletes plasma cells in cynomolgus monkeys. Blood Adv. (2020) 4:4180–94. doi: 10.1182/bloodadvances.2020002565 32886754 PMC7479952

[190] Tomita U Ishimoto Y Ri M Kawase Y Hizukuri Y Maru C . A novel T cell-redirecting anti-GPRC5D x CD3 bispecific antibody with potent antitumor activity in multiple myeloma preclinical models. Sci Rep. (2024) 14:5135. doi: 10.1038/s41598-024-55143-0 38429446 PMC10907593

[191] Li J Stagg NJ Johnston J Harris MJ Menzies SA DiCara D . Membrane-proximal epitope facilitates efficient T cell synapse formation by anti-FcRH5/CD3 and is a requirement for myeloma cell killing. Cancer Cell. (2017) 31:383–95. doi: 10.1016/j.ccell.2017.02.001 28262555 PMC5357723

[192] Friedrich MJ Neri P Kehl N Michel J Steiger S Kilian M . The pre-existing T cell landscape determines the response to bispecific T cell engagers in multiple myeloma patients. Cancer Cell. (2023) 41:711–25. doi: 10.1016/j.ccell.2023.02.008 36898378

[193] Girgis S Wang Lin SX Pillarisetti K Verona R Vieyra D Casneuf T . Effects of teclistamab and talquetamab on soluble BCMA levels in patients with relapsed/refractory multiple myeloma. Blood Adv. (2023) 7:644–8. doi: 10.1182/bloodadvances.2022007625 36006441 PMC9979748

[194] Lee H Durante M Skerget S Vishwamitra D Benaoudia S Ahn S . Impact of soluble BCMA and non-T-cell factors on refractoriness to BCMA-targeting T-cell engagers in multiple myeloma. Blood. (2024) 144:2637–51. doi: 10.1182/blood.2024026212 39321344 PMC11738017

[195] Topp MS Duell J Zugmaier G Attal M Moreau P Langer C . Anti-B-cell maturation antigen BiTE molecule AMG 420 induces responses in multiple myeloma. J Clin Oncol. (2020) 38:775–83. doi: 10.1200/jco.19.02657 31895611

[196] Wiedemann A Szita VR Horvath R Szederjesi A Sebo A Toth AD . Soluble B-cell maturation antigen as a monitoring marker for multiple myeloma. Pathol Oncol Res. (2023) 29:1611171. doi: 10.3389/pore.2023.1611171 37188125 PMC10178067

[197] Firestone RS Socci ND Shekarkhand T Zhu M Qin WG Hultcrantz M . Antigen escape as a shared mechanism of resistance to BCMA-directed therapies in multiple myeloma. Blood. (2024) 144:402–7. doi: 10.1182/blood.2023023557 38728378 PMC11302451

[198] Borkowski A Giordano U Szlasa W Dudek K Kędziora K Mordak-Domagała M . Soluble B-cell maturation antigen as a prognostic marker for progression-free survival in multiple myeloma treated with BCMA-directed therapies: a systematic review and meta-analysis. Cancers. (2026) 18:686. doi: 10.3390/cancers18040686 41749939 PMC12938959

[199] Chen H Yu T Lin L Xing L Cho SF Wen K . Gamma-secretase inhibitors augment efficacy of BCMA-targeting bispecific antibodies against multiple myeloma cells without impairing T-cell activation and differentiation. Blood Cancer J. (2022) 12:118. doi: 10.1038/s41408-022-00716-3 35973981 PMC9381512

[200] Laurie PC Molloy ME Ng PP Anand BS . Abstract 5081: Anti-tumor activity of HPN217, a BCMA-targeting tri-specific T cell engager, is enhanced by γ-secretase inhibitors in preclinical models. Cancer Res. (2023) 83:5081. doi: 10.1158/1538-7445.am2023-5081 36230740

[201] Derrien J Gastineau S Frigout A Giordano N Cherkaoui M Gaborit V . Acquired resistance to a GPRC5D-directed T-cell engager in multiple myeloma is mediated by genetic or epigenetic target inactivation. Nat Cancer. (2023) 4:1536–43. doi: 10.1038/s43018-023-00625-9 37653140

[202] Truger MS Duell J Zhou X Heimeshoff L Ruckdeschel A John M . Single- and double-hit events in genes encoding immune targets before and after T cell-engaging antibody therapy in MM. Blood Adv. (2021) 5:3794–8. doi: 10.1182/bloodadvances.2021004418 34471932 PMC8679680

[203] Papadimitriou M Ahn S Diamond BT Lee H McIntyre JB Truger M . Timing genomic antigen loss in multiple myeloma treated with T cell-redirecting immunotherapies. Blood Cancer Discov. (2025) 6:572–9. doi: 10.1158/2643-3230.bcd-25-0005 40906971 PMC12514755

[204] Papadimitriou M Ahn S Diamond B Lee H McIntyre J Truger M . Target expression, generation, preclinical activity, and pharmacokinetics of the BCMA-T cell bispecific antibody EM801 for multiple myeloma treatment. Cancer Cell. (2017) 31:396–410. doi: 10.1016/j.ccell.2017.02.002 28262554

[205] Verkleij CPM O'Neill CA Broekmans MEC Frerichs KA Bruins WSC Duetz C . T-cell characteristics impact response and resistance to T-cell-redirecting bispecific antibodies in multiple myeloma. Clin Cancer Res. (2024) 30:3006–22. doi: 10.1158/1078-0432.ccr-23-3333 38687588

[206] Pihlgren M Hall O Carretero L Estoppey C Drake A Pais D . ISB 2001, a first-in-class trispecific BCMA and CD38 T cell engager designed to overcome mechanisms of escape from treatments for multiple myeloma by targeting two antigens. Blood. (2022) 140:858–9. doi: 10.1182/blood-2022-159353

[207] Firestone RS McAvoy D Shekarkhand T Serrano E Hamadeh I Wang A . CD8 effector T cells enhance teclistamab response in BCMA-exposed and -naive multiple myeloma. Blood Adv. (2024) 8:1600–11. doi: 10.1182/bloodadvances.2023011225 37878808 PMC10987849

[208] Leblay N Maity R Barakat E McCulloch S Duggan P Jimenez-Zepeda V . Cite-Seq profiling of T cells in multiple myeloma patients undergoing BCMA targeting CAR-T or Bites immunotherapy. Blood. (2020) 136:11–2. doi: 10.1182/blood-2020-137650

[209] Casey M Lee C Kwok WY Law SC Corvino D Gandhi MK . Regulatory T cells hamper the efficacy of T-cell-engaging bispecific antibody therapy. Hematologica. (2024) 109:787–98. doi: 10.3324/haematol.2023.283758 37767564 PMC10905103

[210] Casey M Tu C Harrison SJ Nakamura K . Invariant NKT cells dictate antitumor immunity elicited by a bispecific antibody cotargeting CD3 and BCMA. Blood Adv. (2022) 6:5165–70. doi: 10.1182/bloodadvances.2022008118 35830292 PMC9631633

[211] Neri P Ahn S Lee H Leblay N Friedrich M Maity R . Dysfunctional hyper-expanded clonotypes and lack of TCR clonal replacement predict resistance to T cell engagers in multiple myeloma. Blood. (2022) 140:2093–4. doi: 10.1182/blood-2022-164717

[212] Nakamura R Lear S Wilson D Koeppen H Vaze A Trudel S . Early pharmacodynamic changes in T-cell activation, proliferation, and cytokine production confirm the mode of action of BFCR4350A, a FcRH5/CD3 T-cell-engaging bispecific antibody, in patients with relapsed/refractory multiple myeloma. Blood. (2020) 136:14–5. doi: 10.1182/blood-2020-136980

[213] Abrams RE Pierre K El-Murr N Seung E Wu L Luna E . Quantitative systems pharmacology modeling sheds light into the dose response relationship of a trispecific T cell engager in multiple myeloma. Sci Rep. (2022) 12:10976. doi: 10.1038/s41598-022-14726-5 35768621 PMC9243109

[214] Grab AL Kim PS John L Bisht K Wang H Baumann A . Pre-clinical assessment of SAR442257, a CD38/CD3xCD28 trispecific T cell engager in treatment of relapsed/refractory multiple myeloma. Cells. (2024) 13. doi: 10.3390/cells13100879 38786100 PMC11120574

[215] Wu L Seung E Xu L Rao E Lord DM Wei RR . Trispecific antibodies enhance the therapeutic efficacy of tumor-directed T cells through T cell receptor co-stimulation. Nat Cancer. (2020) 1:86–98. doi: 10.1038/s43018-019-0004-z 35121834

[216] Nair-Gupta P Rudnick SI Luistro L Smith M McDaid R Li Y . Blockade of VLA4 sensitizes leukemic and myeloma tumor cells to CD3 redirection in the bone marrow microenvironment. Blood Cancer J. (2020) 10:65. doi: 10.1038/s41408-020-0331-4 32483120 PMC7264144

[217] Dalovisio A Bahlis N Raje N Costello C Dholaria B Solh M . P897: Updated results from the ongoing phase 1 study of elranatamab, a Bcma targeted T-cell redirecting immunotherapy, for patients with relapsed or refractory multiple myeloma. HemaSphere. (2022) 6:788–9. doi: 10.1097/01.hs9.0000846460.44039.3d 33079766

[218] Searle E Quach H Wong SW Costa LJ Hulin C Janowski W . Teclistamab in combination with subcutaneous daratumumab and lenalidomide in patients with multiple myeloma: results from one cohort of MajesTEC-2, a phase1b, multicohort study. Blood. (2022) 140:394–6. doi: 10.1182/blood-2022-159711

[219] Mouhieddine TH Vieira J Aleman A Grossman L Melnekoff D Upadhyaya B . Combined PD-1 and Tim-3 blockade improves the anti-myeloma activity of T cells of patients on talquetamab. Blood. (2024) 144:357–8. doi: 10.1182/blood-2024-204929

[220] Caserta S Innao V Musolino C Allegra A . Immune checkpoint inhibitors in multiple myeloma: a review of the literature. Pathol Res Pract. (2020) 216:153114. doi: 10.1016/j.prp.2020.153114 32853951

[221] Liu Z Xu X Liu H Zhao X Yang C Fu R . Immune checkpoint inhibitors for multiple myeloma immunotherapy. Exp Hematol Oncol. (2023) 12:99. doi: 10.1186/s40164-023-00456-5 38017516 PMC10685608

[222] Mestiri S El-Ella DMA Fernandes Q Bedhiafi T Almoghrabi S Akbar S . The dynamic role of immune checkpoint molecules in diagnosis, prognosis, and treatment of head and neck cancers. BioMed Pharmacother. (2024) 171:116095. doi: 10.1016/j.biopha.2023.116095 38183744

[223] Kong X Zhang J Chen S Wang X Xi Q Shen H . Immune checkpoint inhibitors: breakthroughs in cancer treatment. Cancer Biol Med. (2024) 21:451–72. doi: 10.20892/j.issn.2095-3941.2024.0055 38801082 PMC11208906

[224] Murciano-Goroff YR Warner AB Wolchok JD . The future of cancer immunotherapy: microenvironment-targeting combinations. Cell Res. (2020) 30:507–19. doi: 10.1038/s41422-020-0337-2 32467593 PMC7264181

[225] Sarwar A Hashim L Faisal MS Haider MZ Ahmed Z Ahmed TF . Advances in viral oncolytics for treatment of multiple myeloma - a focused review. Expert Rev Hematol. (2021) 14:1071–83. doi: 10.1080/17474086.2021.1972802 34428997

[226] Marchica V Costa F Donofrio G Giuliani N . Oncolytic virotherapy and microenvironment in multiple myeloma. Int J Mol Sci. (2021) 22. doi: 10.3390/ijms22052259 33668361 PMC7956262

[227] Parrish C Scott GB Coffey M Melcher A Errington-Mais F Cook G . Combination therapy with reovirus and immunomodulatory drugs induces direct oncolytic and immune-mediated killing of multiple myeloma cells and overcomes stromal-mediated microenvironmental protection. Blood. (2014) 124:4778. doi: 10.1182/blood.v124.21.4778.4778

[228] Kelly KR Espitia CM Zhao W Wu K Visconte V Anwer F . Oncolytic reovirus sensitizes multiple myeloma cells to anti-PD-L1 therapy. Leukemia. (2018) 32:230–3. doi: 10.1038/leu.2017.272 28832023 PMC5844271

[229] Yang YL Yang F Huang ZQ Li YY Shi HY Sun Q . T cells, NK cells, and tumor-associated macrophages in cancer immunotherapy and the current state of the art of drug delivery systems. Front Immunol. (2023) 14:1199173. doi: 10.3389/fimmu.2023.1199173 37457707 PMC10348220

[230] Kyrysyuk O Wucherpfennig KW . Designing cancer immunotherapies that engage T cells and NK cells. Annu Rev Immunol. (2023) 41:17–38. doi: 10.1146/annurev-immunol-101921-044122 36446137 PMC10159905

[231] van Elsas MJ Middelburg J Labrie C Roelands J Schaap G Sluijter M . Immunotherapy-activated T cells recruit and skew late-stage activated M1-like macrophages that are critical for therapeutic efficacy. Cancer Cell. (2024) 42:1032–50. doi: 10.1016/j.ccell.2024.04.011 38759656

[232] Wang X Yang X Wang Y Chen Y Yang Y Shang S . Combination of expanded allogeneic NK cells and T cell-based immunotherapy exert enhanced antitumor effects. Cancers (Basel). (2022) 15. doi: 10.3390/cancers15010251 36612246 PMC9818244

[233] Bai G Sun C Guo Z Wang Y Zeng X Su Y . Accelerating antibody discovery and design with artificial intelligence: recent advances and prospects. Semin Cancer Biol. (2023) 95:13–24. doi: 10.1016/j.semcancer.2023.06.005 37355214

[234] Matsunaga R Tsumoto K . Accelerating antibody discovery and optimization with high-throughput experimentation and machine learning. J BioMed Sci. (2025) 32:46. doi: 10.1186/s12929-025-01141-x 40346589 PMC12063268

[235] Kim J McFee M Fang Q Abdin O Kim PM . Computational and artificial intelligence-based methods for antibody development. Trends Pharmacol Sci. (2023) 44:175–89. doi: 10.1016/j.tips.2022.12.005 36669976

[236] Papadimitriou M Ahn S Diamond BT Lee H McIntyre JB Truger M . Timing antigenic escape in multiple myeloma treated with T-cell redirecting immunotherapies. bioRxiv. (2024) 144:1899. doi: 10.1101/2024.05.22.595383 40906971 PMC12514755

