# Supplementary material for: Emerging precision medicine in multiple myeloma: clinical and preclinical landscape of T cell, natural killer cell, and macrophages engaging multi-specific antibodies
Source: Front Immunol. 2026 Jul 7;17:1822508. doi: 10.3389/fimmu.2026.1822508 (PMC13385978; doi:10.3389/fimmu.2026.1822508)
Supplement: Supplementary file 1 [file Supplementaryfile1.docx]

**Table 3: Clinical trial studies on combination therapies to overcome toxicity and resistance to T and NK and macrophage engager multi-specific antibodies in multiple myeloma based on** [**ClincalTrials.gov**](https://clinicaltrials.gov/)

| Clinical Trial identifier | Status | Phase | Interventions | Target Tested | Tumor Type | Primary Outcome Measures/ Time frame | Enrolment | Published results |
| --- | --- | --- | --- | --- | --- | --- | --- | --- |
| NCT03269136 | Completed | 1 | Elranatamab monotherapy  Elranatamab +  dexamethasone  lenalidomide  pomalidomide | BCMA-CD3 Ab  Immunomodulatory agents | RRMM | Safety and tolerability at increasing dose levels of Elranatamab to determine the maximum tolerated dose and select the recommended Phase II dose. | 101 | (1-3) |
| NCT05090566 | Recruiting | 2 | Elranatamab + Nirogacestat  Elranatamab + lenalidomide + dexamethasone | BCMA-CD3 Ab  γ-Secretase Inhibitor  Immunomodulatory agents | RRMM | Determine the recommended Phase II dose and clinical benefit of Elranatamab in combination with other anti-cancer therapies. | 46 |  |
| NCT06645678 | Recruiting | 1/2 | Elranatamab + mezigdomide + dexamethasone | BCMA-CD3 Ab  Cereblon (CRBN) E3 ligase modulator  Immunomodulatory agents | RRMM | Efficacy and safety of Elranatamab in combination with mezigdomide. | 75 |  |
| NCT05927571 | Recruiting | 1 | Elranatamab + Cevostamab | BCMA-CD3 Ab  FCRL5-CD3 Ab | RRMM | Safety and tolerability of Cevostamab + Elranatamab and determine the recommended Phase II dose | 120 |  |
| NCT06215118 | Recruiting | 1 | Elranatamab + Iberdomide | BCMA-CD3 Ab  Cereblon (CRBN) E3 ligase modulator | RRMM | Tolerability and safety of dosesof elranatamab + Iberdomide. | 87 |  |
| NCT05675449 | Recruiting | 1 | Elranatamab + Carfilzomib + Dexamethasone  Elranatamab + Maplirpacept | BCMA-CD3 Ab  Proteasome inhibitor  Immunomodulatory agents  SIRPαFc fusion protein (CD47-Blocking Immune Checkpoint Inhibitor) | RRMM | Safety and tolerability, and optimal dose of elranatamab + Maplirpacept. | 59 |  |
| NCT05020236 | Active, not recruiting | 3 | Elranatamab monotherapy  Elranatamab +  Daratumumab  Daratumumab + Pomalidomide + Dexamethasone | BCMA-CD3 Ab  Anti-CD38 mAb  Immunomodulatory agents | RRMM | Compare benefit of Elranatamab alone or in combination with daratumumab to the combination of daratumumab, pomalidomide, and dexamethasone. | 944 |  |
| NCT05137054 | Recruiting | 1 | Linvoseltamab +  Daratumumab  Carfilzomib  Lenalidomide  Bortezomib  Pomalidomide  Isatuximab  Fianlimab  Cemiplimab  Nirogacestat | BCMA-CD3 Ab  Anti-CD38  Proteosome inhibitor  Immune checkpoint inhbitor  γ-Secretase nhibitor  Immunomodulatory agents | RRMM | Safety of Linvoseltamab in combination with other treatments and its dosage in combination therapies. | 317 |  |
| NCT07312188 | Recruiting |  | F182112 monotherapy  F182112 + Anti-CD38 mAb | BCMA-CD3 Ab  Anti-CD38 mAb | RRMM | The efficacy and safety of F182112 combined with anti-CD38 mAb. | 90 |  |
| NCT04586426 | Active, not recruiting | 1/2 | Teclistamab + Talquetamab    Teclistamab + Talquetamab + Daratumumab | GPRC5D-CD3 Ab  Anti-CD38 mAb | RRMM | Safety and anticancer activity of Talquetamab + Teclistamab without or with Daratumumab | 228 | (4-6) |
| NCT04108195 | Active, not recruiting | 1 | Daratumumab +  Teclistamab  Talquetamab  Talquetamab + Pomalidomide  Teclistamab + Pomalidomide | GPRC5D-CD3 Ab  BCMA-CD3 Ab  Anti-CD38 | RRMM | Safety of Daratumumab, with or without Pomalidomide, in combination with Talquetamab and Teclistamab, and their recommended phase 2 doses. | 290 | (7, 8) |
| NCT06100237 | Recruiting | 2 | Daratumumab + Teclistamab  Daratumumab + Talquetamab | GPRC5D-CD3 Ab  BCMA-CD3 Ab  Anti-CD38 mAb | High-Risk SMM | Efficacy of Daratumumab with Teclistamab or Talquetamab | 50 |  |
| NCT04722146 | Active, not recruiting | 1 | Teclistamab + Daratumumab + Pomalidomide  Teclistamab + Daratumumab + Lenalidomide + Bortezomib  Teclistamab + Nirogacestat  Teclistamab + Lenalidomide  Teclistamab + Daratumumab + Lenalidomide  Teclistamab + Daratumumab + Lenalidomide + Bortezomib | GPRC5D-CD3 Ab  BCMA-CD3 Ab  Immunomodulatory agents  Anti-CD38 mAb  γ-Secretase Inhibitor | RRMM  NDMM | Assess the safety and tolerability of teclistamab when administered in different combination regimen and to identify the optimal dose(s) of teclistamab combination regimens. | 140 |  |
| NCT05083169 | Active, not recruiting | 3 | Teclistamab + Daratumumab  Daratumumab + Pomalidomide + Dexamethasone  Daratumumab + Bortezomib + Dexamethasone | GPRC5D-CD3 Ab  BCMA-CD3 Ab  Immunomodulatory agents  Anti-CD38 mAb  Proteosome inhibitor | RRMM | Evaluate the efficacy of teclistamab- daratumumab with daratumumab in combination with pomalidomide and dexamethasone or daratumumab in combination with bortezomib and dexamethasone | 587 | (9) |
| NCT07105059 | Recruiting | 1 | Teclistamab + Mezigdomide | BCMA-CD3 Ab  Mezigdomide | RRMM | Evaluate the safety and efficacy of teclistamab in combination with mezigdomide. | 18 |  |
| NCT06880601 | Not yet recruiting | 2 | Teclistamab + autologous lymphocyte infusions | BCMA-CD3 Ab  Autologous lymphocyte infusions | RRMM | Evaluate the safety and efficacy of teclistamab and autologous lymphocyte infusions in relapse refractory multiple myeloma. | 52 |  |
| NCT05552222 | Recruiting | 3 | Teclistamab + Daratumumab + Lenalidomide  Talquetamab + Daratumumab + Lenalidomide  Daratumumab + Dexamethasone + Lenalidomide | GPRC5D-CD3 Ab  BCMA-CD3 Ab  Immunomodulatory agents  Anti-CD38 mAb | NDMM | Evaluate the efficacy of teclistamab in combination with daratumumab and lenalidomide and talquetamab in combination with daratumumab and lenalidomide versus daratumumab, lenalidomide, dexamethasone. | 1590 |  |
| NCT06353022 | Recruiting | 2 | Teclistamab + Lenalidomide  Talquetamab + Teclistamab | GPRC5D-CD3 Ab  BCMA-CD3 Ab | NDMM | Investigate teclistamab in combination with lenalidomide or in combination with talquetamab, allocated based on minimal residual disease (MRD) status standard-risk versus MRD high-risk respectively. | 103 |  |
| NCT05695508 | Recruiting | 2 | Several Teclistamab- and Talquetamab-based combination regimens | GPRC5D-CD3 Ab  BCMA-CD3 Ab  Immunomodulatory agents  Anti-CD38 mAb  Proteasome inhibitor | NDMM and Transplant Eligible | Safety and efficacy of teclistamab- and talquetamab-based combination regimens in participants with newly diagnosed and transplant eligible MM. | 160 |  |
| NCT05338775 | Active, not recruiting | 1 | Teclistamab or Talquetamab with PD-1 inhibitor | GPRC5D-CD3 Ab  BCMA-CD3 Ab  PD-1 inhibitor | RRMM | Safe dose(s) and tolerability of combination of a PD-1 inhibitor in combination with talquetamab or teclistamab | 74 |  |
| NCT06588660 | Terminated | 1 | Teclistamab + Vevoctadekin (ST-067) | BCMA-CD3 Ab  human cytokine interleukin-18 | RRMM | Safety, side effects and best dose of ST-067 in combination with teclistamab | 1 |  |
| NCT06465316 | Recruiting | 1 | Teclistamab + Iberdomide | BCMA-CD3 Ab  Cereblon (CRBN) E3 ligase modulators | RRMM | Safety, side effects, and best dose of iberdomide in combination with teclistamab | 26 |  |
| NCT05849610 | Active, not recruiting | 2 | DVRd induction (Daratumumab + Bortezomib + Lenalidomide + Dexamethasone) then:  Teclistamab + Daratumumab (Tec-Dara)  Talquetamab + Daratumumab If MRD or no CR despite MRD negative after Tec-Dara | GPRC5D-CD3  BCMA-CD3  Immunomodulatory agents  Anti-CD38 mAb  Proteasome inhibitor | High-Risk de Novo MM | Evaluate the efficacy and safety of Teclistamab + Daratumumab (Tec-Dara) and Talquetamab + Daratumumab (Tal-Dara) in terms of measurable residual disease negative complete remission rate by next generation flow cytometry in de novo high-risk multiple myeloma patients. | 30 |  |
| NCT06577025 | Recruiting | 2 | DVRd induction (Daratumumab + Bortezomib + Lenalidomide + Dexamethasone) then:    Talquetamab + Daratumumab (Tal-D) + Cilta-cel  OR  Cilta-cel + Tal-D and Teclistamab + Daratumumab (Tec-D) | GPRC5D-CD3 Ab  BCMA-CD3 Ab  Immunomodulatory agents  Anti-CD38 mAb  BCMA-CART  Proteasome inhibitor  Anti-BCMA CAR T cell | NDMM | Rate of response with signs of potential cure at 5 years after the start of induction treatment in terms of measurable MRD negativity with complete response. | 43 |  |
| NCT06208150 | Recruiting | 3 | Talquetamab + Pomalidomide  Talquetamab + Teclistamab  Pomalidomide + Elotuzumab + Bortezomib | GPRC5D-CD3 Ab  BCMA-CD3 Ab  Anti-SLAMF7  Immunomodulatory agents  Proteasome inhibitor | RRMM | Effectiveness of either talquetamab + pomalidomide or talquetamab + teclistamab with elotuzumab, pomalidomide, and bortezomib | 795 |  |
| NCT05243797 | Recruiting | 3 | Teclistamab  Lenalidomide  Teclistamab + Lenalidomide | BCMA-CD3  Immunomodulatory agent | NDMM | Benefits of teclistamab in combination with lenalidomide and teclistamab after autologous stem cell transplant. | 1594 |  |
| NCT05572229 | Recruiting | 2 | Teclistamab + Daratumumab  Teclistamab + Lenalidomide | BCMA-CD3  Anti-CD38 mAb  Immunomodulatory agent | NDMM | Efficacy and safety of the combination teclistamab + daratumumab  or teclistamab + lenalidomide  in patients with newly diagnosed multiple myeloma who are not eligible for SCT. | 74 |  |
| NCT06461988 | Not yet recruiting | 2 | Talquetamab + Lenalidomide | GPRC5D-CD3  immunomodulatory agent | MM | Evaluate the efficacy of talquetamab and lenalidomide as post stem cell transplant maintenance in multiple myeloma | 20 |  |
| NCT03601078 | Active, not recruiting | 2 | BB2121  BB2121 + ASCT  BB2121 – ASCT  BB2121 with inadequate response to ASCT  BB2121+ Talquetamab  BB2121 + Lenalidomide | Anti-BCMA CAR T cell  GPRC5D-CD3  Immunomodulatory agent | RRMM | Efficacy and safety of BB2121 patients receiving bridging therapy with talquetamab. Efficacy and safety of BB2121 used in combination with lenalidomide maintenance in participants with suboptimal response post ASCT. | 312 |  |
| NCT06348108 | Not yet recruiting | 1 | Talquetamab + Iberdomide + Dexamethasone | GPRC5D-CD3 Ab  Cereblon (CRBN) E3 ligase modulators  Immunomodulatory agents | RRMM | Safety and toxicity of the combination of talquetamab, iberdomide and dexamethasone in patients with triple class exposed multiple myeloma | 32 |  |
| NCT05050097 | Active, not recruiting | 1 | Talquetamab +  Carfilzomib  Pomalidomide  Lenalidomide  Daratumumab + Carfilzomib  Daratumumab + Lenalidomide | GPRC5D-CD3 Ab  Proteosome inhibitor  Immunomodulatory agents  Anti-CD38 mAb | MM | Safety and tolerability of talquetamab when administered in different combination regimens and to identify the safe dose(s) of talquetamab combination regimens. | 166 |  |
| NCT06550895 | Recruiting | 2 | Cilta-cel + Talquetamab consolidation post CAR-T therapy | GPRC5D-CD3 Ab  Anti-BCMA CAR T cell | High-Risk MM | Safety of ciltacabtagene autoleucel and talquetamab | 11 |  |
| NCT05455320 | Recruiting | 3 | Talquetamab + Daratumumab + Pomalidomide  Talquetamab + Daratumumab  Daratumumab + Pomalidomid | GPRC5D-CD3 Ab  Immunomodulatory agents  Anti-CD38 mAb | RRMM | Compare the efficacy of talquetamab in combination with daratumumab and pomalidomide to other combinations. | 864 |  |
| NCT06572605 | Not yet recruiting | 1/2 | Extramedullary disease (EMD)-directed external beam radiotherapy (EMD-EBRT) + Talquetamab | GPRC5D-CD3  Radiation therapy | MM with Extramedullary Disease | Safety and tolerability of single-field, palliative, extramedullary disease (EMD)-directed external beam radiotherapy (EMD-EBRT) in combination with talquetamab | 20 |  |
| NCT06669247 | Recruiting | 1/2 | Linvoseltamab monotherapy  Linvoseltamab + REGN7945 | BCMA-CD3 Ab  CD38-CD28 costimulatory BsAb | RRMM | Safety, tolerability, and preliminary anti-tumor activity of REGN7945, in combination with linvoseltamab | 186 |  |
| NCT05137054 | Recruiting | 1 | Linvoseltamab +  Daratumumab  Carfilzomib  Lenalidomide  Bortezomib  Pomalidomide  Isatuximab  Fianlimab  Cemiplimab  Nirogacestat | BCMA-CD3 Ab  Anti-CD38 mAb  Proteosome inhibitor  Immunomodulatory agent  Immune checkpoint inhibitor  γ-Secretase Inhibitor | RRMM | Safety of linvoseltamab when given in combination with other cancer treatments, and if so, what dose of linvoseltamab should be used for each combination. | 317 |  |
| NCT06121843 | Recruiting | 1 | BMS-986393 +  Alnuctamab  Mezigdomide  Iberdomide | Anti-GPRC5D CAR  T cell  BCMA-CD3 Ab  Cereblon (CRBN) E3 ligase modulators | RRMM | Safety and preliminary efficacy of BMS-986393 in combinations and determine the recommended dose | 147 |  |
| NCT06163898 | Active, not recruiting | 1 | Alnuctamab  Alnuctamab + Mezigdomide + Dexamethasone | BCMA-CD3 Ab  Cereblon (CRBN) E3 ligase modulators  Immunomodulatory agent | RRMM | Recommended dose and schedule, safety and preliminary efficacy of alnuctamab + Mezigdomide + Dexamethasone. | 156 |  |
| NCT06055075 | Active, not recruiting | 1/2 | Forimtamig + Daratumumab  Forimtamig + Carfilzomib | GPRC5D-CD3 Ab  Anti-CD38 mAb  Proteosome inhibitor | RRMM | Safety, tolerability, and preliminary anti-tumor activity of forimtamig alone or in combination with carfilzomib or daratumumab | 19 |  |
| NCT05646836 | Active, not recruiting | 1 | Cevostamab  XmAb24306 + Cevostamab | interleukin (IL)-15/IL-15 receptor α (IL-15Rα) Fc-fusion protein.  FCRL5-CD3 Ab | RRMM | Safety, pharmacokinetics, and activity of XmAb24306 in combination with cevostamab | 90 |  |
| NCT06799026 | Not yet recruiting | 1 | Elranatamab + dendritic cells/MM fusion vaccine + Granulocyte-Macrophage Colony-Stimulating Factor (GM-CSF) | BCMA-CD3  Dendritic cell vaccine | RRMM | Safety and efficacy of the combination of the Dendritic Cell/Multiple Myeloma fusion vaccine with elranatamab | 25 |  |
| NCT06588660 | Terminated | 1 | Teclistamab + ST-067 | GPRC5D-CD3  Engineered IL-18 variant | RRMM | Safety, side effects and best dose, and efficacy of ST-067 in combination with teclistamab | 1 |  |
| NCT06500884 | Recruiting | 2 | Talquetamab + 3 different prophylaxes | GPRC5D-CD3 | RRMM | Identify preventive treatments that can minimize the occurrence, severity, and duration of talquetamab-related taste changes (dysgeusia) | 210 |  |

Multiple Myeloma (MM), Relapsed Refractory Multiple Myeloma (RRMM), B-cell Maturation Antigen (BCMA), Fc receptor-homolog 5 (FCRL5), Monoclonal Antibody (mAb), Chimeric Antigen Receptor (CAR), Newly Diagnosed Multiple Myeloma (NDMM), Recommended Phase 2 dose(s) (RP2Ds), Bispecific Antibody (BsAb), Minimal Residual Disease (MRD), Autologous Stem Cell Transplant (ASCT), Smoldering Multiple Myeloma (SMM), International Myeloma Working Group Response Criteria (IMWG).

**References**

1. Lon HK, Hibma J, Jiang S, Sullivan S, Vandendries E, Skoura A, et al. Population Exposure-Response Efficacy Analysis of Elranatamab (PF-06863135) in Patients with Multiple Myeloma. Target Oncol. 2025;20(5):803-19.

2. Elmeliegy M, Viqueira A, Vandendries E, Hickman A, Conte U, Irby D, et al. Dose Optimization of Elranatamab to Mitigate the Risk of Cytokine Release Syndrome in Patients with Multiple Myeloma. Target Oncol. 2025;20(2):349-59.

3. Bahlis NJ, Costello CL, Raje NS, Levy MY, Dholaria B, Solh M, et al. Elranatamab in relapsed or refractory multiple myeloma: the MagnetisMM-1 phase 1 trial. Nature Medicine. 2023;29(10):2570-6.

4. Kumar S, Mateos MV, Ye JC, Atrash S, Magen H, Quach H, et al. Dual Targeting of Extramedullary Myeloma with Talquetamab and Teclistamab. N Engl J Med. 2026;394(1):51-61.

5. Cohen YC, Magen H, Gatt M, Sebag M, Kim K, Min CK, et al. Talquetamab plus Teclistamab in Relapsed or Refractory Multiple Myeloma. N Engl J Med. 2025;392(2):138-49.

6. St Martin Y, Franz JK, Agha ME, Lazarus HM. Failure of CAR-T cell therapy in relapsed and refractory large cell lymphoma and multiple myeloma: An urgent unmet need. Blood Rev. 2023;60:101095.

7. Chari A, van de Donk N, Dholaria B, Weisel K, Mateos MV, Goldschmidt H, et al. Talquetamab plus daratumumab for the treatment of relapsed or refractory multiple myeloma in the TRIMM-2 study. Blood. 2025;146(24):2902-13.

8. Pillarisetti K, Powers G, Luistro L, Babich A, Baldwin E, Li Y, et al. Teclistamab is an active T cell-redirecting bispecific antibody against B-cell maturation antigen for multiple myeloma. Blood Adv. 2020;4(18):4538-49.

9. Costa LJ, Bahlis NJ, Perrot A, Nooka AK, Lu J, Pawlyn C, et al. Teclistamab plus Daratumumab in Relapsed or Refractory Multiple Myeloma. N Engl J Med. 2026;394(8):739-52.
